# Supplementary material for: Cardiac ryanodine receptor distribution is dynamic and changed by auxiliary proteins and post-translational modification
Source: eLife. 2020 Jan 9;9:e51602. doi: 10.7554/eLife.51602 (PMC6994221; doi:10.7554/eLife.51602)
Supplement: Figure 1—source data 1. [file elife-51602-fig1-data1.pdf]

Figure 1 – Source Data 1

A) Quartiles

Normalized Spark Frequency – Quartiles

Condition	0%	25%	50%	75%	100%
Control	0.33	0.65	0.98	1.31	1.64
Phos	1.07	1.52	2.77	3.67	5.12
FKBP12	0.00	0.12	0.27	0.36	0.84
FKBP12 + Phos	0.74	1.47	1.84	2.57	2.94
FKBP12.6	0.00	0.27	0.41	0.54	1.08
FKBP12.6 + Phos	2.27	3.33	3.97	4.54	5.10

Amplitude - Quartiles

Condition	0%	25%	50%	75%	100%
Control	0.35	0.65	0.98	1.63	4.11
Phos	0.42	0.92	1.27	1.82	3.61
FKBP12	0.35	0.61	0.81	1.20	3.99
FKBP12 + Phos	0.46	0.83	1.31	2.00	4.11
FKBP12.6	0.33	0.52	0.77	1.64	3.08
FKBP12.6 + Phos	0.35	0.82	1.47	1.77	4.08

Full Duration Half Maximum (FDHM) - -Quartiles

Condition	0%	25%	50%	75%	100%
Control	5.70	12.35	16.14	19.23	36.09
Phos	5.70	12.35	17.09	23.27	51.28
FKBP12	3.80	9.50	11.88	14.25	30.39
FKBP12 + Phos	3.80	12.59	16.14	20.89	34.19
FKBP12.6	6.65	9.74	14.25	18.04	51.28
FKBP12.6 + Phos	1.90	17.09	22.79	28.49	78.82

Full Width Half Maximum (FWHM) - Quartiles

Condition	0%	25%	50%	75%	100%
Control	0.99	2.13	2.41	2.84	3.98
Phos	1.14	2.13	2.56	2.84	4.69
FKBP12	0.71	1.70	2.13	2.41	3.55
FKBP12 + Phos	0.99	1.85	2.13	2.41	4.26
FKBP12.6	0.99	1.56	1.70	1.99	3.12
FKBP12.6 + Phos	0.57	1.99	2.27	2.56	4.97

Spark Mass - Quartiles

Condition	0%	25%	50%	75%	100%
Control	3.82	19.66	37.29	66.00	212.35
Phos	10.56	31.87	53.60	90.38	458.37
FKBP12	1.82	10.63	22.81	36.74	330.93
FKBP12 + Phos	2.01	28.24	46.69	70.90	188.15
FKBP12.6	3.12	10.36	19.48	51.30	127.42
FKBP12.6 + Phos	0.52	28.42	70.39	109.99	711.09

B) Spark Parameters – Statistical Analyses

**Spark Freq/Ca Load**	HDA		Holm-Bonferroni	
	p Value	Bonferroni sig.	Critical p Value	Null Hypothesis
Control - FKBP12	1.93957E-03	*	0.0250	REJECT
Control - FKBP12.6	1.78197E-03	*	0.0167	REJECT
Control - FKBP12.6 + Phos	8.14389E-22	***	0.0042	REJECT
Control - FKBP12 + Phos	5.88808E-06	***	0.0083	REJECT
Control - Phosph	5.58734E-14	***	0.0050	REJECT
FKBP12.6 - FKBP12.6 + Phos	3.28820E-29	***	0.0033	REJECT
FKBP12 + Phos - FKBP12.6	1.13080E-12	***	0.0071	REJECT
FKBP12 + Phos - FKBP12.6 + Phos	9.57428E-13	***	0.0056	REJECT
FKBP12 - FKBP12.6	8.04323E-01		0.0500	
FKBP12 - FKBP12.6 + Phos	2.60691E-26	***	0.0036	REJECT
FKBP12 - FKBP12 + Phos	1.08997E-12	***	0.0063	REJECT
Phos - FKBP12	6.75885E-22	***	0.0038	REJECT
Phos - FKBP12+Phos	1.88772E-04	**	0.0125	REJECT
Phos - FKBP12.6	9.55791E-22	***	0.0045	REJECT
Phos - FKBP12.6+Phos	1.37412E-05	***	0.0100	REJECT

**Log Amplitude**	HDA		Holm-Bonferroni	
	p Value	Bonferroni sig.	Critical p Value	Null Hypothesis
Control - FKBP12	1.04861E-05	***	0.0038	REJECT
Control - FKBP12 + Phos	9.31841E-01		0.0500	
Control - FKBP12.6	9.11395E-05	**	0.0050	REJECT
Control - FKBP12.6 + Phos	5.01714E-01		0.0167	
Control - Phos	4.50906E-01		0.0100	
FKBP12 - FKBP12 + Phos	7.55375E-06	***	0.0033	REJECT
FKBP12 - FKBP12.6	8.32945E-01		0.0250	
FKBP12 - FKBP12.6 + Phos	8.67841E-06	***	0.0036	REJECT
FKBP12 + Phos - FKBP12.6	3.58709E-05	***	0.0045	REJECT
FKBP12 + Phos - FKBP12.6 + Phos	4.28549E-01		0.0083	
FKBP12.6 - FKBP12.6 + Phos	1.32935E-05	***	0.0042	REJECT
Phos - FKBP12	1.18239E-04	**	0.0056	REJECT
Phos - FKBP12 + Phos	4.60517E-01		0.0125	
Phos - FKBP12.6	4.84750E-04	**	0.0063	REJECT
Phos - FKBP12.6 + Phos	1.75157E-01		0.0071	

Signif. Codes: Bonferroni $p < 0.0001$ '***'; $p < 0.001$ '**'; $p < 0.05$ '*'

	HDA		Holm-Bonferroni	
**Log FWHM**	p Value	Bonferroni sig.	Critical p Value	Null Hypothesis
Control - FKBP12	1.36242E-05	***	0.0045	REJECT
Control - FKBP12 + Phos	9.72653E-03		0.0071	
Control - FKBP12.6	6.04275E-07	***	0.0038	REJECT
Control - FKBP12.6 + Phos	8.42478E-01		0.0500	
Control - Phos	2.31312E-01		0.0125	
FKBP12 - FKBP12 + Phos	2.71783E-02		0.0100	
FKBP12 - FKBP12.6	3.13378E-01		0.0167	
FKBP12 - FKBP12.6 + Phos	5.99562E-05	***	0.0050	REJECT
FKBP12 + Phos - FKBP12.6	1.29424E-03	*	0.0063	REJECT
FKBP12 + Phos - FKBP12.6 + Phos	1.07433E-02		0.0083	
FKBP12.6 - FKBP12.6 + Phos	6.70286E-07	***	0.0042	REJECT
Phos - FKBP12	5.85344E-08	***	0.0036	REJECT
Phos - FKBP12 + Phos	8.52244E-05	**	0.0056	REJECT
Phos - FKBP12.6	8.95653E-10	***	0.0033	REJECT
Phos - FKBP12.6 + Phos	3.81428E-01		0.0250	

	HDA		Holm-Bonferroni	
**Log FDHM**	p Value	Bonferroni sig.	Critical p Value	Null Hypothesis
Control - FKBP12	3.70342E-03		0.0063	REJECT
Control - FKBP12 + Phos	1.83813E-01		0.0167	
Control - FKBP12.6	6.03849E-01		0.0500	
Control - FKBP12.6 + Phos	2.22245E-06	***	0.0042	REJECT
Control - Phos	2.79745E-02		0.0083	
FKBP12 - FKBP12 + Phos	3.21064E-05	***	0.0045	REJECT
FKBP12 - FKBP12.6	3.01389E-02		0.0100	
FKBP12 - FKBP12.6 + Phos	5.45550E-11	***	0.0033	REJECT
FKBP12 + Phos - FKBP12.6	7.44477E-02		0.0125	
FKBP12 + Phos - FKBP12.6 + Phos	1.08502E-04	**	0.0050	REJECT
FKBP12.6 - FKBP12.6 + Phos	3.49299E-07	***	0.0036	REJECT
Phos - FKBP12	1.07112E-06	***	0.0038	REJECT
Phos - FKBP12 + Phos	3.24937E-01		0.0250	
Phos - FKBP12.6	9.77440E-03		0.0071	
Phos - FKBP12.6 + Phos	2.63050E-03	*	0.0056	REJECT

Signif. Codes: Bonferroni $p < 0.0001$ ‘***’; $p < 0.001$ ‘**’; $p < 0.05$ ‘*’

**Log Spark Mass**	HDA		Holm-Bonferroni	
	p Value	Bonferroni sig.	Critical p Value	Null Hypothesis
Control - FKBP12	6.79403E-07	***	0.0045	REJECT
Control - FKBP12 + Phos	8.77939E-01		0.0500	
Control - FKBP12.6	1.68780E-04	**	0.0063	REJECT
Control - FKBP12.6 + Phos	7.00849E-03		0.0083	REJECT
Control - Phos	5.05752E-01		0.0250	
FKBP12 - FKBP12 + Phos	5.92590E-07	***	0.0042	REJECT
FKBP12 - FKBP12.6	3.53118E-01		0.0125	
FKBP12 - FKBP12.6 + Phos	1.21465E-10	***	0.0033	REJECT
FKBP12 + Phos - FKBP12.6	8.84271E-05	**	0.0056	REJECT
FKBP12 + Phos - FKBP12.6 + Phos	2.92968E-03	*	0.0071	REJECT
FKBP12.6 - FKBP12.6 + Phos	4.03930E-09	***	0.0036	REJECT
Phos - FKBP12	2.92260E-08	***	0.0038	REJECT
Phos - FKBP12 + Phos	3.71282E-01		0.0167	
Phos - FKBP12.6	6.94202E-06	***	0.0050	REJECT
Phos - FKBP12.6 + Phos	2.88229E-02		0.0100	

Signif. Codes: Bonferroni p < 0.0001 ‘***’; p < 0.001 ‘**’; p < 0.05 ‘*’
