# Supplementary material for: Cardiac ryanodine receptor distribution is dynamic and changed by auxiliary proteins and post-translational modification
Source: eLife. 2020 Jan 9;9:e51602. doi: 10.7554/eLife.51602 (PMC6994221; doi:10.7554/eLife.51602)
Supplement: Figure 1—source data 2. [file elife-51602-fig1-data2.docx]

**Figure 1 – source data 2**

| **Experimental Group** | **Sparks Analysed** | **Cells** | **Rats** |
| --- | --- | --- | --- |
| Control | 167 | 25 | 3 |
| Phosphorylation cocktail | 239 | 20 | 4 |
| FKBP12 | 99 | 31 | 4 |
| FKBP12 + phosphorylayion cocktail | 186 | 20 | 3 |
| FKBP12.6 | 77 | 23 | 4 |
| FKBP12.6 + phosphorylation cocktail | 187 | 15 | 3 |
