# Supplementary material for: Cardiac ryanodine receptor distribution is dynamic and changed by auxiliary proteins and post-translational modification
Source: eLife. 2020 Jan 9;9:e51602. doi: 10.7554/eLife.51602 (PMC6994221; doi:10.7554/eLife.51602)
Supplement: Figure 5—source data 1. [file elife-51602-fig5-data1.pdf]

Figure 5 - Source Data 1

Statistical analysis for Figure 5: CDF plot of NNDs

Using the Anderson-Darling k sample test (corrected for ties)

NNDs between tetramers
All Groups: p = 0 - SIGNIFICANT
Control vs phosphorylated: p = 0.0063183 - SIGNIFICANT
Control vs FKBP12: p = 1.498e-26 - SIGNIFICANT
Control vs FKBP12.6: p = 0 - SIGNIFICANT
Control vs FKBP12 phos: p = 0.15442 - NS
Control vs FKBP12.6 phos: p = 2.7804e-11 - SIGNIFICANT
FKBP12 vs FKBP12 phos: p = 3.9539e-79 - SIGNIFICANT
FKBP12.6 vs FKBP12.6 phos: p = 0 - SIGNIFICANT
FKBP12 vs FKBP12.6: p = 8.153e-10 - SIGNIFICANT
FKBP12 phos vs FKBP12.6 phos: p = 0 - SIGNIFICANT
Phosphorylated vs FKBP12 phos: p = 0.20261 - NS
Phosphorylated vs FKBP12.6 phos: p = 0.022621 - SIGNIFICANT
