# Supplementary material for: Cardiac ryanodine receptor distribution is dynamic and changed by auxiliary proteins and post-translational modification
Source: eLife. 2020 Jan 9;9:e51602. doi: 10.7554/eLife.51602 (PMC6994221; doi:10.7554/eLife.51602)
Supplement: Figure 6—source data 1. [file elife-51602-fig6-data1.pdf]

Figure 6 - Source Data 1

Statistical analysis for Figure 6C: CDF plot of Alpha Shape Areas

Using the Anderson-Darling k sample test (corrected for ties)

Alpha Shape Areas
All Groups: p = 0 - SIGNIFICANT
Control vs phosphorylated: p = 1.0904e-26 - SIGNIFICANT
Control vs FKBP12: p = 0 - SIGNIFICANT
Control vs FKBP12.6: p = 0 - SIGNIFICANT
Control vs FKBP12 phos: p = 5.9079e-146 - SIGNIFICANT
Control vs FKBP12.6 phos: p = 1.0261e-19 - SIGNIFICANT
FKBP12 vs FKBP12 phos: p = 0 - SIGNIFICANT
FKBP12.6 vs FKBP12.6 phos: p = 0 - SIGNIFICANT
FKBP12 vs FKBP12.6: p = 0.38377 - NS
FKBP12 phos vs FKBP12.6 phos: p = 0 - SIGNIFICANT
Phosphorylated vs FKBP12 phos: p = 6.9547e-05 - SIGNIFICANT
Phosphorylated vs FKBP12.6 phos: p = 0.2336 - NS
