# Supplementary material for: Cardiac ryanodine receptor distribution is dynamic and changed by auxiliary proteins and post-translational modification
Source: eLife. 2020 Jan 9;9:e51602. doi: 10.7554/eLife.51602 (PMC6994221; doi:10.7554/eLife.51602)
Supplement: Figure 6—source data 2. [file elife-51602-fig6-data2.pdf]

Figure 6 - Source Data 2

Statistical Analysis for Figure 6E – CDFs of Tetramers Per Cluster

Using the Anderson-Darling k sample test (corrected for ties)

Tetramers per Cluster
All Groups : p = 0 - SIGNIFICANT
Control vs phosphorylated: p = 1.414e-128 - SIGNIFICANT
Control vs FKBP12: p = 0 - SIGNIFICANT
Control vs FKBP12.6: p = 0 - SIGNIFICANT
Control vs FKBP12 phos: p = 4.0762e-13 - SIGNIFICANT
Control vs FKBP12.6 phos: p = 7.9987e-05 - SIGNIFICANT
FKBP12 vs FKBP12 phos: p = 0 - SIGNIFICANT
FKBP12.6 vs FKBP12.6 phos: p = 0 - SIGNIFICANT
FKBP12 vs FKBP12.6: p = 2.8133e-12 - SIGNIFICANT
FKBP12 phos vs FKBP12.6 phos: p = 0.041946 - SIGNIFICANT
Phosphorylated vs FKBP12 phos: p = 1.4053e-120 - SIGNIFICANT
Phosphorylated vs FKBP12.6 phos: p = 7.7567e-18 - SIGNIFICANT
