# Supplementary material for: Cardiac ryanodine receptor distribution is dynamic and changed by auxiliary proteins and post-translational modification
Source: eLife. 2020 Jan 9;9:e51602. doi: 10.7554/eLife.51602 (PMC6994221; doi:10.7554/eLife.51602)
Supplement: Figure 7—source data 1. [file elife-51602-fig7-data1.pdf]

Figure 7 - Source Data 1

p-values					
t-test /two tailed – Paired, compare to control	fkbp12	fkbp12.6	phosphorylation	fkbp12 + phosphorylation	fkbp12.6 + phosphorylation
SF6B (FKBP/RyR2)	0.011350218	0.039222629	0.0540984	0.03906	0.020219685
SF6 C (FKBP/CSQ)	0.003260149	0.000576786	0.3896027	0.019909	0.0298508

t-test /two tailed – Paired, compare to Phosphorylation	fkbp12+phosphorylation	fkbp12.6+phosphorylation
SF6B (FKBP/RyR2)	0.0426138	0.022610269
SF6 C (FKBP/CSQ)	0.0194692	0.017272928

t-test /two tailed – Paired, compare to fkbp12	fkbp12.6
SF6B (FKBP/RyR2)	0.1537099
SF6 C (FKBP/CSQ)	0.0548166

t-test /two tailed – Paired, compare to fkbp12	fkbp12 + phosphorylation
SF6B (FKBP/RyR2)	0.065443517
SF6 C (FKBP/CSQ)	0.107984018

t-test /two tailed – Paired, compare to fkbp12.6	Fkbp12.6 + phosphorylation
SF6B (FKBP/RyR2)	0.106742973
SF6 C (FKBP/CSQ)	0.454997981

p-values					
student T- Test compare to control	fkbp12	fkbp12.6	phosphorylation	phosphorylation+fkbp12	phosphorylation+fkbp12.6
SF6E (RyR2 p-S2814/CSQ)	0.381272455	0.489605	0.00112	0.044864041	0.019325
