# Supplementary material for: Cardiac ryanodine receptor distribution is dynamic and changed by auxiliary proteins and post-translational modification
Source: eLife. 2020 Jan 9;9:e51602. doi: 10.7554/eLife.51602 (PMC6994221; doi:10.7554/eLife.51602)
Supplement: Source code 1. [file elife-51602-code1.pdf]

ryr_fit is a program designed to place boxes with the correct dimensions and orientation at the positions of RyR tetramers in tomographic images of the dyads of mammalian cardiomyocytes. The images, which are in the TIFF format, consist of sequential parallel planes, betwen 0.5 and 1 nm apart, through the plane of the dyad and are generated by the Amira program (Thermo Fisher Scientific - FEI). The tetramer is represented by an open rectangle with a default width of 27 nm (which can be changed).

Tetramers are placed manually and can then be classified according to the scheme in Asghari et. al. Circ. Res. 115(2) 252-66, 2014. The program also calculates nearest neighbour distances as well as an alpha shape and a convex hull around the dyad, allowing and estimate of the area and the density (tetramers per unit area).

Usage: ryr_fit [options] -I image[1...image_n]

where:

image is one or more 2D or 3D TIFF file names. Images can be a single image,
multiple images or an image stack. Images can be 8 or 16 bit monochrome, RGB
or mapped TIFFs

If a single monochrome is displayed, the default colour is white although this can be changed.
Example:

```
ryr_fit -scale 1.2 -I cav3_ryr_c002Z*.tif
```

will display the sequential TIFFs at scale of 1.2 nm per pixel

Options:

-R   Analyse data without bringing up images
-scale   pixel size (in nm)
-W n   change tetramer width in nm (default 27)

Display options :

-M n   Magnification (def=1)
Left Mouse button - press then left/right movement moves through individual
planes from the image stacks. Left and right arrows on the
keyboard will do the same.
Right Mouse button - pops up menu

The program saves two files: tetramer2.dat which contains the tetramer poisitions, orientations and classifications and nnd2.dat which contains the nearest neighbour distance for each tetramer
The tetramer2.dat file is created on first use, saved and read in on subsequent use of the same data

Once in the program the following is a guide to adding, modifying, classifying and deleting the tetramers.

Adding and modifying tetramers

Adding a tetramer
Press the 'a' key and left click the mouse in the approximate position you want the tetramer to appear. The position of the tetramer can be moved one pixel at a time using the four arrow keys on the keyboard. The rotation is controlled by the mouse wheel. Once you are satisfied with the position press the 's' key to save the tetramer position

Modifying a tetramer
If, after other tetramers have been placed, there is conflict or overlap and you want to alter the position, press the 'm' key and left click on the desired tetramer, then proceed as before, using the arrow keys and the mouse wheel. Press the 's' key once finished

Delete a Tetramer
To delete a tetramer, press the 'd' key and left click on the desired tetramer, and it will be erased - Warning - this cannot be undone!

Classify a Tetramer
To classify a tetramer, press the 'c' key and left click on the desired tetramer, then use one of the following keys to classify it according to the scheme
c = checkerboard
s = side by side
b = both checkerboard and side by side(two or more different types abutting
the same tetramer)
i = isolated
u = unclassified (default for newly added tetramers)

By default you add a single tetramer each time you press a, however it is possible to add groups of tetramers by pressing 't' which will bring up a window with the following choices:
1. a single tetramer (default)
2. a group of 5 tetramers that follows the Lai checkerboard arrangement
3. a group of 9 tetramers that follows the Lai side-by-side arrangement
4. a group of 5 tetramers that follows a checkerboard arrangement with a user-defined overlap (nm) where overlap is the amount in nm that two abutting tetramers eclipse each other

Keys:

a - Add Tetramer
b - when classifying tetramer - classification = 'both'
c - Classify Tetramer
- when classifying tetramer - classification = 'checkerboard'
d - Delete Tetramer
h - Show/Hide EM Image (toggle)
i - when classifying tetramer - classification = 'isolated'
k - If look ahead is on - Minimum/Summed Intensity for each pixel (toggle)
l - Look ahead On/Off (default 4 planes)
1,2...9 - If Look Ahead is on show 1 to 9 extra planes at a time
m - Modify Tetramer
n - Show/Hide Nearest Neighbours distances (toggle)
p - Measure Distance - press p then left click and drag mouse between points
q - Hide/Unhide Frame No. (toggle)
r - Show/Hide Alpha Shape & Hull(toggle)
s - Save Tetramer after addition or mdoification
- when classifying tetramer - classification = 'side-by-side'
t - Change Tetramer placement from single to one of different groups
u - when classifying tetramer - classification = 'unclassified'
w - Change tetramer width
z - Toggle Frame display
F1 - Red/Colored Tetramers (toggle)
F2 - Show/Hide Tetramers (toggle)

F3 - Circular/Square Shadow (toggle)
F4 - Show/Hide Tetramer Shadow (toggle)
F5 - Outline Region (draw contour) - can create sub-regions of the dya
F6 – Display Scale Bar (size in nm) (toggle)
F7 - FloodFill = Surround/Complete (toggle)
F9 - Isolate Region
F10 - Print help
F11 - Change Tetramer Width
F12 - Flip Image
+ - Zoom In
- - Zoom Out
Home - move image to 1st plane
End - move image to last plane
Esc – Quit program - while drawing quit drawing and erase line
Arrow keys (left, right, up and down) - adding or modifying the tetramer keys will move the tetramer 1 pixel in the direction pressed
When viewing the image, the left arrow will move the image 1 plane back, while the right arrow will move it 1 plane forward

Mouse wheel - when adding or modifying rotate tetramers
To draw contours to isolate a region mouse middle button down and then trace path

Many of the above can be accessed through the menu (mouse right click)
For monochrome images there are colour options that can only be accessed through the menu.

The code consists of

ryr_fit3_main.cpp – startup and brief info on how to use the command line
ryr_fit3a.cpp – program to add/modify/classify the tetramers, calculate NNDs and alpha shape & hull.
scale.cpp – Allows modification of the intensity of channels within the image.
placement.cpp – Allows changing from single to group entry of the tetramers.
tiff.cpp – reads TIFF images generated by Amira – uses libtiff & tiff header files supplied by the system

There are also associated header for the bottom four cpp files, two helper header files: newscroll.h and imstruct.h, a Qt ui file: placement.ui as well as a Qt pro file to compile the program with Qt.

The program runs under Linux with Qt 4.7, CGAL libtiff and legacy OpenGL.

****** ryr_fit3_main.cpp ******

```
/*****************************************************************************************************
* ryr_fit is a program designed to place boxes with the correct dimensions and orientation
* at the positions of RyR tetramers in tomographic images of dyads of mammalian cardiomyocytes.
* The images, which  are in the TIFF format, consist of sequential parallel planes, betwen 0.5
* and 1 nm apart, through the plane of the dyad * and are generated by the Amira program (Thermo
* Fisher Scientific - FEI). The tetramer is represented by an open rectangle with a default width
* of 27 nm (which can be changed).
*
* Tetramers are placed manually and can then be classified according to the scheme in
* Asghari et. al. Circ. Res. 115(2) 252-66, 2014. The program also calculates nearest
* neighbour distances as well as an alpha shape and a convex hull around the dyad, allowing
* and estimate of the area and the density (tetramers per unit area).
*
* Copyright David Scriven, 2012-2019.
*
* Moore Laboratory, Life Sciences Institute,  2350 Health Sciences
* Mall, University of British Columbia, Vancouver, Canada, V6T 1Z3
*
* This file, ryr_fit3_main.cpp is part of the RYR_FIT program
*
* RYR_FIT links to the proprietary Qt system (ver 4.7) as well as the the free
* CGAL algorithmic library and the free TIFF library.
*
* This program is free software: you can redistribute it and/or modify
* it under the terms of the GNU General Public License as published by
* the Free Software Foundation, either version 3 of the License, or
* any later version.
*
* This program is distributed in the hope that it will be useful,
* but WITHOUT ANY WARRANTY; without even the implied warranty of
* MERCHANTABILITY or FITNESS FOR A PARTICULAR PURPOSE.  See the
* GNU General Public License for more details.
*
* You should have received a copy of the GNU General Public License
* along with this program.  If not, see <https://www.gnu.org/licenses/>.
*****************************************************************************************************/

#include <QApplication>
#include <QGLFormat>
#include "ryr_fit3a.h"
#include <unistd.h>
#include <iostream>
#include <cstdio>

void showoptions(void);

int main(int argc, char *argv[])
{
    if(argc < 2)
    {
        showoptions();
        return 99;
    }

    int status = fork();
    switch (status)
    {
      case -1:
      {
        std::cerr << "\nError - could not fork process\n\n";
        exit(1);
      }
      case 0: // child process
        break;
      default: /// parent process
        exit(0);
    }
    status = setsid();
```

```
    if (status == -1)
    {
      std::cerr << "\nError - could not get valid sid\n\n";
      exit(1);
    }



    QApplication app(argc, argv);
    if(!QGLFormat::hasOpenGL())
    {
        qFatal("This system has no OpenGL support");
        exit(99);
    }

    QGLFormat fmt;
    fmt.setDepth(false);
    QGLFormat::setDefaultFormat(fmt);


    RyRFit* images = new RyRFit;
    if(!images->ParseCmdString(argc, argv))exit(99);
    images->ShowImage();
    return app.exec();
}
void showoptions(void)
{
   using namespace std;


   cerr << "\nUsage: ryr_fit [options] -I image[1...imagen]\n\n";
   cerr << "  where:\n\n";
   cerr << "  image is one or more 2D or 3D TIFF file names. Images can be a single image, multiple\n";
   cerr << "  images or an image stack. Images can be 8 or 16 bit monochrome, RGB or mapped TIFFs\n\n";
   cerr << " If a single monochrome is displayed, the default colour is white although this can be changed.\n";
   cerr << " Example:\n\n";
   cerr << "    ryr_fit -scale 1.2 -I cav3_ryr_c002Z*.tif\n";
   cerr << " will display the sequential TIFFs at scale of 1.2 nm per pixel\n\n";
   cerr << " Options:\n\n";
   cerr << "   -R        Analyse data without bringing up images\n";
   cerr << "   -scale    pixel size (in nm)\n";
   cerr << "   -W n      change tetramer width in nm (default 27)\n\n";
   cerr << " Display options :\n\n";
   cerr << "   -M n     Magnification (default=1)\n";
   cerr << "   Left Mouse button - press then left/right movement moves through individual planes from the image\n";
   cerr << "                       stacks. Left and right arrows on the keyboard will do the same.\n";
   cerr << "   Right Mouse button - pops up menu\n\n";
   cerr << "The program saves two files: tetramer2.dat which contains the tetramer poistions, orientations and\n";
   cerr << "and classifications and nnd2.dat which contains the nearest neighbour distance for each tetramer\n";
   cerr << "The tetramer2.dat file is created on first use, saved and read in on subsequent use of the same data\n\n";
   cerr << endl;
}
```

**** ryr_fit3a.cpp ****

```
/*********************************************************************************************
* ryr_fit is a program designed to place boxes with the correct dimensions and orientation
* at the positions of RyR tetramers in tomographic images of dyads of mammalian cardiomyocytes.
* The images, which  are in the TIFF format, consist of sequential parallel planes, betwen 0.5
* and 1 nm apart, through the plane of the dyad * and are generated by the Amira program (Thermo
* Fisher Scientific - FEI). The tetramer is represented by an open rectangle with a default width
* of 27 nm (which can be changed).
*
* Tetramers are placed manually and can then be classified according to the scheme in
* Asghari et. al. Circ. Res. 115(2) 252-66, 2014. The program also calculates nearest
* neighbour distances as well as an alpha shape and a convex hull around the dyad, allowing
* and estimate of the area and the density (tetramers per unit area).
* Tetramers are placed manually and can then be classified according to the scheme in
* Asghari et. al. Circ. Res. 115(2) 252-66, 2014. The program also calculates nearest
* neighbour distances as well as an alpha shape and a convex hull around the dyad, allowing
* and estimate of the area and the density (tetramers per unit area).
*
* Copyright David Scriven, 2012-2019.
*
* Moore Laboratory, Life Sciences Institute,  2350 Health Sciences
* Mall, University of British Columbia, Vancouver, Canada, V6T 1Z3
*
* This file, ryr_fit3a.cpp is part of the RYR_FIT program
*
* RYR_FIT links to the proprietary Qt system (ver 4.7) as well as the the free
* CGAL algorithmic library and the free TIFF library.
*
* This program is free software: you can redistribute it and/or modify
* it under the terms of the GNU General Public License as published by
* the Free Software Foundation, either version 3 of the License, or
* any later version.
*
* This program is distributed in the hope that it will be useful,
* but WITHOUT ANY WARRANTY; without even the implied warranty of
* MERCHANTABILITY or FITNESS FOR A PARTICULAR PURPOSE.  See the
* GNU General Public License for more details.
*
* You should have received a copy of the GNU General Public License
* along with this program.  If not, see <https://www.gnu.org/licenses/>.
*********************************************************************************************/


#include <QtGui>
#include <QtOpenGL>
#include <QTextStream>
#include <QRegExp>
#include <QStringList>
#include <iostream>
#include <iomanip>
#include <istream>
#include "ryr_fit3a.h"
#include "scale.h"

#include "tiff.h"
#include "newscroll.h"
#include "placement.h"
#include <cstdlib>
#include <cmath>
#include <iomanip>

#include <CGAL/algorithm.h>
#include <CGAL/Delaunay_triangulation_2.h>
#include <CGAL/Alpha_shape_2.h>
#include <CGAL/Polygon_2.h>
#include <CGAL/convex_hull_2.h>

#include <QVector>
```

```
typedef G::FT FT;
typedef CGAL::Polygon_2<G> Polygon_2;
typedef CGAL::Alpha_shape_vertex_base_2<G> Vb;
typedef CGAL::Alpha_shape_face_base_2<G>  Fb;
typedef CGAL::Triangulation_data_structure_2<Vb,Fb> Tds;
typedef CGAL::Delaunay_triangulation_2<G,Tds> Triangulation_2;
typedef CGAL::Alpha_shape_2<Triangulation_2>  Alpha_shape_2;
typedef Alpha_shape_2::Alpha_shape_edges_iterator Alpha_shape_edges_iterator;

using namespace std;

bool RyRFit::bAutoScale;
int  RyRFit::nSmin[3];
int  RyRFit::nSmax[3];

RyRFit::RyRFit()
{
    imageMenu = new QMenu(this);

    planeimage = 0;
    hilite = 0;
    bBitmap = false;
    nNoChannels = 0;
    nNoValidChannels = 0;
    nPerPnts = 0;

    ScaleVal = 0;          // various popup windows

    nZoom = 1;

    cMax = 65535;
    cMin = 0;

    bAutoScale = true;     // autoscale EM image (default)
    bShowZ = true;         // Show PlaneNo
    bScaleBar = false;     // Show a scalebar?
    bSingleColor = false;
    bFlood = false;
    bPartialFlood = false;
    bShowTetramers = true;
    bShowShadow = false;
    bFlip = false;
    bAnalyseOnly = false; // If true (-R option) - no image is shown and we just print out the results

    for(int j= 0; j < 3; j++)
    {
        bScale[j] = false;        // Scale image into fixed range?
        psDst[j] = psD[j] = 0;    // initalize pointers to data
        nSZdim[j] = 0;            // no of Z planes per channel
    }
    bSave = false;  // Flag to save changed file

    bLinePlot = false;
    bDisplayNND = false;
    bLookAhead = false;
    bHideEMImage = false;
    bSingleColor = false;
    bDrawing = false;
    nLookAhead = 1;

    bLeftButtonDown=false;   //mousebutton status
    bRightButtonDown=false;
    bMiddleButtonDown=false;
    bAltPressed = false;

    fontcolour[0] = "red";     //font colours for intensity window
    fontcolour[1] = "green";
```

```
    fontcolour[2] = "blue";

    iminfo.nNoValidChannels = 0;
    iminfo.nXPixelSize = 0;
    iminfo.nYPixelSize = 0;
    iminfo.nZSpacing = 0;

    for(int i = 0; i < 3; i++)
    {
        iminfo.DateandTime[i] = "";
        iminfo.Description[i] = "";
        iminfo.Fmax[i] = 0;
        iminfo.Fmin[i] = 0;
    }

    nNoTetramers = 0;
    nAddedTetramers = 0;
    degtorad=4.0*atan(1.0)/180.0;    // some values we need
    tetramersize=27;
    sqrt2 = sqrt(2.0);
    fortyfivedeg = 45.0 * degtorad;
    totalConvexArea=0.0;
    totalAlphaArea=0.0;
    cSumtype = 'm';
    bEnteredScale = false;
    bAddTetramer = false;           // flags to allow
    bPlacedTetramer = false;
    bModifyTetramer = false;
    bClassifyTetramer = false;
    bShowArea = false;
    bFloodCalculated = false;
    bShowShadow = false;
    nBeingModified = -1;
    nBeingClassified = -1;
    nPlacementType = 1;
    DataFileName="";
    NNFileName ="";
    fnmPerPixel = 0.0;
    ShadowShape = SQUARE;

    fXPixelSize = 0.0;
    fYPixelSize = 0.0;

    nInitialColour = MONO;        //colour for a single, non RGB channel

    linecolor[0]=Qt::red;         // colours for tetramer classifications
    linecolor[1]=Qt::green;
    linecolor[2]=Qt::red;
    linecolor[3]=Qt::blue;
    linecolor[4]=Qt::cyan;
    linecolor[5]=Qt::magenta;
    linecolor[6]=Qt::yellow;
    linecolor[7]=Qt::gray;
}
RyRFit::~RyRFit()
{
    delete imageMenu;
    delete [] hilite;

    delete [] psD[0];
    delete [] psD[1];
    delete [] psD[2];

    delete ScaleVal;
}
void RyRFit::resizeEvent(QResizeEvent *e)
{
    int xp = imageScroll->x();
    int yp = imageScroll->y();
```

```
    int width = imageScroll->frameGeometry().width();


    ScaleVal->moveImage(xp, yp, width);
    QWidget::resizeEvent(e);
    updateGL();
}
void RyRFit::moveEvent(QMoveEvent *e)
{
    int xp = imageScroll->x();
    int yp = imageScroll->y();
    int width = imageScroll->frameGeometry().width();


    ScaleVal->moveImage(xp, yp, width);
    QWidget::moveEvent(e);
}
void RyRFit::contextMenuEvent(QContextMenuEvent *e)
{
    QMenu* menu;


    menu=imageMenu;


    menu->exec(e->globalPos());
}
void RyRFit::keyPressEvent( QKeyEvent *e )
{
    bool bAlterTetramer = bModifyTetramer || bAddTetramer;
    switch( e->key() )
    {
    case Qt::Key_Escape:
        if(bDrawing)
        {
            bDrawing = false;
            nPerPnts = 0;
            updateGL();
        }
        else
          close();

        break;
    case Qt::Key_Right:
        if(bDrawing)return;
        if(bAlterTetramer)
        {
            TetramerPosn.rx()++;
            updateGL();
        }
        else
            imageNextPlane();
        break;
    case Qt::Key_Left:
        if(bDrawing)return;
        if(bAlterTetramer)
        {
            TetramerPosn.rx()--;
            updateGL();
        }
        else
            imagePrevPlane();
        break;
    case Qt::Key_Up:
        if(bAlterTetramer)
        {
            TetramerPosn.ry()++;
            updateGL();
        }
        break;
    case Qt::Key_Down:
        if(bAlterTetramer)
        {
            TetramerPosn.ry()--;
            updateGL();
```

```
        }
        break;
    case Qt::Key_Home:
        imageFirstPlane();
        break;
    case Qt::Key_End:
        imageLastPlane();
        break;
    case Qt::Key_Delete:
    case Qt::Key_Backspace:
        if(!bDrawing)return;
        if(nPerPnts > 0)
        {
            nPerPnts--;
            previous = PerPnt[max(0,nPerPnts-1)];
            updateGL();
        }
        break;
    case Qt::Key_A:
        if(bModifyTetramer ||bClassifyTetramer)
            return;
        addTetramer();
        break;
    case Qt::Key_B:
        if(bAlterTetramer)
            return;
        if(bClassifyTetramer)
           tetramerClassification('b');
        break;
    case Qt::Key_C:
        if(bAlterTetramer)
            return;
        if(bClassifyTetramer)
           tetramerClassification('c');
        else
           classifyTetramer();
        break;
    case Qt::Key_D:
        if(bAlterTetramer)
           deleteTetramer();
        break;
    case Qt::Key_E:
        if(bClassifyTetramer)
        {
            bClassifyTetramer = false;
            nBeingClassified = -1;
            updateGL();
        }
        break;
    case Qt::Key_H:
        hideEMImage();
        break;
    case Qt::Key_I:
        if(bAlterTetramer)
            return;
        if(bClassifyTetramer)
           tetramerClassification('i');
        break;
    case Qt::Key_K:
        changeLookAhead();
        break;
    case Qt::Key_L:
        bLookAhead = !bLookAhead;
        if(bLookAhead)
            nLookAhead = 4;
        else
            nLookAhead = 1;
        CalculateBitmap();
        break;
    case Qt::Key_M:
        if(bAlterTetramer)
            return;
        modifyTetramer();
```

```
    break;
case Qt::Key_N:
    showNearestNeighbours();
    break;
case Qt::Key_P: // Measure Line length
    imageLine();
    break;
case Qt::Key_Q:
    HideZ();
    break;
case Qt::Key_R:
    showExtent();
    break;
case Qt::Key_S:
    if(bAlterTetramer)
        saveTetramer();
    if(bClassifyTetramer)
       tetramerClassification('s');
    break;
case Qt::Key_T:
    showPlacement();
    break;
case Qt::Key_U:
    if(bAlterTetramer)
        return;
    if(bClassifyTetramer)
       tetramerClassification('u');
    break;
case Qt::Key_W:
    ChangeTetramerWidth();
    updateGL();
    break;
case Qt::Key_F1:
    showRedTetramers();
    break;
case Qt::Key_F2:
    hideTetramers();
    break;
case Qt::Key_F3:
    changeShadowOutline();
    break;
case Qt::Key_F4:
    toggleShadow();
    break;
case Qt::Key_F5:
    DrawContour();
    break;
case Qt::Key_F6:
    getScaleBarSize();
    break;
case Qt::Key_F7:
    setFloodFillType();
    break;
case Qt::Key_F9:
    ComputeFloodfill();
    break;
case Qt::Key_F10:
    Instructions();
    break;
case Qt::Key_F11:
    ChangeTetramerWidth();
    break;
case Qt::Key_F12:
    FlipImage();
    break;
case Qt::Key_1:
    LookAhead(1);
    break;
case Qt::Key_2:
    LookAhead(2);
    break;
case Qt::Key_3:
    LookAhead(3);
```

```
        break;
    case Qt::Key_4:
        LookAhead(4);
        break;
    case Qt::Key_5:
        LookAhead(5);
        break;
    case Qt::Key_6:
        LookAhead(6);
        break;
    case Qt::Key_7:
        LookAhead(7);
        break;
    case Qt::Key_8:
        LookAhead(8);
        break;
    case Qt::Key_9:
        LookAhead(9);
        break;
    case Qt::Key_Plus:
        imageZoomIn();
        break;
    case Qt::Key_Minus:
        imageZoomOut();
        break;
    case Qt::Key_Alt:
    case Qt::Key_AltGr:
        bAltPressed = true;
        break;
    case Qt::Key_Control:
        if(bAutoScale)
        {
            FixedScale();
            autoAct->setChecked(true);
        }
        else
        {
            AutoScale();
            fixedAct->setChecked(true);
        }
        break;
    default:
        break;
    }
}
void RyRFit::keyReleaseEvent( QKeyEvent *e )
{
    if(e->key() == Qt::Key_Alt || e->key() == Qt::Key_AltGr)
        bAltPressed = false;
}
void RyRFit::wheelEvent(QWheelEvent *event)
{
    if(bAddTetramer || bModifyTetramer)
    {
        TetramerAngle += int(event->delta()/40);
        TetramerAngle %= 90;
        updateGL();
    }
}
void RyRFit::mousePressEvent( QMouseEvent *e )
{
    mousePos = LimitMouse(e->x(),e->y());
    switch (e->button())
    {
    case Qt::LeftButton:
        bLeftButtonDown=true;
        StartPt = EndPt = mousePos;
        if(bModifyTetramer || bClassifyTetramer)
        {
            findTetramer(mousePos);
        }
        if(bAddTetramer)
        {
```

```
            if(bPlacedTetramer)
            {
                findTetramer(mousePos);
            }
            else
            {
                int mxPos = mousePos.x()/nZoom;
                int myPos = mousePos.y()/nZoom;
                TetramerPosn = QPoint(mxPos, myPos);
                TetramerAngle = 0;
                bPlacedTetramer = true;
                updateGL();
                return;
            }
        }
        break;
    case Qt::MidButton:
        bMiddleButtonDown=true;
        StartPt=mousePos;
        if(bDrawing)
        {
            if(nPerPnts == 0)
            {
                PerPnt[0] = RealPos(mousePos);
                previous = PerPnt[0];
                nPerPnts++;
            }
            else
            {
                // move mouse to last point if drawing
                previous = PerPnt[nPerPnts];
                QCursor::setPos(lastMousePosn);
            }
            updateGL();
        }
        break;
    case Qt::RightButton:
        break;
    default:
        QWidget::mousePressEvent(e);
        break;
    }
}
void RyRFit::mouseReleaseEvent( QMouseEvent *e )
{
    EndPt = LimitMouse(e->x(), e->y());
    currentPos=EndPt;
    switch (e->button())
    {
    case Qt::LeftButton:
        bLeftButtonDown=false;
        if(bLinePlot)
        {
            if(StartPt != EndPt)
            {
                glBegin(GL_LINES);
                glVertex2i(StartPt.x(), StartPt.y());
                glVertex2i(EndPt.x(), EndPt.y());
                glEnd();
                updateGL();
            }
        }


        break;
    case Qt::RightButton:
        QWidget::mouseReleaseEvent(e);
        break;


    case Qt::MidButton:
        bMiddleButtonDown=false;
        if(bDrawing)
        {
```

```
            lastMousePosn = QCursor::pos();
            if(nPerPnts > 0)
            {
              FloodAct->setEnabled(true);
              FloodAct->setText("Isolate Region {F9}");
            }
        }
        setCursor(Qt::ArrowCursor);
        releaseKeyboard();
        break;


    default:
        QWidget::mouseReleaseEvent(e);
        break;
    }
}
void RyRFit::mouseMoveEvent( QMouseEvent *e )
{
    QPoint newMousePos;


    if(bLeftButtonDown && !(bLinePlot))
        newMousePos= QPoint(e->x(), windowY-e->y());
    else
        newMousePos = LimitMouse(e->x(),e->y());


    currentPos = newMousePos;


    if(bLeftButtonDown)
    {
        if(bLinePlot)
        {
            EndPt = newMousePos;
            updateGL();
        }
        else
        {
            int nXmove = newMousePos.x() - mousePos.x();


            if(nXmove > 0)
                imageNextPlane();


            if(nXmove < 0)
                imagePrevPlane();


            mousePos = newMousePos;
        }
    }
    if(bMiddleButtonDown && bDrawing)
    {
        // Limit the number of perimeter points
        if(nPerPnts == ( TPERPNTS - 1 ))
        {
          cerr << "Cannot store anymore points!" << endl;
          return;
        }
        QPoint current = RealPos(newMousePos);
        if(current.x() != previous.x() || current.y() != previous.y())
        {
          previous = current;
          PerPnt[nPerPnts++] = current;
          updateGL();
        }
    }
}
void RyRFit::LookAhead(int n)
{
    if(bLookAhead)
    {
        nLookAhead = n;
        CalculateBitmap();
```

```
    }
}
void RyRFit::changeLookAhead()
{
    if(bLookAhead)
    {
        if(cSumtype == 'm')
            cSumtype = 's';
        else
            cSumtype = 'm';
        CalculateBitmap();
    }
}
QPoint RyRFit::LimitMouse(int x, int y)
{
    // keep mouse values within image
    // Transform y coordinate
    y = windowY - y;
    if(x < 0) x = 0;
    else if(x > nImageXdim-1)x = nImageXdim-1;
    if(y < 0)y = 0;
    else if(y > nImageYdim-1)y = nImageYdim-1;


    return QPoint(x, y);
}
int RyRFit::ShowImage()
{
    lookup = new GLubyte* [3];
    sPmin = new int* [3];
    sPmax = new int* [3];
    nPMaxpos = new int* [3];
    nPMinpos = new int* [3];
    nPpts = new int* [3];
    float existingscale = 0;


    if(!bAnalyseOnly)
    {
        // Check stacks for dimensions and type


        if(!CheckDimAndType(1))return 99;
        if(nNoStacks > 1)
        {
            if(nSInputType[0] != TIFF_Mono_8bit && nSInputType[0] != TIFF_Mono_16bit)
            {
                cerr << " Cannot have multiple stacks with RGB & Mapped TIFFs\n" << endl;
                return 50;
            }
            if(!CheckDimAndType(2))return 99;
            if(nSInputType[1] != TIFF_Mono_8bit && nSInputType[1] != TIFF_Mono_16bit)
            {
                cerr << " Cannot have multiple stacks with RGB & Mapped TIFFs\n" << endl;
                return 50;
            }
            if(  (nSXdim[0] != nSXdim[1])
                 || (nSYdim[0] != nSYdim[1])
                 || (nSZdim[0] != nSZdim[1]))
            {
                cerr << " Dimensions of images in red and green channels do not match" << endl;
                return 50;
            }
        }
        if(nNoStacks == 3)
        {
            if(!CheckDimAndType(3))return 99;
            if(nSInputType[2] != TIFF_Mono_8bit && nSInputType[2] != TIFF_Mono_16bit)
            {
                cerr << " Cannot have multiple stacks with RGB & Mapped TIFFs\n" << endl;
                return 50;
            }
            if(  (nSXdim[0] != nSXdim[2])
                 || (nSYdim[0] != nSYdim[2])
                 || (nSZdim[0] != nSZdim[2]))
```

```
    {
        cerr << " Dimensions of images in red and blue channels do not match" << endl;
        return 50;
    }
}

nXdim = nSXdim[0];
nYdim = nSYdim[0];
nZPlanes = nSZdim[0];

nPixelsPerPlane = nXdim*nYdim;
int nPixelsPerChannel = nPixelsPerPlane*nZPlanes;

nNoChannels = nNoStacks;

if(nSInputType[0] != TIFF_Mono_8bit && nSInputType[0] != TIFF_Mono_16bit)
{
    nNoChannels = 3;
    bRGB = true;
}
else
    bRGB=false;

for(int n=0; n < nNoChannels; n++)
{
    psD[n] = new uint16[nPixelsPerChannel];
    psDst[n] = psD[n];
}

// Read in tiff files

for(int l = 0; l < nNoStacks; l++)
    if(!ReadTiffStack(l))return 99;

CheckChannels();
cerr << "\n";

nZmin = 1;
nZmax = nZPlanes;
nPlaneNo=nZmin;

QDesktopWidget *desktop = QApplication::desktop();
QRect ScreenSize = desktop->availableGeometry();
nXScreenMax = ScreenSize.width();
nYScreenMax = ScreenSize.height();

createActions();
createMenus();
FloodAct->setEnabled(false);
toggleShadowAct->setEnabled(false);
changeShadowAct->setEnabled(false);

nPerPnts = 0;
hilite = new bool [nXdim*nYdim];

// resize window to size of image or zoom if necessary
// setup window parameters

if(nZoom > 1 || (nXdim < 65 || nYdim < 50))
{
    nZoom = max(nZoom,int(100/min(nXdim,nYdim)));
    nImageXdim = nZoom*nXdim;
    nImageYdim = nZoom*nYdim;
}
else
{
```

```
        nImageXdim = nXdim;
        nImageYdim = nYdim;
    }
    nBitmapXdim = nXdim;
    nBitmapYdim = nYdim;

    iminfo.nXdim = nXdim;
    iminfo.nYdim = nYdim;
    iminfo.nZdim = nZPlanes;
    iminfo.nNoValidChannels = nNoValidChannels;

    for(int i = 0; i < nNoValidChannels; i++)
    {
        iminfo.Fmin[i] = nFmin[i];
        iminfo.Fmax[i] = nFmax[i];
        iminfo.nValidChannel[i] = nValidChannel[i];
    }
    iminfo.bRGB = bRGB;

    //  Parameters for image window

    imageScroll = new NewScroll;

    imageScroll->setWidget(this);
    imageScroll->setBackgroundRole(QPalette::Dark);

    setMinimumSize(nImageXdim,nImageYdim);
    resize(nImageXdim,nImageYdim);
    imageScroll->resize(nImageXdim+6,nImageYdim+6);
    windowY = nImageYdim;

    imageScroll->setWindowIcon(QIcon(":/dim.png"));

    sysfont = new QFont("System");
    sysfont->setPixelSize(15);

    if(nNoChannels == 1)
        CreateColourMap(nInitialColour);
}
// Check for Data File
QString fpath;
QFileInfo fi(FileName[0].at(0));
fi.makeAbsolute();
fpath = fi.path();

DataFileName=fpath + QString("/tetramer2.dat");

bool bExistingScale =false;
QString FileName = DataFileName;

QFileInfo dfi(FileName);
if(dfi.exists())
{
    QFile file(FileName);
    if(!file.open(QIODevice::ReadOnly | QIODevice::Text))
        QMessageBox::information(0,"error",file.errorString());

    QTextStream input(&file);
    int xp, yp;
    nNoTetramers = 0;
    forever
    {
        QString tline=input.readLine();
        if (tline.isEmpty())
            break;
```

```
//          cout << "Line #" << (nNoTetramers+1) << ' ' << qPrintable(tline) << ' ';

            if(tline.contains("scale="))
            {
                existingscale=(tline.remove("scale=")).toFloat();
                bExistingScale = true;
            }
            else
            {
                QStringList tl=tline.split(QRegExp("\\s+"), QString::SkipEmptyParts);
//              cerr << "size = " << tl.size() << "\n";
                xp = tl.at(0).toInt();
                yp = tl.at(1).toInt();
                if(xp == 0 || yp == 0)
                {
                    cerr << "Invalid tetramer values in input " <<  qPrintable(tline) << " - ignored\n";
                    continue;
                }
                tm[nNoTetramers].centre = QPoint(xp, yp);
                tm[nNoTetramers].angle = tl.at(2).toInt();
                if(tl.size() == 4)
                    tm[nNoTetramers].classification = tl.at(3).at(0).toAscii();
                else
                    tm[nNoTetramers].classification = 'u';
//              cout << "Output: " << xp << ' ' << yp << ' ' << tm[nNoTetramers].angle << ' ' << tm[nNoTetramers].classification << "\n";
                nNoTetramers++;
            }
        }
        file.close();
        if(nNoTetramers == 0)
            cerr << "No valid tetramers in input\n";
    }
    else
    {
        QString Desc = iminfo.Description[0];
        if(Desc.contains("Pixel size = "))
        {
            int p = Desc.indexOf("Pixel size = ") + 12;
            int q = Desc.indexOf(" nm");
            fnmPerPixel = (Desc.mid(p, q-p + 1)).toDouble();
            existingscale = fnmPerPixel;
            bExistingScale = true;
            bEnteredScale = true;
        }

        if(bAnalyseOnly)
        {
          std::cerr << "Error - Can't find tetramer2.dat file\n\n";
          return 50;
        }
    }

    if(bEnteredScale)
    {
        if(bExistingScale && (fnmPerPixel != existingscale))
        {
            QString label = QString("Scaling with file is %1 while entered scale is %2\nPlease enter the correct scale
(nm/pixel):").arg(existingscale).arg(fnmPerPixel);
            fnmPerPixel = QInputDialog::getDouble(this,"Scaling mismatch", label, existingscale, 0.0, 3.0);
        }
    }
    else
    {
        if(bExistingScale)
           fnmPerPixel = existingscale;
        else
           fnmPerPixel = QInputDialog::getDouble(this, "Missing Scale", "No scale supplied either externally or within the file\nPlease enter the correct scale
(nm/pixel):", existingscale, 0.0, 3.0);
    }

    NNFileName=fpath + QString("/nnd2.dat");
```

```
    if(bAnalyseOnly)
    {
        fnmPerPixel = existingscale;
        tetramerwidth = tetramersize/fnmPerPixel;
        tetramerdiag = tetramerwidth/sqrt2;
        CloseProgram();
    }
    else
    {
        setupSingleTetramer();

        CreateStack();

        updateGL();

        // define ancillary windows

        ScaleVal = new ScaleWnd(this, iminfo, nFmin, nFmax, redlut, greenlut, bluelut);
        TetramerPlacement = new Placement;

        connect(TetramerPlacement, SIGNAL(Checkerboard(double)), this, SLOT(setupCheckerboard(double)));
        connect(TetramerPlacement, SIGNAL(SingleTetramer()), this, SLOT(setupSingleTetramer()));
        connect(TetramerPlacement, SIGNAL(LaiCheckerboard()), this, SLOT(setupLaiChckrbrd()));
        connect(TetramerPlacement, SIGNAL(LaiSidebySide()), this, SLOT(setupLaiSidebySide()));
        // position  & show the image

        int xipos = (nXScreenMax - nImageXdim)/2; // + (rand() % 50);
        int yipos = (nYScreenMax - nImageYdim)/2; // + (rand() % 25);

        imageScroll->move(xipos, yipos);
        imageScroll->show();
    }

    return 0;
}
/**************************************************************
        Colour maps & color handling
**************************************************************/
void RyRFit::CreateColourMap(int nColourDisplay)
{

// colour map for Monochrome images
    int red = 0;
    int green = 0;
    int blue = 0;
    int i;

    int nInitVal = 0;
    int nSign = 1;
    int nColourDelta = 1;

    switch (nColourDisplay)
    {
    case RED:
        red = nColourDelta;
        break;
    case YELLOW:
        red = nColourDelta;
        green = nColourDelta;
        break;
    case GREEN:
        green = nColourDelta;
        break;
    case CYAN:
        green = nColourDelta;
        blue = nColourDelta;
        break;
    case BLUE:
```

```
        blue = nColourDelta;
        break;
    case MAGENTA:
        red = nColourDelta;
        blue = nColourDelta;
        break;
    case MONO:
        red = nColourDelta;
        green = nColourDelta;
        blue = nColourDelta;
        break;
    case INVMONO:
        nInitVal = 255;
        nSign = -1;
        red = nColourDelta;
        green = nColourDelta;
        blue = nColourDelta;
        break;
    }

    if(nColourDisplay < LUT)
    {
        for (i = 0; i < 256; i++)
        {
            int j = nInitVal + nSign*i;
            redlut[i] = j*red;
            greenlut[i] = j*green;
            bluelut[i] = j*blue;
        }
     }
    else if(nColourDisplay == LUT)
    {
        QString name = QFileDialog::getOpenFileName( this,
                                            "Select a LUT file", "/usr/dim/lib", "(*.lut)");
        if (name.isEmpty() || name.isNull())return;

        ifstream lut(qPrintable(name));
        char szString[80];
        lut.getline(szString,60);


        int ired, igreen, iblue;
        for(i=0; i < 256; i+=2)
        {
            lut.getline(szString,60);
            ired = igreen = iblue = 0;
            sscanf(szString,"%d %d %d",&ired, &igreen, &iblue);
            redlut[i] = redlut[i+1] = ired;
            greenlut[i] = greenlut[i+1]= igreen;
            bluelut[i] = bluelut[i+1] = iblue;
        }

    }
    else
    {
        nColourDelta = 1;
        for (i = 0; i < 256; i++)
        {
            redlut[i] = i*nColourDelta;
            if(i < 128)
                greenlut[i] = i*2*nColourDelta;
            else
                greenlut[i] = 255-(i-128)*2*nColourDelta;
            bluelut[i] = 255 - i*nColourDelta;
        }
    }
    if(!bAutoScale && ScaleVal->isVisible())ScaleVal->SetColorMap();
    return;
}
/************************************************************/
void RyRFit::initializeGL()
{
    glDisable(GL_DITHER);
    glDisable(GL_FOG);
```

```
    glDisable(GL_LIGHTING);
    glDisable(GL_LINE_STIPPLE);
    glDisable(GL_STENCIL_TEST);
    glDisable(GL_TEXTURE_1D);
    glDisable(GL_TEXTURE_2D);
    glShadeModel(GL_FLAT);
    glLineStipple (1, 0x000F);


    // set the packing alignment
    glPixelStorei(GL_UNPACK_ALIGNMENT, 1);
    qglClearColor(Qt::black);
}
void RyRFit::resizeGL(int x, int y)
{
    //    cerr << "resizeGL\n";
    //    cout << " (" << XViewSt<< "," << YViewSt <<") to ("<< XViewEnd << "," << YViewEnd <<")" << endl;
    //    glViewport(0, 0,GLint(x-1), GLint(x-1));
    glMatrixMode(GL_PROJECTION);
    glLoadIdentity();
    glOrtho(0.0f, GLfloat(x), 0.0f, GLfloat(y), -1, 1);
    glViewport(0, 0,GLint(x), GLint(y));
    glMatrixMode(GL_MODELVIEW);
    glLoadIdentity();
    updateGL();
}
void RyRFit::drawTetramer(QPoint pos, int angle, char classification)
{
    double tdiag;
    if(bFlip)
        angle = 180 - angle;
    double angleinradians = angle*degtorad+fortyfivedeg;


    if(classification == 'l')
        tdiag = laicheckerboarddiag;
    else if(classification == 'L')
        tdiag = laisidebysidediag;
    else
        tdiag = tetramerdiag;


    double x1 = (tdiag * cos (angleinradians))*nZoom;
    double y1 = (tdiag * sin (angleinradians))*nZoom;
    double xpos = pos.x()*nZoom;
    double ypos;


    if(bFlip)
        ypos = windowY-nZoom*pos.y();
    else
        ypos = pos.y()*nZoom;


    if(bFlood)
    {
        if(hilite[pos.x()+nXdim*pos.y()])return;
    }
    QPoint p1 = QPoint(qRound(xpos + x1),qRound(ypos + y1)) ;
    QPoint p2 = QPoint(qRound(xpos - y1), qRound(ypos + x1));
    QPoint p3 = QPoint(qRound(xpos - x1), qRound(ypos - y1));
    QPoint p4 = QPoint(qRound(xpos + y1), qRound(ypos - x1));
    glBegin(GL_LINE_LOOP);
     glVertex2i(p1.x(), p1.y());
     glVertex2i(p2.x(), p2.y());
     glVertex2i(p3.x(), p3.y());
     glVertex2i(p4.x(), p4.y());
    glEnd();
    ntm[0].xpos = xpos;
    ntm[0].ypos = ypos;
}
void RyRFit::drawCheckerboard(QPoint pos, int angle)
{
    double pointangleinradians = angle*degtorad+fortyfivedeg;
```

```
    double x1 = (tetramerdiag * cos (pointangleinradians))*nZoom;
    double y1 = (tetramerdiag * sin (pointangleinradians))*nZoom;
    ntm[0].xpos = pos.x()*nZoom;
    ntm[0].ypos = pos.y()*nZoom;
    for(int j = 1; j < 5; j++)
    {
      float angleinradians = baseangleinradians + (angle + (j-1)*90)*degtorad;
      ntm[j].xpos = (pos.x() + centredistance*cos(angleinradians))*nZoom;
      ntm[j].ypos = (pos.y() + centredistance*sin(angleinradians))*nZoom;
    }


    for(int i = 0; i < 5; i++)
    {
      if(ntm[i].bStipple)
          glEnable(GL_LINE_STIPPLE);
      else
          glDisable(GL_LINE_STIPPLE);
      glBegin(GL_LINE_LOOP);
         glVertex2i(qRound(ntm[i].xpos + x1), qRound(ntm[i].ypos + y1));
         glVertex2i(qRound(ntm[i].xpos - y1), qRound(ntm[i].ypos + x1));
         glVertex2i(qRound(ntm[i].xpos - x1), qRound(ntm[i].ypos - y1));
         glVertex2i(qRound(ntm[i].xpos + y1), qRound(ntm[i].ypos - x1));
      glEnd();
    }
    glDisable(GL_LINE_STIPPLE);
}
void RyRFit::drawLaiSidebySide(QPoint pos, int angle)
{
    double pointangleinradians = (angle+5)*degtorad+fortyfivedeg;


    double x1 = (tetramerdiag * cos (pointangleinradians))*nZoom;
    double y1 = (tetramerdiag * sin (pointangleinradians))*nZoom;
    ntm[0].xpos = pos.x()*nZoom;
    ntm[0].ypos = pos.y()*nZoom;


    int k = 0;
    for(int j = 1; j < 9; j+=2)
    {
      double angleinradians = baseangleinradians + (angle + k*90)*degtorad;
      ntm[j].xpos = (pos.x() + centredistance*cos(angleinradians))*nZoom;
      ntm[j].ypos = (pos.y() + centredistance*sin(angleinradians))*nZoom;
      k++;
    }


    k = 0;
    for(int j = 2; j < 9; j+=2)
    {
      double angleinradians = baseangleinradians + fortyfivedeg + (angle + k*90)*degtorad;
      ntm[j].xpos = (pos.x() + centredistance*sqrt2*cos(angleinradians))*nZoom;
      ntm[j].ypos = (pos.y() + centredistance*sqrt2*sin(angleinradians))*nZoom;
      k++;
    }
    for(int i = 0; i < 9; i++)
    {
      if(ntm[i].bStipple)
          glEnable(GL_LINE_STIPPLE);
      else
          glDisable(GL_LINE_STIPPLE);
      glBegin(GL_LINE_LOOP);
         glVertex2i(qRound(ntm[i].xpos + x1), qRound(ntm[i].ypos + y1));
         glVertex2i(qRound(ntm[i].xpos - y1), qRound(ntm[i].ypos + x1));
         glVertex2i(qRound(ntm[i].xpos - x1), qRound(ntm[i].ypos - y1));
         glVertex2i(qRound(ntm[i].xpos + y1), qRound(ntm[i].ypos - x1));
      glEnd();
    }
    glDisable(GL_LINE_STIPPLE);
}
void RyRFit::paintGL()
{
    glClear(GL_COLOR_BUFFER_BIT| GL_DEPTH_BUFFER_BIT);
    if(bFlip)
```

```
{
    glRasterPos2i(0, windowY);
    glPixelZoom(GLfloat(nZoom), GLfloat(-nZoom));
}
else
    glPixelZoom(GLfloat(nZoom), GLfloat(nZoom));

if(!bHideEMImage)
{
    GLubyte* bitmap = planeimage;
    glDrawPixels(nBitmapXdim,nBitmapYdim,GL_RGB,GL_UNSIGNED_BYTE, bitmap);
}
glRasterPos2i(0,0);

if(bLookAhead)
{
    infoLabel.sprintf("L %d",nLookAhead-1);
    if(cSumtype == 'm')
        infoLabel += " MIN";
    else
        infoLabel += " SUM";

    qglColor(Qt::white);
    glRecti(2, windowY-10, 85,windowY-30);
    qglColor(Qt::red);
    renderText(5, 25, infoLabel, *sysfont);
}

infoLabel.sprintf("Tetramer width = %d nm", qRound(tetramersize));
renderText(50, 25,infoLabel, *sysfont);

if(bShowArea)
{
    qglColor(Qt::blue);
    PlotPerimeters();
    qglColor(Qt::red);
    infoLabel.sprintf("%d Tetramers", nNoTetramers);
    renderText(50, 45, infoLabel, *sysfont);
    infoLabel.sprintf("Convex Hull : cover  %.1f%% of the area - density =  %.2e nm-2",percentTetramerAreaConvex, TetramerDensityConvex);
    renderText(50, 65, infoLabel, *sysfont);
    infoLabel.sprintf("Alpha Shape : cover  %.1f%% of the area - density =  %.2e nm-2",percentTetramerAreaAlpha, TetramerDensityAlpha);
    renderText(50, 85, infoLabel, *sysfont);
}

if(bAddTetramer)
{
    qglColor(Qt::white);
    switch(nPlacementType)
    {
    case 1:
        drawTetramer(TetramerPosn, TetramerAngle);
        break;
    case 2:
        drawCheckerboard(TetramerPosn, TetramerAngle);
        break;
    case 3:
        drawLaiSidebySide(TetramerPosn, TetramerAngle);
        break;
    case 4:
        drawCheckerboard(TetramerPosn, TetramerAngle);
        break;
    }
}

if(bModifyTetramer)
{
    qglColor(Qt::blue);
    drawTetramer(TetramerPosn, TetramerAngle);
}
```

```
if(bClassifyTetramer)
{
    qglColor(Qt::white);
    drawTetramer(tm[nBeingClassified].centre, tm[nBeingClassified].angle);
}


if(bShowTetramers)
{
    for(int i = 0; i < nNoTetramers; i++)
    {
        if(i == nBeingModified || i == nBeingClassified)
            continue;
        if(bSingleColor || bFlood)
            qglColor(Qt::red);
        else
        {
            switch(tm[i].classification)
            {
            case 'u':
                qglColor(Qt::red);
                break;
            case 'c':
                qglColor(Qt::green);
                break;
            case 's':
                qglColor(Qt::yellow);
                break;
            case 'i':
                qglColor(Qt::cyan);
                break;
            case 'b':
                qglColor(Qt::magenta);
                break;
            default:
                qglColor(Qt::white);
            }
        }
        drawTetramer(tm[i].centre, tm[i].angle, tm[i].classification);
    }
}


if(bDrawing)
{
    QPoint* prl = PerPnt;
    if(nPerPnts != 0)
    {
        qglColor(Qt::magenta);
        glBegin(GL_LINE_STRIP);
        for (int j = 0; j < nPerPnts; j++)
        {
            glVertex2i(nZoom*(prl->x()), nZoom*(prl->y()));
            prl++;
        }
        glEnd();
    }
}


if(bDisplayNND)
{
    sysfont->setPixelSize(11);
    for(int j=0; j < nNoTetramers; j++)
    {
        int k=qMin(7,int(mnnd[j]-tetramerwidth)/2);
        //          int hue=300.0*(mnnd[j]-nndmin)/(nndmax-nndmin);
        //          QColor lc = QColor::fromHsv(hue, 255, 255);
        QPoint p1 = nnLine[j].p1();
        QPoint p2 = nnLine[j].p2();
        if(bSingleColor)
            qglColor(Qt::green);
        else
            qglColor(linecolor[k]);
```

```
            glBegin(GL_LINES);
            glVertex2f(p1.x()*nZoom, p1.y()*nZoom);
            glVertex2f(p2.x()*nZoom, p2.y()*nZoom);
            glEnd();
            infoLabel.sprintf("%.1f",mnnd[j]);
            int xpos = ((p1.x()+p2.x())/2 - 4)*nZoom -8;
            int ypos = windowY-((p1.y()+p2.y())/2)*nZoom;
            qglColor(Qt::white);
            renderText(xpos, ypos, infoLabel, *sysfont);
        }
    }


    sysfont->setPixelSize(15);
    qglColor(Qt::magenta);
    if(bScaleBar)
    {
        qglColor(Qt::white);
        glRecti(5,windowY-5*nZoom,5+int(nScaleBarLength/fnmPerPixel)*nZoom, 10*nZoom);
    }


    if(bShowZ)
    {
        glPixelZoom(1.0f,1.0f);
        qglColor(Qt::red);
        infoLabel.sprintf("%d",nPlaneNo);
        renderText(nImageXdim-45, windowY-5*nZoom, infoLabel, *sysfont);
    }
}
void RyRFit::imageLine()
{
    bLinePlot = !bLinePlot;
    if(bLinePlot)
        StartPt = EndPt = QPoint(0,0);


    updateGL();
}
void RyRFit::LineDraw()
{
    ostringstream lv, lv2;
    if(EndPt == StartPt)return;
    QPoint EndL = RealPos(EndPt);
    QPoint StartL = RealPos(StartPt);
    double dXdist= double(StartL.x() - EndL.x());
    double dYdist= double(StartL.y() - EndL.y());
    bool bSizedPix = false;
    if(fXPixelSize > 0.0 && fYPixelSize > 0.0)
    {
        bSizedPix = true;
        dXdist *= fXPixelSize/1000.0;
        dYdist *= fYPixelSize/1000.0;
    }
    double dDist = sqrt(dXdist*dXdist + dYdist*dYdist);


    qglColor(Qt::green);
    glBegin(GL_LINES);
    glVertex2i(StartPt.x(), StartPt.y());
    glVertex2i(EndPt.x(), EndPt.y());
    glEnd();
    lv << "From: " << (StartL.x()+1) << " " << (StartL.y()+1) << " to: ";
    lv << (EndL.x()+1) << " " << (EndL.y()+1) << ends;
    QString LineVal = (lv.str()).c_str();
    glPixelZoom(1.0f, 1.0f);


    renderText(5, 12, LineVal, *sysfont);
    char mu = 0xb5;
    if(bSizedPix)
        lv2 << "dist: "  << setprecision(4) << dDist << ' ' << mu << 'm' << ends;
    else
        lv2 << "dist: "  << setprecision(3) << dDist << ends;
```

```
    LineVal = (lv2.str()).c_str();
    renderText(5, 26, LineVal, *sysfont);
    glPixelZoom(GLfloat(nZoom), GLfloat(nZoom));
}
void RyRFit::CreateStack()
{
    if(planeimage == 0)
    {
        planeimage = new GLubyte[3*nXdim*nYdim];
        if(!planeimage)
        {
            cerr << "Unable to allocate space for plane image - Aborting!\n" << endl;
            CloseProgram();
        }
        CalculateBitmap();
    }
}
void RyRFit::CalculateBitmap()
{
    uint16 *pImage[3];
    pImage[0] = 0;
    pImage[1] = 0;
    pImage[2] = 0;
    uint16 *pID[3];


    bool bSingleImage = (nNoValidChannels == 1 && !bRGB);


    GLubyte min_color = 0;
    GLubyte max_color;
    if(bSingleImage)
        max_color = 248;
    else
        max_color = 255;


    memset(planeimage, 0, nXdim*nYdim*3);


    for(int m = 0; m < nLookAhead; m++)
    {
        GLubyte *pBData = planeimage;


        int nPlane = nPlaneNo + m;
        if(nPlane > nZPlanes)continue;


        for(int i=0 ; i < nNoValidChannels; i++)
        {
            int j = nValidChannel[i];
            pImage[j] = psD[j] + (nPlane-1)*nPixelsPerPlane;
        }


        int maxR;
        int minR;
        int minval[3];
        int nRange[3];
        int kstart[3];
        int kstop[3];
        for(int i = 0; i < nNoValidChannels; i++)
        {
            int j = nValidChannel[i];
            if(!bScale[j])
            {
                minR = minval[j] = sPmin[j][nPlane-1];
                maxR = sPmax[j][nPlane-1];
                kstart[j] = 0;
                kstop[j] = maxR - minval[j] + 1;
            }
            else
            {
                minval[j] = nFmin[j];
                minR = nSmin[j];
                kstart[j] = minR - minval[j];
```

```
        for(int m = 0; m < kstart[j]; m++)
            lookup[j][m] = min_color;   // set colors below min

        maxR = nSmax[j];
        kstop[j] = maxR - minval[j];
        int kmid = nFmax[j]-minval[j]+1;
        for(int m = kstop[j]; m < kmid; m++)
            lookup[j][m] = max_color;   // set colors above max
    }

    float fCdel;
    nRange[i] = maxR - minR;
    if(nRange[i] > 0)
        fCdel = float(max_color - min_color)/float(nRange[i]);
    else
        fCdel = 0;

    for(int k = kstart[j]; k < kstop[j] ; k++)
        lookup[j][k] = qMin(max_color, GLubyte((k-kstart[j]) * fCdel
                                          + min_color));
}

pID[0] = pImage[0];
pID[1] = pImage[1];
pID[2] = pImage[2];

for(int j = 0; j < nPixelsPerPlane; j++)
{
    if(bSingleImage)
    {
        GLubyte nVal;
        uint16 Iindex = *pID[0] - minval[0];
        if(bShowShadow)
        {
            if(hilite[j])
               nVal = int(150*float(Iindex)/255.0);
            else
               nVal = lookup[0][Iindex];
        }
        else
            nVal = lookup[0][Iindex];

        // convert colour index into RGB colour
        if(bLookAhead && m > 0)
        {
            *pBData = qMin(*pBData,redlut[nVal]);
            pBData++;
            *pBData = qMin(*pBData,greenlut[nVal]);
            pBData++;
            *pBData = qMin(*pBData,bluelut[nVal]);
            pBData++;

        }
        else
        {
            *pBData++=redlut[nVal];
            *pBData++=greenlut[nVal];
            *pBData++=bluelut[nVal];
        }
        pID[0]++;

    }
    else
    {
        GLubyte nVal[3] = {0, 0, 0};
        GLubyte cyan[3] = {245, 150, 150};

        for(int l = 0; l < nNoValidChannels; l++)
        {
```

```
                int m = nValidChannel[l];
                uint16 Iindex = *pID[m] - minval[m];
                if(bShowShadow)
                {
                    if(!hilite[j])
                        nVal[m] = int(cyan[m]*(float(*pID[m] - nFmin[m])/float(nFmax[m]-nFmin[m])));
                    else
                        nVal[m] = lookup[m][Iindex];
                }
                else
                    nVal[m] = lookup[m][Iindex];


            }


            if(bLookAhead)
            {
                if(m == 0 )
                {
                    if(cSumtype =='s')
                    {
                        *pBData++ = nVal[0]/nLookAhead;
                        *pBData++ = nVal[1]/nLookAhead;
                        *pBData++ = nVal[2]/nLookAhead;
                    }
                    else
                    {
                        *pBData++=nVal[0];  // red
                        *pBData++=nVal[1];  // green
                        *pBData++=nVal[2];  // blue
                    }
                }
                else
                {


                    if(cSumtype == 'm')
                    {
                        *pBData = qMin(*pBData,nVal[0]);
                        pBData++;
                        *pBData = qMin(*pBData,nVal[1]);
                        pBData++;
                        *pBData = qMin(*pBData,nVal[2]);
                        pBData++;
                    }
                    else
                    {
                        *pBData += nVal[0]/nLookAhead;
                        pBData++;
                        *pBData += nVal[1]/nLookAhead;
                        pBData++;
                        *pBData += nVal[2]/nLookAhead;
                        pBData++;
                    }


                }


            }
            else
            {
                *pBData++=nVal[0];  // red
                *pBData++=nVal[1];  // green
                *pBData++=nVal[2];  // blue
            }
            for(int l = 0; l < nNoValidChannels; l++)
            {
                int m = nValidChannel[l];
                pID[m]++;
            }
        }
    }
}
updateGL();
```

```
}
inline uint16 RyRFit::PixelVal(uint16* ps, QPoint point)
{
    int pval = (nPlaneNo-1)*nPixelsPerPlane+point.y()*nXdim+point.x();
    return (*(ps+pval));
}
QPoint RyRFit::RealPos(QPoint pnt)
{
//    QWidgetList scrollbars = imageScroll->scrollBarWidgets(Qt::AlignLeft | Qt::AlignRight |
//                                                  Qt::AlignTop | Qt::AlignBottom);
    QPoint Offset(0,0);
    QPoint Actual((pnt.x()+ Offset.x())/nZoom ,(pnt.y()+ Offset.y())/nZoom);


    if(Actual.x() < 0)Actual.setX(0);
    if(Actual.y() < 0)Actual.setY(0);
    if(Actual.x() >= nXdim)Actual.setX(nXdim - 1);
    if(Actual.y() >= nYdim)Actual.setY(nYdim - 1);
    return Actual;
}
//*************************************************************************
//*
//*  Section to handle tetramers
//*
//*************************************************************************
void RyRFit::showPlacement()
{
    TetramerPlacement->Show(nPlacementType);
}
void RyRFit::ChangeTetramerWidth()
{
    bool ok;
    double tsize = QInputDialog::getDouble(this, tr("Change Tetramerwidth"),
                                           tr("Tetramer width (nm):"), tetramersize, 24.0, 50.0, 1, &ok);
    if(!ok)
      return;
    tetramersize = tsize;
    tetramerwidth = tetramersize/fnmPerPixel;
    tetramerdiag = tetramerwidth/sqrt2;
    updateGL();
}
void RyRFit::setupSingleTetramer()
{
    nPlacementType = 1;
    nAddedTetramers = 1;
    tetramerwidth = tetramersize/fnmPerPixel;
    tetramerdiag = tetramerwidth/sqrt2;
}
void RyRFit::setupLaiChckrbrd()
{
    nPlacementType = 2;
    nAddedTetramers = 5;
    laicheckerboarddiag = (28.1/fnmPerPixel)/sqrt(2);
    tetramerwidth = 28.1/fnmPerPixel;
    tetramerdiag = laicheckerboarddiag;
    centredistance = 31.8/fnmPerPixel;
    baseangleinradians = acos(0.5);
}
void RyRFit::setupLaiSidebySide()
{
    nPlacementType = 3;
    nAddedTetramers = 9;
    laisidebysidediag = (28.8/fnmPerPixel)/sqrt(2);
    tetramerwidth = 28.8/fnmPerPixel;
    tetramerdiag = laisidebysidediag;
    centredistance = 30.1/fnmPerPixel;
    baseangleinradians = 0;
}
void RyRFit::setupCheckerboard(double tetrameroverlap)
{
    nPlacementType = 4;
    nAddedTetramers = 5;
    tetramerwidth = 29.0/fnmPerPixel;
    tetramerdiag = tetramerwidth/sqrt2;
```

```
    double offset = (tetrameroverlap/fnmPerPixel)-tetramerwidth;
    centredistance = sqrt(tetramerwidth*tetramerwidth+offset*offset);
    baseangleinradians = acos(offset/tetramerwidth);
}
void RyRFit::addTetramer()
{
    if(bClassifyTetramer || bModifyTetramer)
    {
      cerr << " Please complete classification or modification of tetramer before adding another one\n";
      return;
    }
    bAddTetramer = true;
    bPlacedTetramer=false;
    bDisplayNND = false;
    ntm[0].bStipple = false;
    for(int j=1; j < 9; j++)
        ntm[j].bStipple=true;
}
void RyRFit::modifyTetramer()
{
    if(bClassifyTetramer || bAddTetramer)
    {
      cerr << " Please complete classification or addition of tetramer before modifying another one\n";
      return;
    }
    bModifyTetramer = true;
    bDisplayNND = false;
}
void RyRFit::classifyTetramer()
{
    bClassifyTetramer = true;
    if(bModifyTetramer || bAddTetramer)
    {
      cerr << " Please complete addition or modification of the tetramer before classifying another one\n";
      return;
    }
    bDisplayNND = false;
}
void RyRFit::tetramerClassification(char c)
{
  // classification:
  // c = checkerboard
  // s = side by side
  // b = both checkerboard and side by side
  // i = isolated
  // l = Lai Checkerboard (28.1 nm tetramers)
  // L = Lai Side by side (28.8 nm tetramers)
  // u = unclassified


    char ttype[] = {'c', 's', 'b', 'i', 'u'};
    int k = -1;
    for(int i = 0; i < 5; i++)
    {
        if(c == ttype[i])
        {
            k = i;
            break;
        }
    }
    if(k == -1)
        c = 'u';
    tm[nBeingClassified].classification = c;
    nBeingClassified = -1;
    bClassifyTetramer = false;
    updateGL();
}
void RyRFit::saveTetramer()
{
    if(bAddTetramer)
    {
        if(nPlacementType == 3)
            TetramerAngle +=5;
```

```
        tm[nNoTetramers].classification = setType(nPlacementType);
        tm[nNoTetramers].centre=TetramerPosn;
        tm[nNoTetramers].angle=TetramerAngle;
        nNoTetramers++;
        for(int j = 1; j < nAddedTetramers; j++)
        {
            if(!ntm[j].bStipple)
            {
                tm[nNoTetramers].centre = QPoint(qRound(ntm[j].xpos/nZoom), qRound(ntm[j].ypos/nZoom));
                tm[nNoTetramers].angle = TetramerAngle;
                tm[nNoTetramers].classification = setType(nPlacementType);
                nNoTetramers++;
            }
        }
        bAddTetramer = false;
        bPlacedTetramer = false;
        for(int k=1; k < nAddedTetramers; k++)
            ntm[k].bStipple=true;
    }
    if(bModifyTetramer)
    {
        tm[nBeingModified].centre = TetramerPosn;
        tm[nBeingModified].angle = TetramerAngle;
        bModifyTetramer = false;
        nBeingModified = -1;
    }
    updateGL();
}
char RyRFit::setType(int nPlacementType)
{
    char classification;
    if(nPlacementType == 2)
        classification = 'l';
    else if(nPlacementType == 3)
        classification = 'L';
    else if(nPlacementType == 4)
        classification = 'c';
    else
        classification = 'u';


    return classification;
}
void RyRFit::showExtent()
{
    bShowArea = !bShowArea;
    if(bShowArea)
        calculateArea();
    updateGL();
}
void RyRFit::findTetramer(QPoint posn)
{
    float tmeas= tetramerwidth*tetramerwidth/4.0;


    if(bAddTetramer)
    {
        for(int j=1; j < nAddedTetramers; j++ )
        {
            float xdiff = (ntm[j].xpos - posn.x())/nZoom;
            float ydiff = (ntm[j].ypos - posn.y())/nZoom;
            float dist = xdiff*xdiff + ydiff*ydiff;
            if (dist < tmeas)
            {
                ntm[j].bStipple = !ntm[j].bStipple;
                updateGL();
                break;
            }
        }
    }
    else
    {
        for (int k = 0; k < nNoTetramers; k++)
        {
```

```
            QPoint t = tm[k].centre;
            float xdiff = (t.x() - posn.x()/nZoom);
            float ydiff = (t.y() - posn.y()/nZoom);
            float dist = xdiff*xdiff + ydiff*ydiff;
            if (dist < tmeas)
            {
                if(bModifyTetramer)
                {
                    nBeingModified = k;
                    TetramerPosn = tm[k].centre;
                    TetramerAngle = tm[k].angle;
                }
                if(bClassifyTetramer)
                    nBeingClassified = k;


                updateGL();
                break;
            }
        }
    }
}
void RyRFit::deleteTetramer()
{
    if(bAddTetramer)
    {
        bAddTetramer = false;
        bPlacedTetramer = false;
        for(int k=1; k < nAddedTetramers; k++)
            ntm[k].bStipple=true;
    }
    if(bModifyTetramer)
    {
        if(nBeingModified > -1)
        {
            for(int k = nBeingModified; k < nNoTetramers -1; k++ )
            {
                tm[k].centre = tm[k+1].centre;
                tm[k].angle = tm[k+1].angle;
            }
            nBeingModified = -1;
        }
        nNoTetramers--;
        bModifyTetramer = false;
    }
    updateGL();
}
void RyRFit::calculateArea()
{
    double x[4], y[4];
    double tdiag;
    std::vector <APoint> PixelPosn;


    for(int j = 0; j < nNoTetramers; j++)
    {
        float angleinradians = tm[j].angle*degtorad+fortyfivedeg;
        if(tm[j].classification == 'l')
            tdiag = laicheckerboarddiag;
        else if (tm[j].classification == 'L')
            tdiag = laisidebysidediag;
        else
            tdiag = tetramerdiag;


        float x1 = tdiag * cos (angleinradians);
        float y1 = tdiag * sin (angleinradians);
        float xpos = tm[j].centre.x();
        float ypos = tm[j].centre.y();
        x[0]=xpos + x1;
        y[0]=ypos + y1;
        x[1]=xpos - y1;
        y[1]=ypos + x1;
        x[2]=xpos - x1;
        y[2]=ypos - y1;
```

```
        x[3]=xpos + y1;
        y[3]=ypos - x1;


        PixelPosn.push_back(APoint(x[0],y[0]));
//        PixelPosn.push_back(APoint((x[0]+x[1])/2,(y[0]+y[1])/2));
        PixelPosn.push_back(APoint(x[1],y[1]));
//        PixelPosn.push_back(APoint((x[1]+x[2])/2,(y[1]+y[2])/2));
        PixelPosn.push_back(APoint(x[2],y[2]));
//        PixelPosn.push_back(APoint((x[2]+x[3])/2,(y[2]+y[3])/2));
        PixelPosn.push_back(APoint(x[3],y[3]));
//        PixelPosn.push_back(APoint((x[3]+x[0])/2,(y[3]+y[0])/2));
    }
    qSort(PixelPosn.begin(), PixelPosn.end(), RyRFit::PixelPosnLessThan);


    double nmlimit = 60;
    double cutoff = nmlimit/fnmPerPixel;
    int ngroups = 1;
    for(unsigned int j = 1; j < PixelPosn.size(); j++)
    {
        double xdiff = PixelPosn[j].x() - PixelPosn[j-1].x();
        if(xdiff > cutoff)
           ngroups++;
    }


    totalConvexArea = 0;
    totalAlphaArea = 0;
    segments.clear();
    cvexhull.clear();
    if(ngroups == 1)
    {
       FitShape(PixelPosn);
       totalConvexArea = AreaConvex;
       totalAlphaArea = AreaAlpha;
    }
    else
    {
       std::vector <APoint>* PixelGroups = new std::vector<APoint>[ngroups];
       int ng = 0;
       PixelGroups[ng].push_back(PixelPosn[0]);
       for(unsigned int j = 1; j < PixelPosn.size(); j++)
       {
          PixelGroups[ng].push_back(PixelPosn[j-1]);
          double xdiff = PixelPosn[j].x() - PixelPosn[j-1].x();
          if(xdiff > cutoff)
             ng++;
       }
       PixelGroups[ng].push_back(PixelPosn[PixelPosn.size()-1]);


       for(int m = 0; m < ngroups; m++)
       {
           FitShape(PixelGroups[m]);
           totalConvexArea += AreaConvex;
           totalAlphaArea += AreaAlpha;
       }
    }
    totalTetramerArea = tetramersize*tetramersize*nNoTetramers;
    percentTetramerAreaConvex = 100.0*totalTetramerArea/totalConvexArea;
    percentTetramerAreaAlpha = 100.0*totalTetramerArea/totalAlphaArea;
    TetramerDensityConvex = nNoTetramers/totalConvexArea;
    TetramerDensityAlpha = nNoTetramers/totalAlphaArea;
}
void RyRFit::FitShape(std::vector<APoint> PixelGroup)
{
    QVector &lt;Segment&gt; localsegments;
    Alpha_shape_2 A(PixelGroup.begin(), PixelGroup.end(), FT(1000), Alpha_shape_2::REGULARIZED);
    for(Alpha_shape_edges_iterator it =  A.alpha_shape_edges_begin(); it != A.alpha_shape_edges_end(); ++it)
    {
      localsegments.append(A.segment(*it));
      segments.append(A.segment(*it));
    }
```

```
// Order local segments
    int segsize=localsegments.size();
    for(int k = 0; k < segsize; k++)
    {
       for(int j=k+1; j < segsize; j++ )
       {
          if(localsegments[k].target() == localsegments[j].source())
          {
           if (j != k+1)
           {
             Segment temp = localsegments[k+1];
             localsegments[k+1] = localsegments[j];
             localsegments[j]=temp;
           }
           break;
          }
       }
    }


    Polygon_2 p;
    for(int k = 0; k < segsize; k++)
        p.push_back(localsegments[k].source());


    AreaAlpha = p.area()*fnmPerPixel*fnmPerPixel;


    std::vector<APoint> convex;
    CGAL::convex_hull_2(PixelGroup.begin(), PixelGroup.end(), std::back_inserter(convex));
    Polygon_2 q;
    for (std::vector<APoint>::iterator it = convex.begin() ; it != convex.end(); ++it)
        q.push_back(*it);


    QVector<APoint> convexh = QVector<APoint>::fromStdVector(convex);


    int csize=convexh.size();
    for(int k = 0; k < csize; k++)
    {
        int j = (k+1) % csize;
        Segment cvx(convexh[k],convexh[j]);
        cvexhull.append(cvx);
    }


    AreaConvex = q.area()*fnmPerPixel*fnmPerPixel;


//    std::cerr << "Areas : Convex Hull = " << AreaConvex << " - Alpha shape = " << AreaAlpha << "\n";


    return;
}
void RyRFit::PlotPerimeters()
{
//  Plot Alpha shape (concave hull)
    qglColor(Qt::cyan);
    glLineWidth(2.0);
    int nPerimPnts = segments.size();
    for(int j = 0; j < nPerimPnts; j++)
    {
        APoint ps = segments[j].source();
        APoint qs = segments[j].target();
        glBegin(GL_LINES);
          glVertex2d(ps.x()*nZoom, ps.y()*nZoom);
          glVertex2d(qs.x()*nZoom, qs.y()*nZoom);
        glEnd();
    }


// Plot convex hull


    qglColor(Qt::magenta);
    nPerimPnts = cvexhull.size();
    for(int j = 0; j < nPerimPnts; j++)
```

```
    {
        APoint ps = cvexhull[j].source();
        APoint qs = cvexhull[j].target();
        glBegin(GL_LINES);
          glVertex2d(ps.x()*nZoom, ps.y()*nZoom);
          glVertex2d(qs.x()*nZoom, qs.y()*nZoom);
        glEnd();
    }
    glLineWidth(1.0);
}
void RyRFit::calculateNearestNeighbours()
{
    nndmax = 0.0;
    nndmin = 1.e30;
// Because there are usually less than 25 tetramers in a dyad
// do a simple search rather than setting up a tree
    for(int j = 0; j < nNoTetramers; j++)
    {
        QPoint tmj = tm[j].centre;
        if(bFlip)
            tmj.setY(windowY-tmj.y());
        double xpos = tmj.x();
        double ypos = tmj.y();


        double nnd = 10000000;
        for(int i = 0; i < nNoTetramers; i++)
        {
            if(i == j) continue;
            QPoint tmi = tm[i].centre;
            if(bFlip)
                tmi.setY(windowY-tmi.y());


            double xd = xpos-tmi.x();
            double yd = ypos-tmi.y();
            double dist=sqrt(xd*xd + yd*yd);
            nnd = min(nnd,dist);
            if(dist == nnd)
                nnLine[j] = QLine(tmj, tmi);
        }
        mnnd[j] = float(nnd)*fnmPerPixel;
        nndmax = qMax(nndmax, mnnd[j]);
        nndmin = qMin(nndmin, mnnd[j]);
    }
    bDisplayNND = true;
    if(!bAnalyseOnly)
       updateGL();
}
void RyRFit::showNearestNeighbours()
{
    bDisplayNND = !bDisplayNND;
    if(bDisplayNND)
    {
      calculateNearestNeighbours();
      showNearestNeighboursAct->setText("Hide Nearest Neighbours {n}");
    }
    else
      showNearestNeighboursAct->setText("Show Nearest Neighbours {n}");


    updateGL();
}
void RyRFit::changeShadowOutline()
{
    if(ShadowShape == SQUARE)
    {
        ShadowShape = CIRCLE;
        changeShadowAct->setText("Square Shadow {F3}");
    }
    else
    {
        ShadowShape= SQUARE;
        changeShadowAct->setText("Circular Shadow {F3}");
    }
```

```
    if(bFlood)
    {
        bFloodCalculated = false;
        ComputeFloodfill();
    }
}
void RyRFit::showRedTetramers()
{
    bSingleColor = !bSingleColor;
    if(bSingleColor)
       showRedTetramersAct->setText("Classification Colours {F1}");
    else
       showRedTetramersAct->setText("Red Tetramers {F1}");
    updateGL();
}
void RyRFit::hideTetramers()
{
    bShowTetramers = !bShowTetramers;
    if(bShowTetramers)
       showTetramersAct->setText("Hide Tetramers {F2}");
    else
       showTetramersAct->setText("Show Tetramers {F2}");


    updateGL();
}
void RyRFit::setFloodFillType()
{
    bPartialFlood = !bPartialFlood;
    if(bPartialFlood)
       FloodFillTypeAct->setText("FloodFill = Complete {F7}");
    else
       FloodFillTypeAct->setText("FloodFill = Surround {F7}");


    if(bFlood)
    {
      bFloodCalculated=false;
      ComputeFloodfill();
    }
}
void RyRFit::hideEMImage()
{
    bHideEMImage = !bHideEMImage;
    if(bHideEMImage)
        hideEMImageAct->setText("Show EM Image {h}");
    else
        hideEMImageAct->setText("Hide EM Image {h}");
    updateGL();
}
void RyRFit::FlipImage()
{
    bFlip = !bFlip;
    if(bDisplayNND)
        calculateNearestNeighbours();


    updateGL();
}
void RyRFit::DrawContour()
{
    if(bFlood)
    {
       bFlood = false;
       bFloodCalculated = false;
       CalculateBitmap();
    }
    FloodAct->setEnabled(false);
    toggleShadowAct->setEnabled(false);
    bShowShadow = false;
    bDrawing = true;
    bFlood = false;
    bFloodCalculated = false;
    nPerPnts = 0;
    bAddTetramer = false;
```

```
    bModifyTetramer = false;
    bClassifyTetramer = false;
}
void RyRFit::toggleShadow()
{
    if(!bFloodCalculated)
        return;
    bShowShadow = !bShowShadow;
    if(bShowShadow)
        toggleShadowAct->setText("Hide Shadow {F4}");
    else
        toggleShadowAct->setText("Show Shadow {F4}");
    CalculateBitmap();
    updateGL();
}
void RyRFit::ComputeFloodfill()
{
    if(bFloodCalculated)
    {
        if(bFlood)
        {
           FloodAct->setText("Isolate Region {F9}");
           bFlood = false;
        }
        else
        {
           FloodAct->setText("Show All Tetramers {F9}");
           bFlood = true;
        }


        CalculateBitmap();
        return;
    }
    if(nPerPnts < 5)
    {
        cerr << "Contour missing/not large enough\n";
        return;
    }
    // create plane for copy
    int size = nXdim*nYdim;
    int* plane = new int[size];
    if(plane == 0)
    {
        cerr << "Cannot create space for image!" << endl;
        return;
    }
    memset(plane,0,size*4);
    QPoint p[5];


    int* pi = plane;
    QPoint* point = PerPnt;
    *(pi + point->y()*nXdim + point->x()) = 1;


    double dLength = 0.0;
    bool bStatus = false;
    // make sure we have a closed loop
    PerPnt[nPerPnts] = PerPnt[0];
    nPerPnts++;
    // copy perimeter - we're not interested in the image
    for (int k = 1; k < nPerPnts; k++)
    {
        QPoint *next = point + 1;
        int xdiff = next->x() - point->x();
        int ydiff = next->y() - point->y();
        double dist = sqrt(double(xdiff)*double(xdiff) + double(ydiff)*double(ydiff));
        dLength += dist;
        int idist = max(abs(xdiff),abs(ydiff));
        if(idist == 1)
            *(pi + (next->y())*nXdim + next->x()) = 1;
        else
        {
            // fill in gaps in perimeter
```

```
        float xp = point->x() + 0.5;
        float yp = point->y() + 0.5;
        float dist = float(idist);
        float dx = float(xdiff) / dist;
        float dy = float(ydiff) / dist;
        for(int i = 0; i <  idist; i++)
        {
            xp += dx;
            yp += dy;
            *(pi + int(yp)*nXdim + int(xp)) = 1;
        }
    }
    point = next;
}


if(!DoFloodFill(plane))
    goto cleanup2;


if(bPartialFlood)
    goto dignified;


memset(plane,0,size*4);


for(int i = 0; i < nNoTetramers; i++)
{
    double tdiag;
    double angleinradians = tm[i].angle*degtorad+fortyfivedeg;


    if(tm[i].classification == 'l')
        tdiag = laicheckerboarddiag;
    else if(tm[i].classification == 'L')
        tdiag = laisidebysidediag;
    else
        tdiag = tetramerdiag;


    double xpos = tm[i].centre.x();
    double ypos = tm[i].centre.y();
    if(ShadowShape == SQUARE)
    {
        double x1 = (tdiag * cos (angleinradians));
        double y1 = (tdiag * sin (angleinradians));
        p[0] = QPoint(qRound(xpos + x1), qRound(ypos + y1)) ;
        p[1] = QPoint(qRound(xpos - y1), qRound(ypos + x1));
        p[2] = QPoint(qRound(xpos - x1), qRound(ypos - y1));
        p[3] = QPoint(qRound(xpos + y1), qRound(ypos - x1));
        if(hilite[p[0].x()+nXdim*p[0].y()])continue;
        if(hilite[p[1].x()+nXdim*p[1].y()])continue;
        if(hilite[p[2].x()+nXdim*p[2].y()])continue;
        if(hilite[p[3].x()+nXdim*p[3].y()])continue;
        p[4] = p[0];
        for (int k = 0; k < 4; k++)
        {
            *(pi + (p[k].y())*nXdim + p[k].x()) = 1;
            QPoint next = p[k+1];
            int xdiff = next.x() - p[k].x();
            int ydiff = next.y() - p[k].y();
            double dist = sqrt(double(xdiff)*double(xdiff) + double(ydiff)*double(ydiff));
            dLength += dist;
            int idist = max(abs(xdiff),abs(ydiff));
            if(idist == 1)
                *(pi + (next.y())*nXdim + next.x()) = 1;
            else
            {
                // fill in gaps in perimeter
                float xp = p[k].x() + 0.5;
                float yp = p[k].y() + 0.5;
                float dist = float(idist);
                float dx = float(xdiff) / dist;
                float dy = float(ydiff) / dist;
                for(int i = 0; i <  idist; i++)
```

```
                    {
                        xp += dx;
                        yp += dy;
                        *(pi + int(yp)*nXdim + int(xp)) = 1;
                    }
                }
            }
        }
        else
        {
            int num_segments = 200;
            float theta = 2 * 3.1415926 / float(num_segments);
            float tangential_factor = tanf(theta);//calculate the tangential factor

            float radial_factor = cosf(theta);//calculate the radial factor

            float x = tdiag;//we start at angle = 0

            float y = 0;

            if(hilite[qRound(xpos)+nXdim*qRound(ypos)])continue;
            double xpos = tm[i].centre.x();
            double ypos = tm[i].centre.y();
            *(pi + int(ypos)*nXdim +int(xpos + x)) = 1;
            for (int k = 0; k < num_segments; k++)
            {
                float tx = -y;
                float ty = x;

                //add the tangential vector

                x += tx * tangential_factor;
                y += ty * tangential_factor;

                //correct using the radial factor

                x *= radial_factor;
                y *= radial_factor;
                *(pi + int(ypos+y)*nXdim +int(xpos + x)) = 1;
            }
        }
    }

    for(int j=0; j < nXdim*nYdim; j++)
        hilite[j] = false;

    if(!DoFloodFill(plane))
        goto cleanup2;

 //    cerr << "Completed Flood fill\n";
dignified:
    bFloodCalculated = true;
    bFlood = true;
    toggleShadowAct->setEnabled(true);
    changeShadowAct->setEnabled(true);
    FloodAct->setText("Show All Tetramers {F9}");
    bDrawing = false;
    bStatus = true;
cleanup2:
    delete [] plane;
    if(bStatus)
        CalculateBitmap();
}
bool RyRFit::DoFloodFill(int* plane)
{
    // Now do flood fill
    int boundary = 1;
```

```
int flood_fill = 2;
int background = 0;

//   Create outer border to confine flood-fill

bool bStatus = false;
int* pi = plane;
for(int iy = 0; iy < nYdim; iy++)
{
    int lo = iy*nXdim;
    *(pi + lo) = boundary;
    *(pi + nXdim-1 + lo) = boundary;
}
int bl = (nYdim -1)*nXdim;
for(int ix = 0; ix < nXdim; ix++)
{
    *(pi + ix) = boundary;
    *(pi + ix + bl) = boundary;
}

int size = nXdim*nYdim;
QPoint* store = new QPoint[size];
QPoint* stack = store;
for(int i = 0; i < size; i++)
{
    store->setX(0);
    store->setY(0);
    store++;
}

//   start at (1,1) - outside of image area

store = stack;
store->setX(1);
store->setY(1);

int iptr = 1;
bool flag = true;

//    cerr << "Flood fill initialized\n";
while (flag)
{
    //          pop stack
    iptr--;
    if (iptr < 0)
    {
        cerr << "Pointer travelled past begining of array!\n";
        bStatus = false;
        goto cleanup;
    }
    stack = store + iptr;
    int ix = stack->x();
    int iy = stack->y();
    int *pm = plane + ix + iy*nXdim;
    if (*pm == background)
        *pm = flood_fill;

    //     If central pixel is painted, check its neighbors.
    //     A neighbor is pushed on stack if it is not painted before
    //     and its not a boundary pixel

    if (*pm == flood_fill)
    {
        int ia = *(pm+1);
        int ic = *(pm-nXdim);
        int ie = *(pm-1);
        int ig = *(pm+nXdim);
        if(ia == background)
```

```
            {
                stack->setX(ix+1);
                stack->setY(iy);
                iptr++;
                stack = store + iptr;
                if (iptr > size)
                {
                    cerr << "Pointer travelled past end of array!\n";
                    bStatus = false;
                    goto cleanup;
                }
            }


            if(ic == background)
            {
                stack->setX(ix);
                stack->setY(iy-1);
                iptr++;
                stack = store + iptr;
                if (iptr > size)
                {
                    cerr << "Pointer travelled past end of array!\n";
                    bStatus = false;
                    goto cleanup;
                }
            }
            if(ie == background)
            {
                stack->setX(ix-1);
                stack->setY(iy);
                iptr++;
                stack = store + iptr;
                if (iptr > size)
                {
                    cerr << "Pointer travelled past end of array!\n";
                    bStatus = false;
                    goto cleanup;
                }
            }
            if(ig == background)
            {
                stack->setX(ix);
                stack->setY(iy+1);
                iptr++;
                if (iptr > size)
                {
                    cerr << "Pointer travelled past end of array!\n";
                    bStatus = false;
                    goto cleanup;
                }
            }
        }
        if(iptr == 0) flag = false;
    }
    //   compute overlay array
    for (int iy = 1; iy < nYdim - 1; iy++)
    {
        int iyx = iy*nXdim;
        for (int ix = 1; ix < nXdim - 1; ix++)
        {
            int offset = ix + iyx ;
            if (*(plane + offset) == flood_fill)
                hilite[offset] = true;
            else
                hilite[offset] = false;
        }
    }
    bStatus = true;
cleanup:
    delete [] store;
    return bStatus;
}
```

```
//*****************************************
//* End Section to deal with Tetramers
//*****************************************
void RyRFit::getScaleBarSize()
{
   bScaleBar=!bScaleBar;
   if(bScaleBar)
   {
     int minsize=20;
     nScaleBarLength=QInputDialog::getInt(nullptr,"ScaleBar Value","Enter Scalebar length (nm)", nScaleBarLength, minsize, 500, 10);
     ScaleBarAct->setText("Hide Scale Bar {F6}");
   }
   else
     ScaleBarAct->setText("Display Scale Bar (size in nm) {F6}");
   updateGL();
}
void RyRFit::HideZ()
{
   bShowZ = !bShowZ;
   updateGL();
}
void RyRFit::AutoScale()
{
   if(bAutoScale)return;
   bAutoScale = true;
   bScale[0]=bScale[1]=bScale[2]=false;
   CalculateBitmap();
   ScaleVal->hide();
}
void RyRFit::FixedScale()
{
   if(!bAutoScale)return;
   bAutoScale = false;
   bScale[0]=bScale[1]=bScale[2]=true;
   CalculateBitmap();

   ScaleVal->moveImage(imageScroll->x(), imageScroll->y(),
                    (imageScroll->frameGeometry()).width());
   ScaleVal->Show(nSmin, nSmax);
}
void RyRFit::toggleScale(bool bState)
{
   bScale[0] = bScale[1] = bScale[2] = bState;

   if(!bState)
      CalculateBitmap();
}
void RyRFit::ChangeScale(int* nLow, int* nHi)
{
   for(int i=0; i < nNoChannels; i++)
   {
      nSmin[i]=nLow[i];
      nSmax[i]=nHi[i];
   }
   CalculateBitmap();
}


// colourmaps for non-RGB single channel images


void RyRFit::colourMono()
{
   CreateColourMap(MONO);
   CalculateBitmap();
}
void RyRFit::colourRed()
{
   CreateColourMap(RED);
   CalculateBitmap();
}
void RyRFit::colourGreen()
{
   CreateColourMap(GREEN);
```

```
    CalculateBitmap();
}
void RyRFit::colourBlue()
{
    CreateColourMap(BLUE);
    CalculateBitmap();
}
void RyRFit::colourInvMono()
{
    CreateColourMap(INVMONO);
    CalculateBitmap();
}
void RyRFit::colourLUT()
{
    CreateColourMap(LUT);
    CalculateBitmap();
}
void RyRFit::imageZoomIn()
{
    nOldZoom = nZoom;
    if(nZoom < 8)nZoom++;
    ChangeSize();
}
void RyRFit::imageZoomOut()
{
    nOldZoom = nZoom;
    if(nZoom > 1)nZoom--;
    ChangeSize();
}
void RyRFit::ChangeSize()
{
    nImageXdim = nZoom*nXdim;
    nImageYdim = nZoom*nYdim;
    resize(nImageXdim,nImageYdim);
    windowY = nImageYdim;
    //    sysfont->setPixelSize(15);
    float factor=float(nZoom)/float(nOldZoom);


    if(bLinePlot)
    {
        StartPt = QPoint(int(StartPt.x()*factor),int(StartPt.y()*factor));
        EndPt = QPoint(int(EndPt.x()*factor),int(EndPt.y()*factor));
    }
    resizeGL(nImageXdim,nImageYdim);
    imageScroll->resize(nImageXdim+6,nImageYdim+6);
    updateGL();
}
void RyRFit::imageDisplay()
{
    CalculateBitmap();
}
void RyRFit::imageFirstPlane()
{
// display first plane from EM image
    nPlaneNo=nZmin;
    imageDisplay();
}
void RyRFit::imageLastPlane()
{
// display last plane from EM image
    nPlaneNo=nZmax;
    imageDisplay();
}
void RyRFit::imageNextPlane()
{
// display next plane from EM image
    nPlaneNo++;
    nPlaneNo = (nPlaneNo > nZmax) ? nZmin : nPlaneNo; // cycle to beginning
    imageDisplay();
}
void RyRFit::imagePrevPlane()
{
// display prev plane from EM image
```

```
    nPlaneNo--;
    nPlaneNo = (nPlaneNo < nZmin) ? nZmax : nPlaneNo; // cycle to end
    imageDisplay();
}
void RyRFit::closeEvent(QCloseEvent* event)
{
    if(bAddTetramer || bModifyTetramer || bClassifyTetramer)
    {
          cerr << "Cannot - close - Tetramer is being added, modified or classified\n";
          event->ignore();
    }
    else
    {
       CloseProgram();
       event->accept();
    }
}
void RyRFit::CloseProgram()
{
// calculate print and save output
    if(nNoTetramers > 0)
    {


        int nNoUnclassified = 0;
        int nNoCheckerboard = 0;
        int nNoSidebySide = 0;
        int nNoIsolated = 0;
        int nNoBoth = 0;
        int nLaiCheckerboard = 0;
        int nLaiSidebySide = 0;
        if(!bAnalyseOnly)
        {
            cerr << "Saving tetramer file: " << qPrintable(DataFileName) << " ...\n";
            QFile file(DataFileName);
            if(!file.open(QIODevice::WriteOnly | QIODevice::Text))
            {
                QMessageBox::information(0,"error",file.errorString());
                goto closeit;
            }


            QTextStream out(&file);
            out << "scale=" << fnmPerPixel << "\n";


            for (int jj = 0; jj < nNoTetramers; jj++)
              out << tm[jj].centre.x() << ' '<< tm[jj].centre.y() << ' ' << tm[jj].angle << ' ' << tm[jj].classification <<"\n";


            file.close();
        }
        for (int jj = 0; jj < nNoTetramers; jj++)
        {
            switch(tm[jj].classification)
            {
            case 'u':
                nNoUnclassified++;
                break;
            case 'c':
                nNoCheckerboard++;
                break;
            case 's':
                nNoSidebySide++;
                break;
            case 'i':
                nNoIsolated++;
                break;
            case 'b':
                nNoBoth++;
                break;
            case 'l':
                nLaiCheckerboard++;
                break;
            case 'L':
```

```
            nLaiSidebySide++;
            break;
        default:
            qglColor(Qt::white);
        }
    }


    if(totalConvexArea == 0.0)
        calculateArea();


    calculateNearestNeighbours();


    QFile nnfile(NNFileName);  // nearest neighbour distance file
    if(!nnfile.open(QIODevice::WriteOnly | QIODevice::Text))
    {
        QMessageBox::information(nullptr,"error",nnfile.errorString());
        goto closeit;
    }
    QTextStream nnout(&nnfile);


// output to both file and screen


    nnout << "Total tetramers = " << nNoTetramers << "\n";
    cerr << "\nTotal tetramers = " << nNoTetramers << "\n";
    if(nNoCheckerboard > 0)
    {
       cerr << "No. checkerboard = " << nNoCheckerboard << "\n";
       nnout << "No. checkerboard = " << nNoCheckerboard << "\n";
    }
    if(nNoSidebySide > 0)
    {
       cerr << "No. side by side = " << nNoSidebySide << "\n";
       nnout << "No. side by side = " << nNoSidebySide << "\n";
    }
    if(nNoIsolated > 0)
    {
       cerr << "No. Isolated = " << nNoIsolated << "\n";
       nnout << "No. Isolated = " << nNoIsolated << "\n";
    }
    if(nNoBoth > 0)
    {
       cerr << "No. Both = " << nNoBoth << "\n";
       nnout << "No. Both = " << nNoBoth << "\n";
    }
    if(nLaiCheckerboard > 0)
    {
       cerr << "No. Lai Checkerboard = " << nLaiCheckerboard << "\n";
       nnout << "No. Lai Checkerboard = " << nLaiCheckerboard << "\n";
    }
    if(nLaiSidebySide > 0)
    {
       cerr << "No. Lai Side by Side = " << nLaiSidebySide << "\n";
       nnout << "No. Lai Side by Side = " << nLaiSidebySide << "\n";
    }
    if(nNoUnclassified > 0)
    {
       cerr << "No. Unclassified = " << nNoUnclassified << "\n";
       nnout << "No. Unclassified = " << nNoUnclassified << "\n";
    }


    cerr << "\nNearest Neighbour distances\n";
    cerr << setprecision(2) << fixed;


    if(nNoCheckerboard > 0)
    {
        cerr << "\nCheckerboard\n";
        nnout << "\nCheckerboard\n";
        for(int k= 0; k < nNoTetramers; k++)
        {
            if(tm[k].classification == 'c')
```

```
            {
                nnout << mnnd[k] << "\n";
                cerr << mnnd[k] << "\n";
            }
        }
        cerr << "\n";
    }
    if(nNoSidebySide > 0)
    {
        cerr << "\nSide by side\n";
        nnout << "\nSide by side\n";
        for(int k= 0; k < nNoTetramers; k++)
        {
            if(tm[k].classification == 's')
            {
                nnout << mnnd[k] << "\n";
                cerr << mnnd[k] << "\n";
            }
        }
        cerr << "\n";
    }
    if(nNoBoth > 0)
    {
        cerr << "\nBoth\n";
        nnout << "\nBoth\n";
        for(int k= 0; k < nNoTetramers; k++)
        {
            if(tm[k].classification == 'b')
            {
                nnout << mnnd[k] << "\n";
                cerr << mnnd[k] << "\n";
            }
        }
        cerr << "\n";
    }
    if(nNoIsolated > 0)
    {
        cerr << "\nIsolated\n";
        nnout << "\nIsolated\n";
        for(int k= 0; k < nNoTetramers; k++)
        {
            if(tm[k].classification == 'i')
            {
                nnout << mnnd[k] << "\n";
                cerr << mnnd[k] << "\n";
            }
        }
        cerr << "\n";
    }
    if(nLaiCheckerboard > 0)
    {
        cerr << "\nLai checkerboard\n";
        nnout << "\nLai checkerboard\n";
        for(int k= 0; k < nNoTetramers; k++)
        {
            if(tm[k].classification == 'l')
            {
                nnout << mnnd[k] << "\n";
                cerr << mnnd[k] << "\n";
            }
        }
        cerr << "\n";
    }
    if(nLaiSidebySide > 0)
    {
        cerr << "\nLai side by side\n";
        nnout << "\nLai side by side\n";
        for(int k= 0; k < nNoTetramers; k++)
        {
            if(tm[k].classification == 'L')
            {
                nnout << mnnd[k] << "\n";
                cerr << mnnd[k] << "\n";
```

```
                }
            }
            cerr << "\n";
        }
        if(nNoUnclassified > 0)
        {
            cerr << "\nUnclassified\n";
            nnout << "\nUnclassified\n";
            for(int k= 0; k < nNoTetramers; k++)
            {
                if(tm[k].classification == 'u')
                {
                   nnout << mnnd[k] << "\n";
                   cerr << mnnd[k] << "\n";
                }
            }
            cerr << "\n";
        }


        nnout.setRealNumberPrecision(2);
        nnout << "\nConvex Hull: Tetramers cover " << fixed << percentTetramerAreaConvex  << " % of the total area - Density = ";
        nnout.setRealNumberPrecision(3);
        nnout << scientific << TetramerDensityConvex  << " nm**-2\n";
        nnout.setRealNumberPrecision(2);
        nnout << "Alpha Shape: Tetramers cover " << fixed << percentTetramerAreaAlpha  << " % of the total area - Density = ";
        nnout.setRealNumberPrecision(3);
        nnout << scientific << TetramerDensityAlpha  << " nm**-2\n";
        nnfile.close();


        cerr << "Saving nearest neighbour file : " << qPrintable(NNFileName) << " ...\n";


        cerr << "No. of Tetramers: " << nNoTetramers << "\n";
        cerr << "ConvexArea = " << totalConvexArea << " - AlphaArea = " << totalAlphaArea << " nm2\n";
        cerr << "Convex Hull: Tetramers cover " << fixed << setprecision(2) << percentTetramerAreaConvex  << " % of the total area - Density = " <<
scientific << setprecision(3) << TetramerDensityConvex  << " nm**-2\n";
        cerr << "Alpha Shape: Tetramers cover " << fixed << setprecision(2) << percentTetramerAreaAlpha  << " % of the total area - Density = " <<
scientific << setprecision(3) << TetramerDensityAlpha  << " nm**-2\n";
    }
closeit:
    if(bAnalyseOnly)
       exit(0);
    else
    {
       ScaleVal->close();
       imageScroll->close();
    }
}
void RyRFit::Instructions(void)
{
    cerr << "\nAdding and modifying tetramers\n\n";
    cerr << "Adding a tetramer\n";
    cerr << "Press the 'a' key and left click the mouse in the approximate position\n";
    cerr << "you want the tetramer to appear. The position of the tetramer can be moved\n";
    cerr << "one pixel at a time using the four arrow keys on the keyboard. The rotation\n";
    cerr << "is controlled by the mouse wheel. Once you are satisfied with the position\n";
    cerr << "press the 's' key to save the tetramer position\n\n";
    cerr << "Modifying a tetramer\n";
    cerr << "If, after other tetramers have been placed, there is conflict or overlap and you want\n";
    cerr << "to alter the position, press the 'm' key and left click on the desired tetramer, then\n";
    cerr << "proceed as before, using the arrow keys and the mouse wheel. Press the 's' key once finished\n\n";
    cerr << "Delete a Tetramer\n";
    cerr << "To delete a tetramer, press the 'd' key and left click on the desired tetramer, and it will be\n";
    cerr << "erased - Warning - this cannot be undone!\n\n";
    cerr << "Classify a Tetramer\n";
    cerr << "To classify a tetramer, press the 'c' key and left click on the desired tetramer, then use one of the\n";
    cerr << "following keys to classify it according to the scheme and it will be\n";
    cerr << "   c = checkerboard\n";
    cerr << "   s = side by side\n";
    cerr << "   b = both checkerboard and side by side(two or more different types abutting the same tetramer)\n";
    cerr << "   i = isolated\n";
    cerr << "   u = unclassified (default for newly added tetramers)\n\n";
    cerr << "By default you add a single tetramer each time you press A, however it is possible to add groups\n";
```

```
    cerr << "of tetramers by pressing 'T' which will bring up a window with the following choices:\n";
    cerr << "  1. a single tetramer (default)\n";
    cerr << "  2. a group of 5 tetramers that follows the Lai checkerboard arrangement\n";
    cerr << "  3. a group of 9 tetramers that follows the Lai side-by-side arrangement\n";
    cerr << "  4. a group of 5 tetramers that follows a checkerboard arrangement with a user-defined overlap (nm)\n";
    cerr << "     Overlap is the amount of overlap (in nm) of two tetramers\n";
    cerr << "\nKeys:\n\n";
    cerr << "\ta - Add Tetramer\n";
    cerr << "\tb - when classifying tetramer - classification = 'both'\n";
    cerr << "\tc - Classify Tetramer\n";
    cerr << "\t  - when classifying tetramer - classification = 'checkerboard'\n";
    cerr << "\td - Delete Tetramer\n";
    cerr << "\th - Show/Hide EM Image (toggle)\n";
    cerr << "\ti - when classifying tetramer - classification = 'isolated'\n";
    cerr << "\tk - If look ahead is on - Minimum/Summed Intensity for each pixel (toggle)\n";
    cerr << "\tl - Look ahead On/Off (default 4 planes)\n";
    cerr << "\t1,2...9 - If Look Ahead is on show 1 to 9 extra planes at a time\n";
    cerr << "\tm - Modify Tetramer\n";
    cerr << "\tn - Show/Hide Nearest Neighbours distances (toggle)\n";
    cerr << "\tp - Measure Distance - press p then left click and drag mouse between points\n";
    cerr << "\tq - Hide/Unhide Frame No. (toggle)\n";
    cerr << "\tr - Show/Hide Alpha Shape & Hull(toggle)\n";
    cerr << "\ts - Save Tetramer after addition or mdoification\n";
    cerr << "\t  - when classifying tetramer - classification = 'side-by-side'\n";
    cerr << "\tt - Change Tetramer placement from single to one of different groups\n";
    cerr << "\tu - when classifying tetramer - classification = 'unclassified'\n";
    cerr << "\tw - Change tetramer width\n";
    cerr << "\tz - Toggle Frame display\n";
    cerr << "\tF1 - Red/Colored Tetramers (toggle)\n";
    cerr << "\tF2 - Show/Hide Tetramers (toggle)\n";
    cerr << "\tF3 - Circular/Square Shadow (toggle)\n";
    cerr << "\tF4 - Show/Hide Tetramer Shadow (toggle)\n";
    cerr << "\tF5 - Outline Region (draw contour) - can create sub-regions of the dyad\n";
    cerr << "\tF6 - Display Scale Bar (size in nm) (toggle)\n";
    cerr << "\tF7 - FloodFill = Surround/Complete (toggle)\n";
    cerr << "\tF9 - Isolate Region\n";
    cerr << "\tF10 - Print help\n";
    cerr << "\tF11 - Change Tetramer Width\n";
    cerr << "\tF12 - Flip Image\n";
    cerr << "\t+ - Zoom In\n";
    cerr << "\t- - Zoom Out\n";
    cerr << "\tHome - move image to 1st plane\n";
    cerr << "\tEnd - move image to last plane\n";
    cerr << "\tEsc - Quit program - while drawing quit drawing and erase line\n";
    cerr << "\tArrow keys (left, right, up and down) - adding or modifying the tetramer\n";
    cerr << "              keys will move the tetramer 1 pixel in the direction pressed\n";
    cerr << "              When viewing the image, the left arrow will move the image 1 plane\n";
    cerr << "              back, while the right arrow will move it 1 plane forward\n";
    cerr << "\nMouse wheel - when adding or modifying rotate tetramers\n";
    cerr << "To draw contours to isolate a region mouse middle button down and then trace path\n";
    cerr << "\nMany of the above can be accessed through the menu (mouse right click)\n";
    cerr << "For monochrome images there are colour options that can only be accessed through the menu\n";
    cerr << endl;
}
bool RyRFit::ParseCmdString(int argc, char** argv)
{
    // Parse the command line for options and images
    nNoStacks=0;
    bool bStack = false;


    int i=1;
    while(i < argc)
    {
        if (*argv[i] == '-')
        {
            if(strlen(argv[i]) == 1)
            {
                if(bStack)
                    FileName[nNoStacks-1].append(argv[i]);
                else
                    cerr << "\n Warning - Isolated dash in input - ignored\n";
                i++;
                continue;
```

```
            }
            bStack = false;
        }
        else
        {
            if(bStack)
            {
                FileName[nNoStacks-1].append(argv[i]);
                //      cerr << "i << " " << argv[i] << " " << qPrintable( FileName[nNoStacks-1].last()) << "\n";
            }
            else
                cerr << "\n Warning - Isolated filename = " << argv[i] <<" in input - ignored\n";
            i++;
            continue;
        }
        if ( !strcmp("-R", argv[i]) )
        {
            bAnalyseOnly = true;
        }
        else if ( !strcmp("-M", argv[i]) )
        {
            nZoom = atoi( argv[++i] );
            if ( nZoom < 1  || nZoom > 8)
            {
                cerr << "\n Warning! - Invalid zoom value: -M " << nZoom << endl;
                nZoom = 1;
            }
        }
        else if ( !strcmp("-W", argv[i]) )
        {
           tetramersize = atof(argv[++i]);
           if (tetramersize < 20.0 || tetramersize > 50.0)
           {
               cerr << "\n Warning! - Invalid value: -W " << tetramersize << "set to 27 nm " << endl;
               tetramersize = 27.0;
           }
           else
             cerr << "\n Tetramer width set to " << tetramersize << endl;
        }
        else if ( !strcmp("-scale", argv[i]))
        {
            fnmPerPixel = atof(argv[++i]);
            bEnteredScale = true;
        }
        else if ( !strcmp("-I", argv[i]) )
        {
            nNoStacks++;
            if(nNoStacks == 4)
            {
                cerr << "\n Error! - can only enter a maximum of three images or stacks\n";
                return false;
            }
            bStack = true;
        }
        else
            cerr << "\n Warning! - argument " << i << " \'" <<argv[i] << "\' isn\'t a valid option\n";
        i++;
    }


    if(bAnalyseOnly)
        return true;


    if(nNoStacks == 0 )
    {
        cerr <<  "\n Error! - Missing image name !\n" << endl;
        return false;
    }


    return true;
}
void RyRFit::createMenus()
{
```

```
    // Image Menu

    imageMenu->addAction(hideAct);
    tetramerMenu=imageMenu->addMenu(tr("Tetramers"));
    tetramerMenu->addAction(addTetramerAct);
    tetramerMenu->addAction(deleteTetramerAct);
    tetramerMenu->addAction(modifyTetramerAct);
    tetramerMenu->addAction(saveTetramerAct);
    tetramerMenu->addAction(showTetramerExtentAct);
    tetramerMenu->addAction(showRedTetramersAct);
    tetramerMenu->addAction(showTetramersAct);
    tetramerMenu->addAction(changeShadowAct);
    tetramerMenu->addAction(toggleShadowAct);
    tetramerMenu->addAction(DrawContourAct);
    tetramerMenu->addAction(FloodFillTypeAct);
    tetramerMenu->addAction(FloodAct);
    tetramerMenu->addAction(showNearestNeighboursAct);
    tetramerMenu->addAction(changePlacementAct);
    tetramerMenu->addAction(hideEMImageAct);
    tetramerMenu->addAction(ChangeTetramerWidthAct);
    tetramerMenu->addAction(FlipImageAct);

    iscaleMenu=imageMenu->addMenu(tr("Scaling"));
    iscaleMenu->addAction(autoAct);
    iscaleMenu->addAction(fixedAct);

    if(nNoChannels == 1)
    {
        colourMenu=imageMenu->addMenu(tr("Colour"));
        colourMenu->addAction(colbwAct);
        colourMenu->addAction(colredAct);
        colourMenu->addAction(colgreenAct);
        colourMenu->addAction(colblueAct);
        colourMenu->addAction(colwbAct);
        colourMenu->addAction(colLUTAct);
    }

    imageMenu->addAction(changeLookAheadAct);
    imageMenu->addAction(LineAct);
    imageMenu->addAction(ScaleBarAct);
    imageMenu->addSeparator();
    imageMenu->addAction(ZoomInAct);
    imageMenu->addAction(ZoomOutAct);
    imageMenu->addSeparator();
    imageMenu->addAction(HelpAct);
    imageMenu->addSeparator();
    imageMenu->addAction(QuitAct);

}
void RyRFit::createActions()
{
    // image menu actions
    addTetramerAct = new QAction(tr("Add {a}"),this);
    connect(addTetramerAct, SIGNAL(triggered()), this, SLOT(addTetramer()));

    modifyTetramerAct = new QAction(tr("Modify {m}"),this);
    connect(modifyTetramerAct, SIGNAL(triggered()), this, SLOT(modifyTetramer()));

    deleteTetramerAct = new QAction(tr("Delete {d}"),this);
    connect(deleteTetramerAct, SIGNAL(triggered()), this, SLOT(deleteTetramer()));

    saveTetramerAct = new QAction(tr("Save {s}"),this);
    connect(saveTetramerAct, SIGNAL(triggered()), this, SLOT(saveTetramer()));

    changePlacementAct = new QAction(tr("Change Placement {t}"), this);
    connect(changePlacementAct, SIGNAL(triggered()), this , SLOT(showPlacement()));

    hideEMImageAct = new QAction(tr("Hide EM Image {h}"), this);
```

```
connect(hideEMImageAct, SIGNAL(triggered()), this , SLOT(hideEMImage()));

showTetramerExtentAct = new QAction(tr("Show/Hide Alpha Shape & Hull{r}"),this);
connect(showTetramerExtentAct, SIGNAL(triggered()), this, SLOT(showExtent()));

showNearestNeighboursAct = new QAction(tr("Show Nearest Neighbours {n}"),this);
connect(showNearestNeighboursAct, SIGNAL(triggered()), this, SLOT(showNearestNeighbours()));

showRedTetramersAct = new QAction(tr("Red Tetramers {F1}"),this);
connect(showRedTetramersAct, SIGNAL(triggered()), this, SLOT(showRedTetramers()));

showTetramersAct = new QAction(tr("Hide Tetramers {F2}"),this);
connect(showTetramersAct, SIGNAL(triggered()), this, SLOT(hideTetramers()));

changeShadowAct = new QAction(tr("Circular Shadow {F3}"),this);
connect(changeShadowAct, SIGNAL(triggered()), this, SLOT(changeShadowOutline()));

toggleShadowAct = new QAction(tr("Show Tetramer Shadow {F4}"),this);
connect(toggleShadowAct, SIGNAL(triggered()), this, SLOT(toggleShadow()));

DrawContourAct = new QAction(tr("Outline Region {F5}"), this);
connect(DrawContourAct, SIGNAL(triggered()), this, SLOT(DrawContour()));

ScaleBarAct = new QAction(tr("Display Scale Bar (size in nm) {F6}"), this);
connect(ScaleBarAct, SIGNAL(triggered()), this, SLOT(getScaleBarSize()));

FloodFillTypeAct = new QAction(tr("FloodFill = Surround {F7}"),this);
connect(FloodFillTypeAct, SIGNAL(triggered()), this, SLOT(setFloodFillType()));

FloodAct = new QAction(tr("Isolate Region {F9}"),this);
connect(FloodAct, SIGNAL(triggered()), this, SLOT(ComputeFloodfill()));

ChangeTetramerWidthAct = new QAction(tr("Change Tetramer Width {F11}"), this);
connect(ChangeTetramerWidthAct, SIGNAL(triggered()), this, SLOT(ChangeTetramerWidth()));

FlipImageAct = new QAction(tr("Flip Image {F12}"), this);
connect(FlipImageAct, SIGNAL(triggered()), this, SLOT(FlipImage()));

hideAct = new QAction(tr("Hide/Unhide Frame No. {q}"),this);
connect(hideAct, SIGNAL(triggered()), this, SLOT(HideZ()));

autoAct  = new QAction(tr("   Autoscale"),this);
connect(autoAct, SIGNAL(triggered()), this, SLOT(AutoScale()));
autoAct->setCheckable(true);

fixedAct = new QAction(tr("   Fixed Scale"),this);
connect(fixedAct, SIGNAL(triggered()), this, SLOT(FixedScale()));
fixedAct->setCheckable(true);

if(nNoValidChannels == 1)
{
   colbwAct = new QAction(tr("B/W"),this);
   connect(colbwAct, SIGNAL(triggered()), this, SLOT(colourMono()));

   colredAct = new QAction(tr("Red"),this);
   connect(colredAct, SIGNAL(triggered()), this, SLOT(colourRed()));

   colgreenAct = new QAction(tr("Green"),this);
   connect(colgreenAct, SIGNAL(triggered()), this, SLOT(colourGreen()));

   colblueAct = new QAction(tr("Blue"),this);
   connect(colblueAct, SIGNAL(triggered()), this, SLOT(colourBlue()));
```

```
        colwbAct = new QAction(tr("W/B"),this);
        connect(colwbAct, SIGNAL(triggered()), this, SLOT(colourInvMono()));

        colLUTAct = new QAction(tr("LUT"),this);
        connect(colLUTAct, SIGNAL(triggered()), this, SLOT(colourLUT()));
    }

    changeLookAheadAct = new QAction(tr("Minimum or Sum Display {k}"), this);
    connect(changeLookAheadAct, SIGNAL(triggered()), this, SLOT(changeLookAhead()));

    LineAct = new QAction(tr("Measure Distance {l}"),this);
    connect(LineAct, SIGNAL(triggered()), this, SLOT(imageLine()));

    ZoomInAct = new QAction(tr("Zoom In {+}"),this);
    connect(ZoomInAct, SIGNAL(triggered()), this, SLOT(imageZoomIn()));

    ZoomOutAct = new QAction(tr("Zoom Out {-}"),this);
    connect(ZoomOutAct, SIGNAL(triggered()), this, SLOT(imageZoomOut()));

    HelpAct = new QAction(tr("Instructions {F10}"),this);
    connect(HelpAct, SIGNAL(triggered()), this, SLOT(Instructions()));

    QuitAct = new QAction(tr("Quit {esc}"),this);
    connect(QuitAct, SIGNAL(triggered()), this, SLOT(close()));

    if(nNoValidChannels == 1)
    {
        colourGroup = new QActionGroup(this);
        colourGroup->addAction(colbwAct);
        colourGroup->addAction(colredAct);
        colourGroup->addAction(colgreenAct);
        colourGroup->addAction(colblueAct);
        colourGroup->addAction(colwbAct);
        colourGroup->addAction(colLUTAct);
    }

    scaleGroup = new QActionGroup(this);
    scaleGroup->addAction(autoAct);
    scaleGroup->addAction(fixedAct);
    autoAct->setChecked(true);

}
bool RyRFit::CheckDimAndType(int nStackNo)
{
    // Check that TIFF images in a stack are consistent - i.e. same size and type

    int nSt = nStackNo - 1;

    int nXdim0 = 0;
    int nYdim0 = 0;
    int nInputType0 = -1;
    int nXdim1 = 0;
    int nYdim1 = 0;
    int nInputType1 = -1;
    char fn[512];
    for(int i=0; i < FileName[nSt].size(); i++)
    {
        QString fname=FileName[nSt].at(i);
        QFileInfo fi(fname);
        if(fname != QString("-"))
        {
            if(fi.suffix().isEmpty())
                FileName[nSt].replace(i, fname + QString(".tif"));
        }
        strcpy(fn, qPrintable(FileName[nSt].at(i)));
        TiffImage* imfile = new TiffImage;
        if(!imfile->ReadTiff(fn,' ',true))
```

```
        {
            cerr << "\n Error while reading in file parameters for " << fn << endl;
            delete imfile;
            return false;
        }
        nXdim1 = imfile->Xdim();
        nYdim1 = imfile->Ydim();
        nInputType1=imfile->InputType();
        nSZdim[nSt] += imfile->Zdim();
        if(i == 0)
        {
            QString DateandTime=imfile->DateTime();
            if(!DateandTime.isEmpty())
            {
                QString Year=DateandTime.mid(0,4);
                QString Month=DateandTime.mid(5,2);
                QString Day=DateandTime.mid(8,2);
                QString Time=DateandTime.mid(11,8);
                iminfo.DateandTime[nSt]= "Date: " + Day + '/' + Month + '/' + Year + " - Time: " + Time;
            }


            iminfo.ImageType[nSt] = QString().setNum(imfile->BitsPerSample()) + " bits/pixel";
            switch (imfile->Photometric())
            {
            case 0:
                iminfo.ImageType[nSt] += " grayscale image where White is minimum";
                break;
            case 1:
                iminfo.ImageType[nSt] += " grayscale image where Black is minimum";
                break;
            case 2:
                iminfo.ImageType[nSt] +=  " RGB image";
                break;
            case 3:
                iminfo.ImageType[nSt] +=  " palette colour image";
                break;
            case 4:
                iminfo.ImageType[nSt] +=  " transparency mask";
                break;
            }
            iminfo.Description[nSt] = imfile->ImageDescription();


            nX0 = imfile->X0();
            nY0 = imfile->Y0();
        }
        delete imfile;
        if(i > 0)
        {
            if(nXdim0 != nXdim1 || nYdim0 != nYdim1)
            {
                cerr << "\n Error - X & Y dimensions are different - files " << (i-1);
                cerr << " and " << i << " of stack " << nStackNo << "\n";
                return false;
            }
            if(nInputType0 != nInputType1)
            {
                cerr << "\n Error - Input types are different - files " << (i-1);
                cerr << " and " << i << " of stack " << nStackNo << "\n";
                return false;
            }
        }
        nXdim0 = nXdim1;
        nYdim0 = nYdim1;
        nInputType0 = nInputType1;
    }
    nSXdim[nSt] = nXdim0;
    nSYdim[nSt] = nYdim0;
    nSInputType[nSt] = nInputType0;


    return true;
}
void RyRFit::InputTiffMono(TiffImage* Tiff_Image, int nChannel)
```

```
{
// input Monochrome TIFF according to parameters stored
// Can also input single channel (R, G or B)

    int nDataPoints=Tiff_Image->PntsPerImage();
    uint16* ps1 = psDst[nChannel];

    bool bMinisWhite = Tiff_Image->WhiteisMin();

    if(Tiff_Image->BitsPerSample() == 8)
    {

// 8 bit Mono
        uint8 *pb = Tiff_Image->BytePointer();
        for(int k = 0; k < nDataPoints; k++)
        {
            if(bMinisWhite)
                *ps1++ = 255 - *pb++; // White is Min black is max
            else
                *ps1++ = *pb++;
        }
    }
    else
    {
// 16 bit Mono
        uint16 *ps = Tiff_Image->UShortPointer();
        for(int k = 0; k < nDataPoints; k++)
        {
            if(bMinisWhite)
                *ps1++ = 65535 - *ps++; // White is Min black is max
            else
                *ps1++ = *ps++;
        }
    }
    psDst[nChannel] = ps1;
}
void RyRFit::InputTiffRGB(TiffImage* Tiff_Image)
{
// input RGB TIFF according to parameters read

    int nDataPoints=Tiff_Image->PntsPerImage();

    uint16* ps1 = psDst[0];
    uint16* ps2 = psDst[1];
    uint16* ps3 = psDst[2];

    if(Tiff_Image->BitsPerSample() == 8)
    {
// 8 bit RGB
        uint8 *pb = Tiff_Image->BytePointer();

        for(int k = 0; k < nDataPoints; k++)
        {
            *ps1++ = *pb++;
            *ps2++ = *pb++;
            *ps3++ = *pb++;
        }
    }
    else
    {
//  16 bit RGB
        uint16 *ps = Tiff_Image->UShortPointer();

        for(int k = 0; k < nDataPoints; k++)
        {
            *ps1++ = *ps++;
            *ps2++ = *ps++;
            *ps3++ = *ps++;
```

```
        }
    }
    psDst[0] = ps1;
    psDst[1] = ps2;
    psDst[2] = ps3;
}
bool RyRFit::InputTiffMap(TiffImage* Tiff_Image)
{
// input a Mapped TIFF according to parameters read
    int nDataPoints=Tiff_Image->PntsPerImage();


    uint16* ps1 = psDst[0];
    uint16* ps2 = psDst[1];
    uint16* ps3 = psDst[2];


    uint16* r;
    uint16* g;
    uint16* b;
    Tiff_Image->ColourMap(r, g, b); // get colour map
    int num_entries = 1 << Tiff_Image->BitsPerSample() ;


    if(Tiff_Image->BitsPerSample() == 8)
    {
 //  8 bit Map
        uint8 *pb = Tiff_Image->BytePointer();


        for(int k = 0; k < nDataPoints; k++)
        {
            if(*pb >= num_entries)
            {
                cerr << " Error - value of " << *pb << " exceeds number of entries in lookup table = " << num_entries << endl;
                return false;
            }
            *ps1++ = *(r + *pb);
            *ps2++ = *(g + *pb);
            *ps3++ = *(b + *pb);
            pb++;
        }
    }
    else
    {
        uint16 *ps = Tiff_Image->UShortPointer();
// 16 bit Map


        for(int k = 0; k < nDataPoints; k++)
        {
            if(*ps >= num_entries)
            {
                cerr << " Error - value of " << *ps << " exceeds number of entries in lookup table = " << num_entries << endl;
                return false;
            }
            *ps1++ = *(r + *ps);
            *ps2++ = *(g + *ps);
            *ps3++ = *(b + *ps);
            ps++;
        }
    }


    psDst[0] = ps1;
    psDst[1] = ps2;
    psDst[2] = ps3;


    return true;
}
bool RyRFit::CheckChannels()
{
// Check Channels for valid values - i.e. non-zero good stats.
    for(int n = 0; n < nNoChannels; n++)
    {
        uint16* ps = psD[n];
```

```
        nFmin[n] = 66000;
        nFmax[n] = 0;

        for(int k = 0; k < nPixelsPerPlane*nZPlanes; k++)
        {
            nFmin[n] = (nFmin[n] < *ps)?  nFmin[n] : *ps;
            nFmax[n] = (nFmax[n] > *ps)?  nFmax[n] : *ps;
            ps++;
        }

        if(nFmax[n] > nFmin[n])
        {

            nValidChannel[nNoValidChannels++] = n;

            nPMaxpos[n] = new int [nZPlanes];
            nPMinpos[n] = new int [nZPlanes];
            nPpts[n] = new int [nZPlanes];
            nPixelsPerPlane = nXdim*nYdim;
            sPmin[n] = new int[nZPlanes];
            sPmax[n] = new int[nZPlanes];

            if(CalculateStatistics(n) > 0)return false;
            nThreshold[n] = nFmin[n];
            // create space for lookup table
            lookup[n] = new GLubyte [nFmax[n]-nFmin[n] + 1];
        }
    }
    return true;
}
int RyRFit::CalculateStatistics(int imageno)
{
// calculate minimum and maximum values for each channel (R,G or B or monochrome = single channel)
// this is used for scaling the images
    nFmin[imageno] = USHRT_MAX;
    nFmax[imageno] = 0;

    uint16* ps=psD[imageno];
    if(ps == NULL)exit(1);

    for (int k = 0; k < nZPlanes; k++)
    {
        int Minval= USHRT_MAX;
        int Maxval= 0;
        int npts = 0;
        for( int i=0; i < nPixelsPerPlane; i++)
        {
            if(*ps > Maxval)
            {
                Maxval=*ps;
                nPMaxpos[imageno][k] = i;
                if(Maxval > nFmax[imageno])
                    nFmax[imageno] = Maxval;
            }
            if(*ps < Minval)
            {
                Minval=*ps;
                nPMinpos[imageno][k] = i;
                if(Minval < nFmin[imageno])
                    nFmin[imageno] = Minval;
            }
            npts++;
            ps++;
        }
        nPpts[imageno][k] = npts;
        sPmin[imageno][k] = Minval;
        sPmax[imageno][k] = Maxval;
    }
    if(nFmax[imageno] <= nFmin[imageno])
    {
```

```
        QMessageBox::warning(this,"RyR_Fit",
                             "Error - Invalid values in TIFF data!!",
                             QMessageBox::Ok,QMessageBox::NoButton);
        return 100;
    }
    if(bScale[imageno])
    {
        nSmin[imageno] = qMax(nFmin[imageno],nBlackLevel[imageno]);
        nSmax[imageno] = qMin(nFmax[imageno],
                              int(248*fScale[imageno]+nSmin[imageno]));
    }
    else
    {
        nSmin[imageno] = nFmin[imageno];
        nSmax[imageno] = nFmax[imageno];
    }
    return 0;
}
bool RyRFit::ReadTiffStack(int stackno)
{
// read in a TIFF stack either from a single stack or multiple images
    char fn[512];
    cerr << "\n Stack " << (stackno+1) << " - reading : ";
    for(int m = 0; m < FileName[stackno].size(); m++)
    {
        strcpy(fn, qPrintable(FileName[stackno].at(m)));
        TiffImage*  Tiff_Image = new TiffImage;
        if(!Tiff_Image->ReadTiff(fn))
        {
            cerr << "\n Error while reading in file " << fn << endl;
            delete Tiff_Image;
            return false;
        }


        if(Tiff_Image->InputType() == TIFF_Mono_8bit ||
                Tiff_Image->InputType() == TIFF_Mono_16bit)
            InputTiffMono(Tiff_Image,stackno);


        else if(Tiff_Image->InputType() == TIFF_RGB_8bit ||
                Tiff_Image->InputType() == TIFF_RGB_16bit)
            InputTiffRGB(Tiff_Image);


        else
            InputTiffMap(Tiff_Image);


        delete Tiff_Image;
    }
    cerr << nSZdim[stackno] << " planes";
    return true;
}
```

**** ryr_fit3a.h ****

```
/*******************************************************************************************
* Header file for ryr_fit3a.cpp that reads allows placement of tetramers on Amira-generated
* TIFF images
*
* Copyright David Scriven, 2012-2019.
*
* Moore Laboratory, Life Sciences Institute, 2350 Health Sciences
* Mall, University of British Columbia, Vancouver, Canada, V6T 1Z3
*
* This file, ryr_fit3a.h is part of the RYR_FIT program
*
* This program is free software: you can redistribute it and/or modify
* it under the terms of the GNU General Public License as published by
* the Free Software Foundation, either version 3 of the License, or
* any later version.
*******************************************************************************************/
#ifndef _ryrfit_h
#define _ryrfit_h


#include <QGLWidget>
#include <QString>
#include <QContextMenuEvent>
#include <QPoint>
#include <QLine>
#include <QStringList>
#include <QColor>
#include <QVector>
#include <vector>
#include "tiff.h"
#include "imstruct.h"
#include <CGAL/Exact_predicates_inexact_constructions_kernel.h>
typedef CGAL::Exact_predicates_inexact_constructions_kernel G;
typedef G::Segment_2 Segment;
typedef G::Point_2  APoint;


class QAction;
class QActionGroup;
class QFont;
class QMenu;
class QCursor;
class TiffImage;
class ScaleWnd;
class QScrollArea;
class Placement;


struct Tetramer
{
  QPoint centre;
  int angle;
  char classification;
};
struct NewTetramer
{
    double xpos;
    double ypos;
    bool bStipple;
};


#define TPERPNTS 8192


class RyRFit : public QGLWidget
{
    Q_OBJECT

public:
    RyRFit();
    ~RyRFit();
    bool ParseCmdString(int argc, char** argv);
```

```
    int  ShowImage();
    int  NoChannels() {return nNoChannels;}


public slots:
    void ChangeScale(int* nLow, int* nHi);
    void ChangeTetramerWidth();
    void setupSingleTetramer();
    void setupLaiSidebySide();
    void setupCheckerboard(double tetrameroverlap);
    void setupLaiChckrbrd();
protected:
    enum {RED, YELLOW, GREEN, CYAN, BLUE, MAGENTA, MONO, INVMONO,
          LUT, PSEUDOCOLOUR};
    enum {SQUARE, CIRCLE};
    void contextMenuEvent(QContextMenuEvent *event);
    void keyPressEvent( QKeyEvent *e );
    void keyReleaseEvent( QKeyEvent *e );
    void mousePressEvent( QMouseEvent *e );
    void mouseReleaseEvent( QMouseEvent *e );
    void mouseMoveEvent( QMouseEvent *e );
    void moveEvent(QMoveEvent *e);
    void wheelEvent(QWheelEvent *event);
    void closeEvent(QCloseEvent* e);
    void resizeEvent(QResizeEvent* e);
    void createActions();
    void createMenus();
    void CreateStack();
    void paintGL();
    void initializeGL();
    void resizeGL(int x, int y);
    void LookAhead(int n);
    void imageDisplay();
    void drawTetramer(QPoint posn, int angle, char classification = 'u');
    void drawCheckerboard(QPoint posn, int angle);
    void drawLaiSidebySide(QPoint posn, int angle);
    void findTetramer(QPoint posn);
    void tetramerClassification(char c);


    void calculateArea();
    void PlotPerimeters();
    void calculateNearestNeighbours();
    char setType(int nPlacementType);
//    void sideView();
    int  GetData(TiffImage* image, QString filename, int imageno);
    int  CalculateStatistics(int imageno);

    uint16 PixelVal(uint16* ps, QPoint point);


private slots:
    void AutoScale();
    void FixedScale();
    void toggleScale(bool bState);


    void addTetramer();
    void saveTetramer();
    void deleteTetramer();
    void modifyTetramer();
    void classifyTetramer();
    void showExtent();
    void showNearestNeighbours();
    void showPlacement();


    void changeLookAhead();
    void showRedTetramers();
    void hideTetramers();
    void changeShadowOutline();
    void setFloodFillType();
    void DrawContour();
    void ComputeFloodfill();
    void hideEMImage();
    void toggleShadow();
```

```
    void FlipImage();
    void Instructions();

    void HideZ();

    void colourMono();
    void colourRed();
    void colourGreen();
    void colourBlue();
    void colourInvMono();
    void colourLUT();
    void CreateColourMap(int nColourDisplay);

    void getScaleBarSize();

    void imageLine();
    void imageFirstPlane();
    void imageLastPlane();
    void imageNextPlane();
    void imagePrevPlane();
    void imageZoomIn();
    void imageZoomOut();

    void ChangeSize();
    void CloseProgram();

    void CalculateBitmap();

protected:
    void FitShape(std::vector<APoint> PixelGroup);
    bool CheckDimAndType(int nStackNo);
    bool CheckChannels();
    bool ReadTiffStack(int nStackNo);
    void InputTiffMono(TiffImage* Tiff_Image, int nChannel);
    void InputTiffRGB(TiffImage* Tiff_Image);
    bool InputTiffMap(TiffImage* Tiff_Image);
    bool DoFloodFill(int* plane);

    void LineDraw();

    QPoint LimitMouse(int x, int y);
    QPoint RealPos(QPoint pos);
    QVector &lt;Segment&gt; segments;
    QVector &lt;Segment&gt; cvexhull;

private:
// menus
    QMenu* imageMenu;
    QMenu* tetramerMenu;
    QMenu* bscaleMenu;
    QMenu* iscaleMenu;
    QMenu* colourMenu;

    QActionGroup* colourGroup;
    QActionGroup* scaleGroup;

    QAction* autoAct;
    QAction* fixedAct;
    QAction* hideAct;

    QAction* addTetramerAct;
    QAction* deleteTetramerAct;
    QAction* modifyTetramerAct;
    QAction* showTetramersAct;
    QAction* FloodFillTypeAct;
    QAction* FlipImageAct;
    QAction* toggleShadowAct;
```

```
    QAction* saveTetramerAct;
    QAction* showTetramerExtentAct;
    QAction* showNearestNeighboursAct;
    QAction* changePlacementAct;
    QAction* changeLookAheadAct;
    QAction* showRedTetramersAct;
    QAction* hideEMImageAct;
    QAction* FloodAct;
    QAction* DrawContourAct;
    QAction* changeShadowAct;
    QAction* ChangeTetramerWidthAct;
    QAction* ScaleBarAct;
    QAction* HelpAct;


    QAction* colbwAct;
    QAction* colredAct;
    QAction* colgreenAct;
    QAction* colblueAct;
    QAction* colwbAct;
    QAction* colLUTAct;


    QAction* LineAct;
    QAction* ZoomInAct;
    QAction* ZoomOutAct;
    QAction* QuitAct;

    QFont*   sysfont;
    QString  infoLabel;
    QString  fontcolour[3];

    QScrollArea* imageScroll;
    QCursor* Cursor;


protected:
    uint16*   psD[3];
    uint16*   psDst[3];


    ScaleWnd* ScaleVal;
    Placement* TetramerPlacement;


    Imageinfo  iminfo;


    int nNoTetramers;
    int nAddedTetramers;
    Tetramer tm[100];
    NewTetramer ntm[9];


    int nPerPnts;
    QPoint   PerPnt[TPERPNTS];
    QPoint   previous;
    QPoint   lastMousePosn;


    bool    bDrawing;


    QLine  nnLine[100];
    QColor linecolor[8];
    QPoint TetramerPosn;
    int TetramerAngle;
    int nBeingModified;
    int nBeingClassified;
    int ShadowShape;
    QRect    FullArea;
    float mnnd[100];


    QStringList  FileName[3];
    QString  DataFileName;
    QString  NNFileName;
```

```
    int   nWinXPos;
    int   nWinYPos;

    GLubyte redlut[256];
    GLubyte greenlut[256];
    GLubyte bluelut[256];

    char    cSumtype;

    bool    bShowZ;
    bool    bBorder;
    bool    bScaleBar;
    bool    bSpecial;
    bool    bRGB;
    bool    bSave;
    bool    bEnteredScale;
    bool    bLookAhead;
    bool    bAltPressed;
    bool    bPickedCentre;
    bool    bSingleColor;
    bool    bFlood;
    bool    bFloodCalculated;
    bool    bPartialFlood;
    bool    bShowShadow;
    bool    bShowTetramers;
    bool    bFlip;
    bool    bAnalyseOnly;

    bool    bLeftButtonDown;
    bool    bRightButtonDown;
    bool    bMiddleButtonDown;

    bool    bLinePlot;
    bool    bAddTetramer;
    bool    bModifyTetramer;
    bool    bClassifyTetramer;
    bool    bPlacedTetramer;
    bool    bHideEMImage;
    bool    bShowArea;
    bool    bDisplayNND;

    bool    bBitmap;

    int     nZoom;
    int     nOldZoom;
    int     windowY;
    QPoint  currentPos;
    QPoint  mousePos;

//dealing with ROI

    QPoint   StartMove;
    QPoint   StartPt;
    QPoint   EndPt;

    int nInitialColour;

    GLubyte* planeimage;
    GLubyte* imageseg;
    GLubyte** lookup;
    bool* hilite;

// Displayable min & max & Z planes

    int cMax;
    int cMin;
```

```
int nZmin;
int nZmax;
int nLookAhead;

int nNoStacks;
int nNoChannels;
int nNoValidChannels;
int nXdim;
int nYdim;
int nBitmapXdim;
int nBitmapYdim;

int nSXdim[3];
int nSYdim[3];
int nSZdim[3];
int nSInputType[3];
int nValidChannel[3];

int nPixelsPerPlane;
int nX0;
int nY0;

QString Description;
QString Software;

int nFmin[3];
int nFmax[3];
int nThreshold[3];

int nImageXdim;
int nImageYdim;

int nPlaneNo;
int nZPlanes;

int nXScreenMax;
int nYScreenMax;

int nScaleBarLength;

int nPlacementType;
double degtorad;
double fortyfivedeg;
double tetramerdiag;
double tetramerwidth;
double tetramersize;
double laicheckerboarddiag;
double laisidebysidediag;
double baseangleinradians;
double centredistance;
double sqrt2;
double AreaConvex;
double AreaAlpha;

double totalConvexArea;
double totalAlphaArea;
double totalTetramerArea;
double percentTetramerAreaConvex;
double percentTetramerAreaAlpha;
double TetramerDensityConvex;
double TetramerDensityAlpha;

float nndmax;
float nndmin;

float fMicronsPerPixel;
float fnmPerPixel;
```

```
    float fXPixelSize;
    float fYPixelSize;

    bool bScale[3];
    float fScale[3];
    int nBlackLevel[3];


    int TimerInterval;


//Statistics  data
    int** sPmin;
    int** sPmax;
    int** nPMaxpos;
    int** nPMinpos;
    int** nPpts;


    static bool PixelPosnLessThan(const APoint &s1, const APoint &s2)
    {
        return (s1.x() < s2.x());
    }


    static bool bAutoScale;
    static int nSmax[3];
    static int nSmin[3];
};
#endif
```

**** scale.cpp ****

```
/******************************************************************************************
* Helper file for RYR_FIT that allows scaling all channels of a displayed TIFF file
*
* Copyright David Scriven, 2012-2019.
*
* Moore Laboratory, Life Sciences Institute,  2350 Health Sciences
* Mall, University of British Columbia, Vancouver, Canada, V6T 1Z3
*
* This file, scale.cpp is part of the RYR_FIT program
*
* RYR_FIT links to the proprietary Qt system (ver 4.7) as well as the the free
* CGAL algorithmic library and the free TIFF library.
*
* This program is free software: you can redistribute it and/or modify
* it under the terms of the GNU General Public License as published by
* the Free Software Foundation, either version 3 of the License, or
* any later version.
*
* This program is distributed in the hope that it will be useful,
* but WITHOUT ANY WARRANTY; without even the implied warranty of
* MERCHANTABILITY or FITNESS FOR A PARTICULAR PURPOSE.  See the
* GNU General Public License for more details.
*
* You should have received a copy of the GNU General Public License
* along with this program.  If not, see <https://www.gnu.org/licenses/>.
*******************************************************************************************/


#include <QGLWidget>
#include "scale.h"


using namespace std;


ScaleWnd::ScaleWnd(QGLWidget *parent, Imageinfo iminfo, int* _nFmin, int* _nFmax,
                   GLubyte* r, GLubyte* g, GLubyte* b) : QGLWidget(nullptr, parent)
{
    bLeftButtonDown = false;
    nNoValidChannels = iminfo.nNoValidChannels;
    bRGB = iminfo.bRGB;

    for(int i = 0; i < 3; i++)
    {
        nFmin[i] = _nFmin[i]; //absolute limits
        nFmax[i] = _nFmax[i];
        nSmin[i] = _nFmin[i]; // set limits
        nSmax[i] = _nFmax[i];
        nValidChannel[i] = iminfo.nValidChannel[i];
        fRange[i] = float(nFmax[i]-nFmin[i]);
    }
    nSpacing = 45;

    scalemap = nullptr;
    scalemap = new GLubyte[20832];  // 28 * 248 *3

    redlut = r;
    greenlut = g;
    bluelut = b;
    GLubyte *sc = scalemap;
    for(int j = 0; j < 248; j++)
    {
        GLubyte val = GLubyte(j);
        for (int k = 0; k < 28; k++)
        {
            *sc++ = redlut[val];
            *sc++ = greenlut[val];
            *sc++ = bluelut[val];
        }
    }

    nCanvasWidth = 75;
```

```
    nCanvasWidth = nNoValidChannels*nSpacing + 10;
    if(nNoValidChannels == 1)nCanvasWidth += 5;
    nCanvasHeight = 320;
    boxbot = 30;
    boxtop = boxbot + 248;

    xposmin = 17;
    xposmax = 47;

    sysfont = new QFont("Helvetica");
    sysfont->setPointSize(13);

    setMinimumSize(nCanvasWidth, nCanvasHeight);
    resize(nCanvasWidth, nCanvasHeight);
    setWindowTitle("Scale");

    connect(this,SIGNAL(RangeChanged(int*, int*)),parent,SLOT(ChangeScale(int*, int*)));
    connect(this,SIGNAL(CloseProgram()),parent,SLOT(CloseProgram()));
    connect(this,SIGNAL(ScaleOff()),parent,SLOT(AutoScale()));
}
ScaleWnd::~ScaleWnd()
{
    if(isVisible())hide();
    delete [] scalemap;
}
void ScaleWnd::keyPressEvent(QKeyEvent* e)
{
    switch(e->key())
    {
    case Qt::Key_Escape:
        emit CloseProgram();
        break;
    case Qt::Key_Control:
        emit ScaleOff();
        break;
    default:
        QWidget::keyPressEvent(e);
        break;
    }
}
void ScaleWnd::mouseReleaseEvent(QMouseEvent* e)
{
    if(e->button() == Qt::LeftButton)
    {
        bMinBox[0] = bMinBox[1] = bMinBox[2] = false;
        bMaxBox[0] = bMaxBox[1] = bMaxBox[2] = false;
        bLeftButtonDown = false;
    }
    else
        QWidget::mouseReleaseEvent(e);
}
void ScaleWnd::mousePressEvent(QMouseEvent* e)
{
    if(e->button() == Qt::LeftButton)
    {
        QPoint mousePosn(e->x(),nCanvasHeight-e->y());
        bLeftButtonDown=true;
        int i = CheckXpos(mousePosn);
        if(i == -1)return;
        if(mousePosn.x() < xposmin && mousePosn.x() > xposmax)return;
        bMinBox[i] = (mousePosn.y() > minpos[i] - 9) && (mousePosn.y() < minpos[i]) ;
        bMaxBox[i] = (mousePosn.y() < maxpos[i] + 9) && (mousePosn.y() > maxpos[i]) ;
        //    cerr << bMinBox[i] << " " << bMinBox[i] << "\n";
        StartPt = mousePosn;
    }
    else
        QWidget::mousePressEvent(e);
}
int ScaleWnd::CheckXpos(QPoint point)
{
    int i = -1;
    for(int j = 0; j < nNoValidChannels; j++)
    {
```

```
        int xposmin = 10 + j*nSpacing;
        int xposmax = 40 + j*nSpacing;
        if(nNoValidChannels == 1)
        {
            xposmin += 7;
            xposmax += 7;
        }
        if(point.x() > xposmin && point.x() < xposmax)i=j;
    }
    return i;
}
void ScaleWnd::mouseMoveEvent(QMouseEvent* e)
{
    if(!bLeftButtonDown)return;
    QPoint point(e->x(), nCanvasHeight-e->y());
    //   cerr << point.x << "," << point.y << "\n";
    if(point.y() < 3 || point.y() > (nCanvasHeight -3))return;
    int i = CheckXpos(point);
    if(i == -1) return;
    int nYmove = point.y() - StartPt.y();
    if(nYmove == 0) return;
    if(bMinBox[i])
        minpos[i]=qMax(boxbot, qMin(maxpos[i]-1, minpos[i]+nYmove));
    if(bMaxBox[i])
        maxpos[i]=qMin(boxtop, qMax(minpos[i]+1, maxpos[i]+nYmove));

    int j = nValidChannel[i];
    nSmin[j] = int(float(minpos[i] - boxbot)/248.0*fRange[j])+ nFmin[j];
    nSmax[j] = int(float(maxpos[i] - boxbot)/248.0*fRange[j])+ nFmin[j];
    emit RangeChanged(nSmin, nSmax);
    StartPt = point;
    updateGL();
}
void ScaleWnd::initializeGL()
{
    // Setup OpenGl
    glDisable(GL_BLEND);
    glDisable(GL_ALPHA_TEST);
    glDisable(GL_DITHER);
    glDisable(GL_DEPTH_TEST);
    glDisable(GL_FOG);
    glDisable(GL_LIGHTING);
    glDisable(GL_LINE_STIPPLE);
    glDisable(GL_LOGIC_OP);
    glDisable(GL_STENCIL_TEST);
    glDisable(GL_TEXTURE_1D);
    glDisable(GL_TEXTURE_2D);
    glShadeModel(GL_FLAT);

    glMatrixMode(GL_PROJECTION);
    glLoadIdentity();
    glOrtho(0, nCanvasWidth, 0, nCanvasHeight, 0.0, 1.0);
    glMatrixMode(GL_MODELVIEW);
    glLoadIdentity();
    glViewport(0, 0, nCanvasWidth, nCanvasHeight);
}
void ScaleWnd::paintGL()
{
    qglClearColor(Qt::white);
    glClear(GL_COLOR_BUFFER_BIT);
    glRasterPos2i(0,0);

    bool bMultiChannel = (bRGB || nNoValidChannels > 1);

    QColor maxpen = Qt::red;     // single channel defns
    QColor minpen = Qt::blue;

    for(int i = 0; i < nNoValidChannels; i++)
    {
        GLubyte *sc = scalemap;
        memset(sc, 0, 20832);
        int offset;
        int nV = nValidChannel[i];
```

```
if(bMultiChannel)
{
    offset = i*nSpacing;
    switch (nV)
    {
    case 0:
        minpen=maxpen=Qt::red;
        break;
    case 1:
        sc += 1;
        minpen=maxpen=Qt::green;
        break;
    case 2:
        sc += 2;
        minpen=maxpen=Qt::blue;
        break;
    }
}
else
    offset = 5;

GLubyte val = 0;
int bval = 28*(minpos[i]-boxbot);
for(int jb = 0; jb < bval ; jb++)
{
    if(bMultiChannel)
    {
        *sc = val;
        sc += 3;
    }
    else
    {
        *sc++ = redlut[val];
        *sc++ = greenlut[val];
        *sc++ = bluelut[val];
    }
}
float fDel = 248.0/float(maxpos[i]-minpos[i]);
int nR = maxpos[i]-minpos[i];
for(int j = 0; j < nR; j++)
{
    val = GLubyte(j*fDel);
    for (int k = 0; k < 28; k++)
    {
        if(bMultiChannel)
        {
            *sc = val;
            sc += 3;
        }
        else
        {
            *sc++ = redlut[val];
            *sc++ = greenlut[val];
            *sc++ = bluelut[val];
        }
    }
}
int tval = 28*248;
int sval = 28*(maxpos[i]-boxbot);
val = GLubyte(248);
for(int jt = sval; jt < tval; jt++)
{
    if(bMultiChannel)
    {
        *sc = val;
        sc += 3;
    }
    else
    {
        *sc++ = redlut[val];
        *sc++ = greenlut[val];
        *sc++ = bluelut[val];
```

```
            }
        }

        qglColor(maxpen);
        glBegin(GL_LINE_LOOP);   // box around bitmap
        glVertex2i(10 + offset,boxbot);
        glVertex2i(10 + offset,boxtop);
        glVertex2i(40 + offset,boxtop);
        glVertex2i(40 + offset,boxbot);
        glEnd();

        glRasterPos2i(12 + offset ,boxbot);
        glDrawPixels(27,248,GL_RGB,GL_UNSIGNED_BYTE, scalemap);

        glRasterPos2i(0,0);

        // min line & box
        qglColor(Qt::darkGray);
        glRecti(12 + offset ,minpos[i]- 8, 39 + offset,minpos[i]-1);
        glBegin(GL_LINES);
        glVertex2i(5 + offset,minpos[i]);
        glVertex2i(45 + offset,minpos[i]);
        glEnd();

        // max line & box
        glRecti(12 + offset,maxpos[i] + 2,39 + offset,maxpos[i]+ 9);
        glBegin(GL_LINES);
        glVertex2i(5 + offset,maxpos[i]);
        glVertex2i(45 + offset,maxpos[i]);
        glEnd();

        QString Value;
        qglColor(minpen);
        Value.sprintf("%d",nSmin[nV]);
        renderText(10 + offset, boxtop + 30 , Value);

        qglColor(maxpen);
        Value.sprintf("%d",nSmax[nV]);
        renderText(10 + offset, boxbot - 5, Value);
    }
}
void ScaleWnd::Show(int* Smin, int* Smax)
{
    for (int i = 0; i < nNoValidChannels; i++)
    {
        int j = nValidChannel[i];
        nSmin[j] = Smin[j];
        nSmax[j] = Smax[j];
        minpos[i] = int(248.0*float(nSmin[j]-nFmin[j])/fRange[j]) + boxbot;
        maxpos[i] = int(248.0*float(nSmax[j]-nFmin[j])/fRange[j]) + boxbot;
    }
    show();
}
void ScaleWnd::moveImage(int xparent, int yparent, int width)
{
    move(xparent + width, yparent);
}
void ScaleWnd::SetColorMap()
{
    makeCurrent();
    updateGL();
}
```

**** scale.h ****

```
/*****************************************************************************************
* Header file for scale.cpp that reads allows scaling of TIFF channels.
*
* Copyright David Scriven, 2012-2019.
*
* Moore Laboratory, Life Sciences Institute,  2350 Health Sciences
* Mall, University of British Columbia, Vancouver, Canada, V6T 1Z3
*
* This file, scale.h is part of the RYR_FIT program
*
* This program is free software: you can redistribute it and/or modify
* it under the terms of the GNU General Public License as published by
* the Free Software Foundation, either version 3 of the License, or
* any later version.
*****************************************************************************************/


#ifndef __scale_h
#define __scale_h


#include <QGLWidget>
#include <QContextMenuEvent>
#include "imstruct.h"


class QPoint;
class QFont;


class ScaleWnd : public QGLWidget
{
    Q_OBJECT


    void keyPressEvent( QKeyEvent *e );
    void mousePressEvent( QMouseEvent *e );
    void mouseReleaseEvent( QMouseEvent *e );
    void mouseMoveEvent( QMouseEvent *e );
    int  CheckXpos(QPoint point);


    void initializeGL();
    void paintGL();

public:
    ScaleWnd(QGLWidget* cw, Imageinfo iminfo, int* nFmin, int* nFmax, GLubyte* r, GLubyte* g, GLubyte* b);
    ~ScaleWnd();
    void Show(int* Smin, int* Smax);
    void moveImage(int x, int y, int width);
    void SetColorMap();
signals:
    void RangeChanged(int* nSmin, int* nSmax);
    void CloseProgram();
    void ScaleOff();
protected:
    QFont*  sysfont;
    GLubyte* scalemap;
    GLubyte* redlut;
    GLubyte* greenlut;
    GLubyte* bluelut;

    QPoint StartPt;


    bool bMinBox[3];
    bool bMaxBox[3];
    bool bLeftButtonDown;
    bool bRGB;


    float fRange[3];
```

```
    int nFmin[3];
    int nFmax[3];
    int maxpos[3];
    int minpos[3];
    int nSmin[3];
    int nSmax[3];
    int nValidChannel[3];

    int nCanvasWidth;
    int nCanvasHeight;
    int nSpacing;
    int boxbot;
    int boxtop;
    int xposmin;
    int xposmax;
    int nNoValidChannels;
};


#endif
```

**** placement.cpp ****

```
/****************************************************************************************
* Helper file for RYR_FIT that allows placement of different types of RyR tetramer groups
*
* Copyright David Scriven, 2012-2019.
*
* Moore Laboratory, Life Sciences Institute,  2350 Health Sciences
* Mall, University of British Columbia, Vancouver, Canada, V6T 1Z3
*
* This file, placement.cpp is part of the RYR_FIT program
*
* RYR_FIT links to the proprietary Qt system (ver 4.7) as well as the the free
* CGAL algorithmic library and the free TIFF library.
*
* This program is free software: you can redistribute it and/or modify
* it under the terms of the GNU General Public License as published by
* the Free Software Foundation, either version 3 of the License, or
* any later version.
*
* This program is distributed in the hope that it will be useful,
* but WITHOUT ANY WARRANTY; without even the implied warranty of
* MERCHANTABILITY or FITNESS FOR A PARTICULAR PURPOSE.  See the
* GNU General Public License for more details.
*
* You should have received a copy of the GNU General Public License
* along with this program.  If not, see <https://www.gnu.org/licenses/>.
****************************************************************************************/


#include "placement.h"
// This subroutine allows the user to place of
//  1. a single tetramer
//  2. a group of 5 tetramers that follows the Lai checkerboard arrangement
//  3. a group of 9 tetramers that follows the Lai side-by-side arrangement
//  4. a group of 5 tetramers that follows a checkerboard arrangement with a user-defined overlap (nm)
//
Placement::Placement()
{
  setupUi(this);
  connect(rbVariableOverlap, SIGNAL(toggled(bool)), this, SLOT(toggleOverlap(bool)));
  connect(bBExitCancel, SIGNAL(accepted()), this, SLOT(Exit()));
  connect(bBExitCancel, SIGNAL(rejected()), this, SLOT(Cancel()));
  leOverlap->hide();
  lbl_nm->hide();
  lbl_overlap->hide();
}
void Placement::Show(int placement)
{
    switch(placement)
    {
      case 1: rbSingleTetramer->setChecked(true); break;
      case 2: rbLaiCheckerboard->setChecked(true); break;
      case 3: rbLaiSidebySide->setChecked(true); break;
      case 4: rbVariableOverlap->setChecked(true); break;
    }
    show();
}


void Placement::toggleOverlap(bool bState)
{
   if(bState)
   {
     leOverlap->show();
     lbl_nm->show();
     lbl_overlap->show();
   }
   else
   {
     leOverlap->hide();
     lbl_nm->hide();
     lbl_overlap->hide();
   }
```

```
}
void Placement::Cancel()
{
  hide();
}
void Placement::Exit()
{
  if(rbSingleTetramer->isChecked())
  {
    SingleTetramer();
  }
  if(rbLaiCheckerboard->isChecked())
  {
    LaiCheckerboard();
  }
  if(rbLaiSidebySide->isChecked())
  {
    LaiSidebySide();
  }
  if(rbVariableOverlap->isChecked())
  {
    if(leOverlap->text().isEmpty())
      return;
    double overlap = leOverlap->text().toDouble();
    Checkerboard(overlap);
  }
  hide();
}
```

**** placement.h ****

```
/*****************************************************************************************
* Header file for placement.cpp that reads allows changing the types of tetramers placed
* on the TIFF images.
*
* Copyright David Scriven, 2012-2019.
*
* Moore Laboratory, Life Sciences Institute,  2350 Health Sciences
* Mall, University of British Columbia, Vancouver, Canada, V6T 1Z3
*
* This file, placement.h is part of the RYR_FIT program
*
* This program is free software: you can redistribute it and/or modify
* it under the terms of the GNU General Public License as published by
* the Free Software Foundation, either version 3 of the License, or
* any later version.
*****************************************************************************************/

#ifndef __placement_h
#define __placement_h

#include "ui_placement.h"

class Placement : public QDialog, private Ui::Placement
{
   Q_OBJECT
public:
   Placement();
   void Show(int PlacementType);
private slots:
   void toggleOverlap(bool bState);
   void Cancel();
   void Exit();

signals:
   void SingleTetramer();
   void LaiCheckerboard();
   void LaiSidebySide();
   void Checkerboard(double overlap);
};
#endif
```

****** tiff.cpp ******

```
/*****************************************************************************************
* Helper file for RYR_FIT that reads in various types of TIFF files - based on the
* free TIFF library and header files
*
* Copyright David Scriven, 2012-2019.
*
* Moore Laboratory, Life Sciences Institute,  2350 Health Sciences
* Mall, University of British Columbia, Vancouver, Canada, V6T 1Z3
*
* This file, tiff.cpp is part of the RYR_FIT program
*
* RYR_FIT links to the proprietary Qt system (ver 4.7) as well as the the free
* CGAL algorithmic library and the free TIFF library.
*
* This program is free software: you can redistribute it and/or modify
* it under the terms of the GNU Lesser General Public License as published by
* the Free Software Foundation, either version 3 of the License, or
* any later version.
*
* This program is distributed in the hope that it will be useful,
* but WITHOUT ANY WARRANTY; without even the implied warranty of
* MERCHANTABILITY or FITNESS FOR A PARTICULAR PURPOSE.  See the
* GNU Lesser General Public License for more details.
*
* You should have received a copy of the GNU Lesser General Public License
* along with this program.  If not, see <https://www.gnu.org/licenses/>.
******************************************************************************************/

#include "tiff.h"
#include <QMessageBox>
#include <QFileInfo>
#include "unistd.h"
#include <cstdlib>
#include <fcntl.h>

// This class allows the user to read or write multi-stack tiffs
// types: Monochrome or RGB, 8 or 16 bit
// Limited number of Fields allowed - compatible with microscopy images
// No compression
// reads tiled and line images
// writes line images
//
TiffImage::TiffImage()
{
    nFmin=0;
    nFmax=0;
    nX0=0;
    nY0=0;
    fXResolution = 1.0;
    fYResolution = 1.0;
    fXPixelSize = 0.0;
    fYPixelSize = 0.0;
    nZSpacing = 0;
    nPixelSize = 0;
    lTitle = "";

    red = nullptr;
    green = nullptr;
    blue = nullptr;

    nOffset = 0;
    nSkip = 1;
    nSamplesPerPixel = 1;
    nBitsPerSample = 8;
    nByteMultiplier = 1;
    b16bitdata = false;
    bByteImage = true;
```

```
    nPhotometric = 1;

    bReversedImage= false;
    bRGB = false;
    ByteData = nullptr;
}
TiffImage::~TiffImage()
{
    delete [] ByteData;
    delete [] red;
    delete [] green;
    delete [] blue;
}
bool TiffImage::ReadTiff(char* szFileName, char cChannelL, bool bHeaderValues)
{
    // This routine reads in TIFF files of various types:
    // 8 or 16 bit monochrome or RGB

    cChannel = cChannelL;

    QString fname = szFileName;
    QFileInfo fi(fname);
    fi.makeAbsolute();
    QString ext=fi.suffix();
    if(ext.isEmpty())
    {
        QString newname=fi.path() + ".tif";
        fi.setFile(newname);
    }
    if(!fi.exists())
    {
        QMessageBox::warning(NULL,"Error",
                             QString("File %1 does not exist!").arg(fname),
                             QMessageBox::Ok,QMessageBox::NoButton);
        return false;
    }
    if(!fi.isReadable())
    {
        QMessageBox::warning(NULL,"Error",
                             QString("File %1 is not readable - check permissions").arg(fname),
                             QMessageBox::Ok,QMessageBox::NoButton);
        return false;
    }

    fname = fi.filePath();
    tif = TIFFOpen(fname.toAscii().data(),"r");
    if(!tif)
        return false;

    uint32 width;
    uint32 length;

    // read tags in first header

    TIFFGetField(tif,TIFFTAG_IMAGEWIDTH,&width);
    TIFFGetField(tif,TIFFTAG_IMAGELENGTH,&length);

    nXdim = short(width);
    nYdim = short(length);

    TIFFGetFieldDefaulted(tif,TIFFTAG_BITSPERSAMPLE, &nBitsPerSample);
    if(nBitsPerSample == 8)bByteImage=true;
    else if (nBitsPerSample == 16)
    {
        bByteImage=false;
        nByteMultiplier=2;
    }
    else
```

```
{
    sprintf(szErrInfo," Can't handle Bits Per Sample = %d", nBitsPerSample);
    TIFFError("\n ReadTiff",szErrInfo);
    return false;
}

TIFFGetFieldDefaulted(tif,TIFFTAG_SAMPLESPERPIXEL, &nSamplesPerPixel);
if(nSamplesPerPixel == 1)
{
    bRGB = false;
    if(nBitsPerSample == 16)
        nInputType = TIFF_Mono_16bit;
    else
        nInputType = TIFF_Mono_8bit;
}
else if(nSamplesPerPixel == 3)
{
    bRGB = true;
    if(nBitsPerSample == 16)
        nInputType = TIFF_RGB_16bit;
    else
        nInputType = TIFF_RGB_8bit;
}
else
{
    sprintf(szErrInfo," Can't handle Samples Per Pixel = %d", nSamplesPerPixel);
    TIFFError("\n ReadTiff",szErrInfo);
    return false;
}

uint16 nSampleFormat;
TIFFGetFieldDefaulted(tif,TIFFTAG_SAMPLEFORMAT, &nSampleFormat);
if(nSampleFormat > 1 && nSampleFormat < 4)
{
    sprintf(szErrInfo," Can't handle Sample Format = %d", nSampleFormat);
    TIFFError("\n ReadTiff",szErrInfo);
    return false;
}

TIFFGetField(tif,TIFFTAG_PHOTOMETRIC, &nPhotometric);
if(nPhotometric != 2 && bRGB)
{
    sprintf(szErrInfo,"Photometic interpretation of %d not supported",nPhotometric);
    TIFFError("\n ReadTiff",szErrInfo);
    return false;
}

if(nPhotometric == 3)
{
    uint16* r;
    uint16* g;
    uint16* b;

    if(!TIFFGetField(tif, TIFFTAG_COLORMAP, &r, &g, &b))
    {
        TIFFError("\n ReadTiff","Missing color map!");
        return false;
    }

    delete [] red;
    delete [] green;
    delete [] blue;

    int num_entries = 1<<nBitsPerSample;
    red = new uint16[num_entries];
    green = new uint16[num_entries];
    blue = new uint16[num_entries];
    for (int i = 0; i < num_entries; i++)
    {
        red[i] = r[i];
```

```
        green[i] = g[i];
        blue[i] = b[i];
    }
    if(nBitsPerSample == 16)
        nInputType = TIFF_Map_16bit;
    else
        nInputType = TIFF_Map_8bit;
}

if(nPhotometric==0)bReversedImage=true;
else bReversedImage=false;

char* text;

if(TIFFGetField(tif, TIFFTAG_IMAGEDESCRIPTION, &text) > 0)
    Description = text;

if(TIFFGetField(tif, TIFFTAG_SOFTWARE, &text) > 0)
    SoftwareName = text;

if(TIFFGetField(tif, TIFFTAG_DATETIME, &text) > 0)
    DateandTime = text;

nFmin=GetTIFFshort(TIFFTAG_MINSAMPLEVALUE);
nFmax=GetTIFFshort(TIFFTAG_MAXSAMPLEVALUE);

uint16 nResolutionUnit;
if(TIFFGetField(tif, TIFFTAG_RESOLUTIONUNIT, &nResolutionUnit) > 0)
{
    fXResolution=GetTIFFfloat(TIFFTAG_XRESOLUTION);
    fYResolution=GetTIFFfloat(TIFFTAG_YRESOLUTION);
    if(nResolutionUnit > 1)
    {
        if(fXResolution != 0.0)
        {
            fXPixelSize = 1.e7/fXResolution;
            if(nResolutionUnit == 2)fXPixelSize *= 2.54;
        }
        if(fYResolution != 0.0)
        {
            fYPixelSize = 1.e7/fYResolution;
            if(nResolutionUnit == 2)fYPixelSize *= 2.54;
        }
    }
}

TIFFGetFieldDefaulted(tif,TIFFTAG_PLANARCONFIG , &nPlanarConfiguration);
if(nPlanarConfiguration > 1)
{
    TIFFError("\n ReadTiff","Can only handle Planar Configuration = 1");
    return false;
}

float X0 = GetTIFFfloat(TIFFTAG_XPOSITION);
if(X0 < 0.0)
    nX0 = 0;
else
    nX0 = int(X0);
float Y0 = GetTIFFfloat(TIFFTAG_YPOSITION);
if(Y0 < 0.0)
    nY0 = 0;
else
    nY0 = int(Y0);

if(!CheckDirectories())return false;

nPixelsPerPlane=nXdim*nYdim;
nPixelsPerImage=nPixelsPerPlane*nNoZPlanes;
```

```
ByteData = 0;

if(bHeaderValues)
{
    return true;
}
uint32 nBytesPerPixel = nByteMultiplier*nSamplesPerPixel;
uint32 nBytesPerLine = nBytesPerPixel*nXdim;
uint32 nBytesPerPlane = nBytesPerPixel*nPixelsPerPlane;

ByteData = new uint8 [nNoZPlanes*nBytesPerPlane];
if(ByteData == NULL)
{
    TIFFError("\n ReadTiff","Error - Out of memory for ByteData");
    TIFFClose(tif);
    return false;
}

// read data

uint8* buffer = 0;
uint32 buflen;

// Tiled TIFF

if(TIFFIsTiled(tif))
{
    uint32 tileWidth, tileLength;

    TIFFGetField(tif, TIFFTAG_TILEWIDTH, &tileWidth);
    TIFFGetField(tif, TIFFTAG_TILELENGTH, &tileLength);
    buflen = TIFFTileSize(tif);

    int tilesAcross = (nXdim + tileWidth - 1) / tileWidth;
    int tilesDown = (nYdim + tileLength - 1) / tileLength;

    buffer = new uint8[buflen];

    uint32 nBytesinTileRow = TIFFTileRowSize(tif);

    for(int jk=0; jk < nNoZPlanes; jk++)
    {
        uint8* pbplanest = ByteData + (jk+1) * nBytesPerPlane;
        for (int row = 0; row < tilesDown; row ++)
        {
            uint32 ypos = row * tileLength;
            for (int col = 0; col < tilesAcross; col ++)
            {
                uint32 xpos = col * tileWidth;

                if (TIFFReadTile(tif, buffer, xpos, ypos, 0, 0) < 0)
                {
                    sprintf(szErrInfo, "Error while reading tile at %d, %d", xpos, ypos);
                    TIFFError("\n ReadTiff", szErrInfo);
                    delete [] buffer;
                    return false;
                }

                // copy from buffer into image array

                uint8 *pbuf = buffer;
                int nNoLines = tileLength;
                int nNoBytes =  nBytesinTileRow;

                if (xpos + tileWidth > uint32(nXdim))
```

```
                nNoBytes = (nXdim - xpos)*nBytesPerPixel;
            if (ypos + tileLength > uint32(nYdim))
                nNoLines = nYdim - ypos;

            int pbOffset = nBytesPerLine+nNoBytes;
            int pbufOffset = nBytesinTileRow-nNoBytes;

            //flip image: copy from bottom to top
            uint8* pb = pbplanest - (ypos+1)*nBytesPerLine + xpos*nBytesPerPixel;

            for(int j = 0; j < nNoLines; j++)
            {
                for(int i = 0; i < nNoBytes; i++)
                    *pb++ = *pbuf++;

                pbuf += pbufOffset;
                pb -= pbOffset;
            }
          }
        }
        TIFFReadDirectory(tif);
      }
    }
    else // line TIFF
    {
        buflen = TIFFScanlineSize(tif);
        buffer = new uint8[buflen];
        int lines=nBytesPerPlane/buflen;
        for(int jk=1; jk <= nNoZPlanes; jk++)
        {
            int ipos = jk*nBytesPerPlane;
            for (int i = 0; i < lines; i++)
            {
                TIFFReadScanline(tif, buffer, i, 0);
                // flip image
                for(uint32 k = 0; k < buflen; k++)
                    ByteData[ ipos - (i+1) * buflen +k ] = buffer[k];
            }
            TIFFReadDirectory(tif);
        }
    }
    delete [] buffer;
    TIFFClose(tif);
    return true;
}
int TiffImage::check_extension(char* filename)
{
    int length = strlen(filename);
    char *p=filename+length;

    for(int j=length; j > -1; j--)
        if(*(p--) != ' ')break;

    *(p+1)='\0';

    p=strstr(filename,".tif");
    if(p==0)strcat(filename,".tif");
    else
    {
        p+=4;
        if(*p != '\0')return 35;
    }
    return 0;
}
bool TiffImage::CheckDirectories()
{
    int nNoDirs = 1;
    while (!TIFFLastDirectory(tif))
    {
        if(!TIFFReadDirectory(tif))
```

```
        {
            TIFFError("\n ReadTiff","Error while reading directory\n");
            return false;
        }
        nNoDirs++;
        int width=GetTIFFint(TIFFTAG_IMAGEWIDTH);
        int height=GetTIFFint(TIFFTAG_IMAGELENGTH);
        if(width != nXdim || height != nYdim)
        {
            cerr << " Error - width and height " << width << " x " << height;
            cerr << " in directory " << nNoDirs << " does not match that ";
            cerr << " in initial directory (" << nXdim << " x " << nYdim << ")\n";
            return false;
        }


        int bitspersample=GetTIFFshort(TIFFTAG_BITSPERSAMPLE);
        if(nBitsPerSample != bitspersample)
        {
            sprintf(szErrInfo,"Bits per sample different in image %d\n",nNoDirs);
            TIFFError("\n ReadTiff",szErrInfo);
            return false;
        }
    }


    TIFFSetDirectory(tif,0);


    nNoZPlanes = nNoDirs;
    return true;
}
float TiffImage::GetTIFFfloat(ttag_t tag)
{
    float val;
    if(TIFFGetField(tif, tag, &val) == 0)
        return 0.0;
    return (val);
}
short TiffImage::GetTIFFshort(ttag_t tag)
{
    uint16 val;
    if(TIFFGetField(tif, tag, &val) == 0)
        return short(0);
    return short(val);
}
int TiffImage::GetTIFFint(ttag_t tag)
{
    uint32 val;
    if(TIFFGetField(tif, tag, &val) == 0)
        return 0;
    return int(val);
}
void TiffImage::SetDim(short _nXdim, short _nYdim, short nZdim)
{
    nXdim = _nXdim;
    nYdim = _nYdim;
    nNoZPlanes = nZdim;
    nPixelsPerPlane=nXdim*nYdim;
    nPixelsPerImage=nPixelsPerPlane*nNoZPlanes;
}
void TiffImage::SetBitsPerSample(short bps)
{
    nBitsPerSample = bps;
    if(bps == 8)
        bByteImage=true;
    else if(bps == 16)
    {
        bByteImage=false;
        nByteMultiplier = 2;
    }
    else
    {
        cerr << " Error - can\'t set bits per sample to " << bps << "\n";
    }
```

```
}
void TiffImage::SetColourMap(uint16* r, uint16* g, uint16* b)
{
    red = r;
    green = g;
    blue = b;
}
bool TiffImage::SetOutputType(int nOutput, char cChannelL)
{
    nOutputType = nOutput;
    bByteImage = true;
    nBitsPerSample=8;
    bRGB = false;
    if(nOutput >  TIFF_Map_8bit)
    {
        bByteImage=false;
        nBitsPerSample = 16;
    }
    if(nOutputType == TIFF_RGB_8bit || nOutputType == TIFF_RGB_16bit)
    {
        bRGB=true;
        nPhotometric = 2;
        nSamplesPerPixel = 3;
        cChannel = cChannelL;
        nOffset=0;
        if(cChannel == ' ')
            nSkip = 1;
        else
        {
            nSkip = 3;
            if(cChannel == 'g')nOffset=1;
            if(cChannel == 'b')nOffset=2;
        }
    }
    if(nOutputType == TIFF_Map_8bit || nOutputType == TIFF_Map_16bit)
        nPhotometric = 3;

    if(nOutputType < TIFF_Mono_8bit || nOutputType >  TIFF_Map_16bit)
    {
        TIFFError("\n ReadTiff","Invalid output type chosen");
        return false;
    }
    return true;
}
bool TiffImage::T16bitTo8bit()
{
    uint16 *pI=reinterpret_cast <uint16*> (ByteData);
    uint16* pIst = pI;
    int nOutputSize=nPixelsPerImage*nSamplesPerPixel;
    uint8* ByteDataNew = new uint8 [nOutputSize];
    if(ByteDataNew == NULL)
    {
        TIFFError("\n 16bitTo8bit","Error - Out of memory for data");
        return false;
    }

    memset(ByteDataNew,0,nOutputSize);
    uint8* pb = ByteDataNew;

    uint16 nMax = 0;
    uint16 nMin = 65535;

    for(int i=0; i < nPixelsPerImage; i++)
    {
        if(*pI > nMax)nMax = *pI;
        if(*pI < nMin)nMin = *pI;
        pI++;
    }

    if(nMax != nMin)
    {
```

```
        float fScaling = 1.0;
        if(nMax > 256)
        {
            fScaling = 255.0 / float(nMax - nMin);
            nFmin = 0;
            nFmax = 255;
            pI = pIst;
            for(int i1=0; i1 < nPixelsPerImage; i1++)
            {
                *pb = uint8(*pI * fScaling);
                pb++;
                pI++;
            }
        }
        else
        {
            nFmin = nMin;
            nFmax = nMax;
        }
    }
    else
    {
        if (nMin > 255)nMin = 255;
        for(int i3=0; i3 < nPixelsPerImage; i3++)
        {
            *pb= uint8 (nMin);
            pb ++;
        }
        nFmin = nMin;
        nFmax = nMin;
    }
    delete [] ByteData;

    ByteData = ByteDataNew;

    return true;
}
bool TiffImage::WriteTiff(char* szFileName)
{
    if(ByteData == NULL)
    {
        TIFFError("WriteTiff","No data to write!");
        return false;
    }

    tif = TIFFOpen(szFileName,"w");
    if(!tif)
        return false;

    if(bByteImage && b16bitdata)
        T16bitTo8bit();

    TIFFSetField(tif,TIFFTAG_MINSAMPLEVALUE,  nFmin);
    TIFFSetField(tif,TIFFTAG_MAXSAMPLEVALUE,  nFmax);
    if(Description.length() != 0)
        TIFFSetField(tif,TIFFTAG_IMAGEDESCRIPTION,Description.toAscii().data());
    if(SoftwareName.length() != 0)
        TIFFSetField(tif,TIFFTAG_SOFTWARE,SoftwareName.toAscii().data());

    int nBytesPerPlane = nPixelsPerPlane * nSamplesPerPixel * nByteMultiplier;
    int nBytesPerLine = nXdim * nSamplesPerPixel * nByteMultiplier;

    for(int z=0; z < nNoZPlanes; z++)
    {
        TIFFSetField(tif,TIFFTAG_IMAGEWIDTH,nXdim);
        TIFFSetField(tif,TIFFTAG_IMAGELENGTH,nYdim);

        TIFFSetField(tif,TIFFTAG_BITSPERSAMPLE, nBitsPerSample);
        TIFFSetField(tif,TIFFTAG_SAMPLESPERPIXEL, nSamplesPerPixel);
```

```
        TIFFSetField(tif,TIFFTAG_PLANARCONFIG, uint16(1));
        TIFFSetField(tif,TIFFTAG_COMPRESSION, uint16(1));  // no compression
        TIFFSetField(tif,TIFFTAG_PHOTOMETRIC, nPhotometric);
        if(nPhotometric == 3)
            TIFFSetField(tif,TIFFTAG_COLORMAP, red, green, blue);

        TIFFSetField(tif,TIFFTAG_ORIENTATION, uint16(1));

        TIFFSetField(tif,TIFFTAG_XRESOLUTION, fXResolution);
        TIFFSetField(tif,TIFFTAG_YRESOLUTION, fYResolution);

        TIFFSetField(tif,TIFFTAG_XPOSITION, uint16(nX0));
        TIFFSetField(tif,TIFFTAG_YPOSITION, uint16(nY0));

        if(DateandTime.length() != 0)
        {
            DateandTime.truncate(20);
            TIFFSetField(tif,TIFFTAG_DATETIME, DateandTime.toAscii().data());
        }
        if(nNoZPlanes > 1)
        {
            TIFFSetField(tif, TIFFTAG_SUBFILETYPE, FILETYPE_PAGE);
            TIFFSetField(tif, TIFFTAG_PAGENUMBER , z+1, nNoZPlanes);
        }

        uint8* pbData = ByteData + z * nBytesPerPlane;

        for(int k = 0; k < nYdim; k++)
        {
            // flip image
            uint8* pbL = pbData + (nYdim - 1 - k)*nBytesPerLine;

            TIFFWriteScanline(tif, pbL, k, 0);
        }

        if(nNoZPlanes > 1)TIFFWriteDirectory(tif);
    }
    TIFFClose(tif);
    delete [] ByteData;
    return true;
}
```

**** tiff.h ****

```
/*******************************************************************************************
* Header file for tiff.cpp that reads in various types of TIFF files
*
* Copyright David Scriven, 2012-2019.
*
* Moore Laboratory, Life Sciences Institute,  2350 Health Sciences
* Mall, University of British Columbia, Vancouver, Canada, V6T 1Z3
*
* This file, tiff.h is part of the RYR_FIT program
*
* This program is free software: you can redistribute it and/or modify
* it under the terms of the GNU Lesser General Public License as published by
* the Free Software Foundation, either version 3 of the License, or
* any later version.
*******************************************************************************************/

#if !defined (_tiff_h)
#define _tiff_h

#include <fstream>
#include <iostream>
#include <cmath>
#include <sstream>
#include <stdio.h>
#include <stdlib.h>
#include <cstring>
#include <ctime>
#include <QString>
#include <QMessageBox>

using namespace std;

#include "tiffio.h"

enum {TIFF_Mono_8bit,  TIFF_RGB_8bit,  TIFF_Map_8bit,
      TIFF_Mono_16bit, TIFF_RGB_16bit, TIFF_Map_16bit};

class TiffImage
{
protected:
    TIFF*   tif;


    bool    bRGB;           // True for 3 component colour TIFF
    bool    bMap;           // True for colourmapped TIFF
    bool    bByteImage;         // True for 8 bit TIFF image
    bool    bReversedImage;     // True if BLACK is max, & white is min
    bool    bSwitchEndian;
    bool    bWrite;
    bool    b16bitdata;     // is attached data 16 bit?
    bool    bTemporaryFile;

    uint8*  ByteData;

    int     nIminVal;
    int     nImaxVal;
    int     nPixelsPerPlane;
    int     nPixelsPerImage;
    int     nBytesPerImage;
    int     nBytesPerPlane;

    int     nInputType;
    int     nOutputType;
```

```
    short   nXdim;    // Image X dim
    short   nYdim;    // Image Y dim
    short   nNoZPlanes;
    int     nX0;
    int     nY0;


    uint16* red;    // colourmap pointers
    uint16* green;
    uint16* blue;

    int     nOffset;
    int     nSkip;
    int     nByteMultiplier;


    short   nFmin;
    short   nFmax;
    float   fXResolution;
    float   fYResolution;
    float   fXPixelSize;
    float   fYPixelSize;
    int     nZSpacing;
    int     nPixelSize;


    uint16  nBitsPerSample;
    uint16  nCompression;
    uint16  nSamplesPerPixel;
    uint16  nPhotometric;
    uint16  nPlanarConfiguration;
    uint16  nResolutionUnit;


    QString SoftwareName;
    QString Computer;
    QString Description;
    QString DateandTime;
    QString ITitle;


    char    szErrInfo[256];

    char    cChannel;


public:
    TiffImage();
    ~TiffImage();

    const char*   GetErrorMsg() {return szErrInfo;}


    QString  ImageDescription() {return Description;}
    QString  Software() {return SoftwareName;}
    QString  DateTime() {return DateandTime;}
    QString  Title()  {return ITitle;}
    int      InputType() {return nInputType;}
    int      OutputType() {return nOutputType;}

    bool     WhiteisMin() {return bReversedImage;}
    short    Photometric() {return nPhotometric;}
    short    BitsPerSample() {return nBitsPerSample;}
    short    SamplesPerPixel() {return nSamplesPerPixel;}
    int      PntsPerPlane(){return nPixelsPerPlane;}
    int      PntsPerImage(){return nPixelsPerImage;}
    void     ColourMap(uint16* &r, uint16* &g, uint16* &b)
             {r = red; g = green; b = blue;}


    uint8*   BytePointer() { return ByteData;}
    uint16*  UShortPointer(){ return reinterpret_cast<uint16*> (ByteData);}

    void     SetBytePointer(uint8* pb) { b16bitdata=false; ByteData=pb;}
    void     SetUShortPointer(uint16* ps){ b16bitdata=true; nByteMultiplier=2; ByteData=reinterpret_cast<uint8*> (ps);}
```

```
    float    XPixelSize() {return fXPixelSize;}
    float    YPixelSize() {return fYPixelSize;}
    int      PixelSize() {return nPixelSize;}
    int      ZSpacing() {return nZSpacing;}
    int      X0()  { return nX0;}
    int      Y0()  { return nY0;}
    int      Xdim(){ return nXdim;}
    int      Ydim(){ return nYdim;}
    int      Zdim(){ return nNoZPlanes;}


    bool     ReadTiff(char* szInputName, char cChannel=' ', bool bHeaderValues=false);
    bool     WriteTiff(char* szOutputName);
    int      check_extension(char* filename);

    void     GetMinMax(short& Minval, short& Maxval){Minval = nFmin; Maxval = nFmax;}


    bool     SetOutputType(int nConversion, char cChannel = ' ');
    void     SetSamplesPerPixel(short spp){nSamplesPerPixel = spp;}
    void     SetBitsPerSample(short bps);
    void     SetDescription(const char* szD){Description = szD;}
    void     SetDescription(QString D){Description = D;}
    void     SetDateTime(const char* DT) {DateandTime = DT;}
    void     SetDateTime(QString DT) {DateandTime = DT;}
    void     SetSoftware(const char* Software) {SoftwareName = Software;}
    void     SetSoftware(QString Software) {SoftwareName = Software;}
    void     SetMinMax(short nMinval, short nMaxval){nFmin = nMinval; nFmax = nMaxval;}
    void     SetOrigin(short nX, short nY){nX0 = nX; nY0 = nY;}
    void     SetPhotometric(int _nPhotometric){nPhotometric = _nPhotometric;}
    void     SetColourMap(uint16* r, uint16* g, uint16* b);
    void     SetDim(short _nXdim, short _nYdim, short nZdim);
    void     SetRes(float fXRes, float fYRes){fXResolution = fXRes; fYResolution = fYRes;}


protected:


    bool     T16bitTo8bit();
    bool     CheckDirectories();
    float    GetTIFFfloat(ttag_t tag);
    short    GetTIFFshort(ttag_t tag);
    int      GetTIFFint(ttag_t tag);
};


#endif
```

**** ryr_fit3a.pro ****

```
CONFIG += debug_and_release qt
OBJECTS_DIR   = obj
MOC_DIR       = moc


debug{
DEFINES+=CGAL_DISABLE_ROUNDING_MATH_CHECK=ON
}
QMAKE_CXXFLAGS += -frounding-math


RESOURCES     = ryr_fit.qrc
HEADERS       = ryr_fit3a.h   \
                imstruct.h  \
                scale.h      \
                newscroll.h  \
                tiff.h       \
                placement.h
SOURCES       = ryr_fit3a.cpp   \
                scale.cpp      \
                tiff.cpp       \
                placement.cpp  \
                ryr_fit3_main.cpp
FORMS  =   placement.ui
LIBS  += -lCGAL -lgmp -lboost_system -ltiff
# install
TARGET = ryr_fit
QT += opengl
```

**** newscroll.h ****

```
#include <QScrollArea>

class NewScroll : public QScrollArea
{
  void keyPressEvent(QKeyEvent *e)
  {
      QApplication::sendEvent(widget(),e);
  }
  void resizeEvent(QResizeEvent *e)
  {
      QScrollArea::resizeEvent(e);
      QApplication::sendEvent(widget(),e);
  }
  void moveEvent(QMoveEvent *e)
  {
      QScrollArea::moveEvent(e);
      QApplication::sendEvent(widget(),e);
  }
  void closeEvent(QCloseEvent *e)
  {
      QWidget::closeEvent(e);
  }

public:
  NewScroll() {};
};
```

**** imstruct.h ****

```
#ifndef _imstruct_h

#define _imstruct_h
#include <QColor>

typedef unsigned short uint16;

enum {Show, DontShow, Exclude, ShowOnly};

struct Colocinfo
{
   int nRGBStatus;
   int nRGStatus;
   int nRBStatus;
   int nGBStatus;
   QColor RGBColour;
   QColor RGColour;
   QColor RBColour;
   QColor GBColour;
};

struct Imageinfo
{
   int nXdim;
   int nYdim;
   int nZdim;
   int nNoValidChannels;
   int nValidChannel[3];
   uint16 **psD;
   bool bRGB;

   int nXPixelSize;
   int nYPixelSize;
   int nZSpacing;

   int Fmax[3];
   int Fmin[3];

   QString FileName[3];
   QString Description[3];
   QString DateandTime[3];
   QString ImageType[3];
   QString Software;
};

#endif
```

**** ryr_fit3a.pro ****


```xml
<?xml version="1.0" encoding="UTF-8"?>
<ui version="4.0">
 <class>Placement</class>
 <widget class="QDialog" name="Placement">
  <property name="geometry">
   <rect>
    <x>0</x>
    <y>0</y>
    <width>224</width>
    <height>242</height>
   </rect>
  </property>
  <property name="windowTitle">
   <string>Dialog</string>
  </property>
  <widget class="QDialogButtonBox" name="bBExitCancel">
   <property name="geometry">
    <rect>
     <x>23</x>
     <y>191</y>
     <width>171</width>
     <height>32</height>
    </rect>
   </property>
   <property name="orientation">
    <enum>Qt::Horizontal</enum>
   </property>
   <property name="standardButtons">
    <set>QDialogButtonBox::Cancel|QDialogButtonBox::Ok</set>
   </property>
  </widget>
  <widget class="QLabel" name="label">
   <property name="geometry">
    <rect>
     <x>30</x>
     <y>10</y>
     <width>151</width>
     <height>20</height>
    </rect>
   </property>
   <property name="font">
    <font>
     <weight>75</weight>
     <bold>true</bold>
    </font>
   </property>
   <property name="text">
    <string>Tetramer Placement</string>
   </property>
  </widget>
  <widget class="QGroupBox" name="groupBox">
   <property name="geometry">
    <rect>
     <x>10</x>
     <y>30</y>
     <width>191</width>
     <height>171</height>
    </rect>
   </property>
   <property name="title">
    <string/>
   </property>
   <widget class="QLabel" name="lbl_nm">
    <property name="geometry">
     <rect>
      <x>148</x>
      <y>125</y>
      <width>21</width>
      <height>16</height>
     </rect>
```

```
  </property>
  <property name="text">
   <string>nm</string>
  </property>
 </widget>
 <widget class="QLineEdit" name="leOverlap">
  <property name="geometry">
   <rect>
    <x>104</x>
    <y>121</y>
    <width>41</width>
    <height>23</height>
   </rect>
  </property>
  <property name="sizePolicy">
   <sizepolicy hsizetype="Preferred" vsizetype="Fixed">
    <horstretch>0</horstretch>
    <verstretch>0</verstretch>
   </sizepolicy>
  </property>
 </widget>
 <widget class="QLabel" name="lbl_overlap">
  <property name="geometry">
   <rect>
    <x>46</x>
    <y>121</y>
    <width>56</width>
    <height>20</height>
   </rect>
  </property>
  <property name="text">
   <string>Overlap</string>
  </property>
 </widget>
 <widget class="QRadioButton" name="rbSingleTetramer">
  <property name="geometry">
   <rect>
    <x>20</x>
    <y>15</y>
    <width>145</width>
    <height>18</height>
   </rect>
  </property>
  <property name="text">
   <string>Single Tetra&amp;mer</string>
  </property>
 </widget>
 <widget class="QRadioButton" name="rbVariableOverlap">
  <property name="geometry">
   <rect>
    <x>20</x>
    <y>98</y>
    <width>145</width>
    <height>25</height>
   </rect>
  </property>
  <property name="text">
   <string>Checkerboard</string>
  </property>
 </widget>
 <widget class="QRadioButton" name="rbLaiCheckerboard">
  <property name="geometry">
   <rect>
    <x>20</x>
    <y>70</y>
    <width>145</width>
    <height>25</height>
   </rect>
  </property>
  <property name="text">
   <string>&amp;Lai Checkerboard</string>
  </property>
 </widget>
```

```xml
   <widget class="QRadioButton" name="rbLaiSidebySide">
    <property name="geometry">
     <rect>
      <x>20</x>
      <y>40</y>
      <width>145</width>
      <height>25</height>
     </rect>
    </property>
    <property name="text">
     <string>&amp;Lai side by side</string>
    </property>
   </widget>
  </widget>
  <zorder>groupBox</zorder>
  <zorder>bBExitCancel</zorder>
  <zorder>label</zorder>
 </widget>
 <resources/>
 <connections/>
</ui>
```
