# Supplementary material for: Cardiac ryanodine receptor distribution is dynamic and changed by auxiliary proteins and post-translational modification
Source: eLife. 2020 Jan 9;9:e51602. doi: 10.7554/eLife.51602 (PMC6994221; doi:10.7554/eLife.51602)
Supplement: Source code 2. [file elife-51602-code2.pdf]

Matlab program to analyse significance of CDF (NND, Alpha Shape Areas, TetramersPerCluster)

```
function testCDFdata(gtitle, Control, Phosphorylated, FKBP12, FKBP12_Phos, FKBP12_6, FKBP12_6_Phos)

[n1,~] = size(Control);
n1mean=mean(Control(:));
n1median=median(Control(:));
[n2,~] = size(Phosphorylated);
n2mean=mean(Phosphorylated(:));
n2median=median(Phosphorylated(:));
[n3,~] = size(FKBP12(:));
n3mean=mean(FKBP12(:));
n3median=median(FKBP12(:));
[n4,~] = size(FKBP12_Phos(:));
n4mean=mean(FKBP12_Phos(:));
n4median=median(FKBP12_Phos(:));
[n5,~] = size(FKBP12_6(:));
n5mean=mean(FKBP12_6(:));
n5median=median(FKBP12_6(:));
[n6,~] = size(FKBP12_6_Phos(:));
n6mean=mean(FKBP12_6_Phos(:));
n6median=median(FKBP12_6_Phos(:));

disp(' ');
disp(gtitle);
disp(['Control:  # =  ' num2str(n1) '; Mean = ' num2str(n1mean) '; Median = ' num2str(n1median)]);
disp(['Phos: # =  ' num2str(n2) '; Mean = ' num2str(n2mean) '; Median = ' num2str(n2median)]);
disp(['FKBP12: # =  ' num2str(n3) '; Mean = ' num2str(n3mean) '; Median = ' num2str(n3median)]);
disp(['FKBP12.6: # =  ' num2str(n5) '; Mean = ' num2str(n5mean) '; Median = ' num2str(n5median)]);
disp(['FKBP12 + Phos: # = ' num2str(n4) '; Mean = ' num2str(n4mean) '; Median = ' num2str(n4median)]);
disp(['FKBP12.6 + Phos: #  =  ' num2str(n6) '; Mean = ' num2str(n6mean) '; Median = '
num2str(n6median)]);
disp(' ');



ncolumns = n1 + n2 + n3 + n4 + n5 + n6;
AllData=zeros(ncolumns,2);

j = 0;
for i=1:n1
    AllData(i,1) = Control(i,1);
    AllData(i,2) = 1;
end
j = j + n1;

for i=1:n2
    AllData(j+i,1) = Phosphorylated(i,1);
    AllData(j+i,2) = 2;
end
j = j + n2;

for i=1:n3
    AllData(j+i,1) = FKBP12(i,1);
    AllData(j+i,2) = 3;
end
j = j + n3;

for i=1:n4
    AllData(j+i,1) = FKBP12_Phos(i,1);
    AllData(j+i,2) = 4;
end
j = j + n4;

for i=1:n5
    AllData(j+i,1) = FKBP12_6(i,1);
    AllData(j+i,2) = 5;
end
j = j + n5;

for i=1:n6
    AllData(j+i,1) = FKBP12_6_Phos(i,1);
    AllData(j+i,2) = 6;
end

disp('_________________________________________________________________');
disp('Comparison of all data groups');

[~,pAll]=AnDarksamtest(AllData);
%----------------------------------------------------------------------

ControlPhos=zeros(n1+n2,2);
for i=1:n1
```

```
    ControlPhos(i,1) = Control(i,1);
    ControlPhos(i,2) = 1;
end
j = n1;

for i=1:n2
    ControlPhos(j+i,1) = Phosphorylated(i,1);
    ControlPhos(j+i,2) = 2;
end

disp('_________________________________________________________________');
disp('Comparison of control & phosphorylated');

[~,pConPhos]=AnDarksamtest(ControlPhos);
%----------------------------------------------------------------------
ControlFKBP12=zeros(n1+n3,2);
for i=1:n1
    ControlFKBP12(i,1) = Control(i,1);
    ControlFKBP12(i,2) = 1;
end
j = n1;

for i=1:n3
    ControlFKBP12(j+i,1) = FKBP12(i,1);
    ControlFKBP12(j+i,2) = 2;
end

disp('_________________________________________________________________');
disp('Comparison of control & FKBP12');

[~,pConFKBP12]=AnDarksamtest(ControlFKBP12);
%-----------------------------------------------------------------------
ControlFKBP12_6=zeros(n1+n5,2);
for i=1:n1
    ControlFKBP12_6(i,1) = Control(i,1);
    ControlFKBP12_6(i,2) = 1;
end
j = n1;

for i=1:n5
    ControlFKBP12_6(j+i,1) = FKBP12_6(i,1);
    ControlFKBP12_6(j+i,2) = 2;
end

disp('_________________________________________________________________');
disp('Comparison of control & FKBP12_6');

[~,pConFKBP12_6]=AnDarksamtest(ControlFKBP12_6);

%---------------------------------------------------------------
Control_FKBP12_Phos=zeros(n1+n4,2);
for i=1:n1
    Control_FKBP12_Phos(i,1) = Control(i,1);
    Control_FKBP12_Phos(i,2) = 1;
end
j = n1;

for i=1:n4
    Control_FKBP12_Phos(j+i,1) = FKBP12_Phos(i,1);
    Control_FKBP12_Phos(j+i,2) = 2;
end

disp('_________________________________________________________________');
disp('Comparison of control & FKBP12 Phos');

[~,pConFKBP12_Phos]=AnDarksamtest(Control_FKBP12_Phos);

%---------------------------------------------------------------
Control_FKBP12_6_Phos=zeros(n1+n6,2);
for i=1:n1
    Control_FKBP12_6_Phos(i,1) = Control(i,1);
    Control_FKBP12_6_Phos(i,2) = 1;
end
j = n1;

for i=1:n6
    Control_FKBP12_6_Phos(j+i,1) = FKBP12_6_Phos(i,1);
    Control_FKBP12_6_Phos(j+i,2) = 2;
end

disp('_________________________________________________________________');
disp('Comparison of control & FKBP12.6 Phos');

[~,pConFKBP12_6_Phos]=AnDarksamtest(Control_FKBP12_6_Phos);
```

```
%---------------------------------------------------------------------
FKBP12_FKBP12_6=zeros(n3+n5,2);
for i=1:n3
    FKBP12_FKBP12_6(i,1) = FKBP12(i,1);
    FKBP12_FKBP12_6(i,2) = 1;
end
j = n3;

for i=1:n5
    FKBP12_FKBP12_6(j+i,1) = FKBP12_6(i,1);
    FKBP12_FKBP12_6(j+i,2) = 2;
end
disp('_________________________________________________________________');
disp('Comparison of FKBP12 & FKBP12_6');

[~,pFKBP12_FKBP12_6]=AnDarksamtest(FKBP12_FKBP12_6);
%--------------------------------------------------------------
Phos_FKBP12_Phos=zeros(n2+n4,2);
for i=1:n2
    Phos_FKBP12_Phos(i,1) = Phosphorylated(i,1);
    Phos_FKBP12_Phos(i,2) = 1;
end
j = n2;

for i=1:n4
    Phos_FKBP12_Phos(j+i,1) = FKBP12_Phos(i,1);
    Phos_FKBP12_Phos(j+i,2) = 2;
end

disp('_________________________________________________________________');
disp('Comparison of Phosphorylated vs FKBP12 phos');
[~,pPhos_FKBP12_Phos]=AnDarksamtest(Phos_FKBP12_Phos);

%---------------------------------------------------------------------
FKBP12_FKBP_12_phos=zeros(n3+n4,2);
for i=1:n3
    FKBP12_FKBP_12_phos(i,1) = FKBP12(i,1);
    FKBP12_FKBP_12_phos(i,2) = 1;
end
j = n3;

for i=1:n4
    FKBP12_FKBP_12_phos(j+i,1) = FKBP12_Phos(i,1);
    FKBP12_FKBP_12_phos(j+i,2) = 2;
end

disp('_________________________________________________________________');
disp('Comparison of FKBP12 vs FKBP12 phos');

[~,pFKBP12_FKBP12_Phos]=AnDarksamtest(FKBP12_FKBP_12_phos);
%---------------------------------------------------------------------
FKBP12_6_FKBP_12_6_phos=zeros(n5+n6,2);
for i=1:n5
    FKBP12_6_FKBP_12_6_phos(i,1) = FKBP12_6(i,1);
    FKBP12_6_FKBP_12_6_phos(i,2) = 1;
end
j = n5;

for i=1:n6
    FKBP12_6_FKBP_12_6_phos(j+i,1) = FKBP12_6_Phos(i,1);
    FKBP12_6_FKBP_12_6_phos(j+i,2) = 2;
end

disp('_________________________________________________________________');
disp('Comparison ofFKBP12.6 vs FKBP12.6 phos');

[~,pFKBP12_6_FKBP12_6_Phos]=AnDarksamtest(FKBP12_6_FKBP_12_6_phos);
%---------------------------------------------------------------------
Phos_FKBP12_6_Phos=zeros(n2+n6,2);
for i=1:n2
    Phos_FKBP12_6_Phos(i,1) = Phosphorylated(i,1);
    Phos_FKBP12_6_Phos(i,2) = 1;
end
j = n2;

for i=1:n6
    Phos_FKBP12_6_Phos(j+i,1) = FKBP12_6_Phos(i,1);
    Phos_FKBP12_6_Phos(j+i,2) = 2;
end

disp('_________________________________________________________________');
disp('Comparison of Phosphorylated vs FKBP12.6 phos');

[~,pPhos_FKBP12_6_Phos]=AnDarksamtest(Phos_FKBP12_6_Phos);
```

```
%-------------------------------------------------------------------
FKBP12_Phos_FKBP12_6_Phos=zeros(n4+n6,2);

for i=1:n4
    FKBP12_Phos_FKBP12_6_Phos(j+i,1) = FKBP12_Phos(i,1);
    FKBP12_Phos_FKBP12_6_Phos(j+i,2) = 1;
end
j=n4;

for i=1:n6
    FKBP12_Phos_FKBP12_6_Phos(j+i,1) = FKBP12_6_Phos(i,1);
    FKBP12_Phos_FKBP12_6_Phos(j+i,2) = 2;
end

disp('_________________________________________________________________');
disp('Comparison of FKBP12 phos & FKBP12.6 phos');
[~,pFKBP12_Phos_FKBP12_6_Phos]=AnDarksamtest(FKBP12_Phos_FKBP12_6_Phos);

disp('_________________________________________________________________');
disp('Using the Anderson-Darling k sample test (corrected for ties)');
disp (' ');
disp(checksig(['All ' gtitle],pAll));
disp(checksig('Control vs phosphorylated',pConPhos));
disp(checksig('Control vs FKBP12',pConFKBP12));
disp(checksig('Control vs FKBP12.6',pConFKBP12_6));
disp(checksig('Control vs FKBP12 phos',pConFKBP12_Phos));
disp(checksig('Control vs FKBP12.6 phos',pConFKBP12_6_Phos));
disp(checksig('FKBP12 vs FKBP12 phos',pFKBP12_FKBP12_Phos));
disp(checksig('FKBP12.6 vs FKBP12.6 phos',pFKBP12_6_FKBP12_6_Phos));
disp(checksig('FKBP12 vs FKBP12.6',pFKBP12_FKBP12_6));
disp(checksig('FKBP12 phos vs FKBP12.6 phos',pFKBP12_Phos_FKBP12_6_Phos));
disp(checksig('Phosphorylated vs FKBP12 phos',pPhos_FKBP12_Phos));
disp(checksig('Phosphorylated vs FKBP12.6 phos',pPhos_FKBP12_6_Phos));

end

function [sig] = checksig(title, pval)
sig = [title ': p = ' num2str(pval)];
if(pval < 0.05)
  sig =[sig ' - SIGNIFICANT'];
else
  sig =[sig ' - NS'];
end
end
```

The program AnDarksamtest.m was created by A. Trujillo-Ortiz, R. Hernandez-Walls, K. Barba-Rojo, L. Cupul-Magana and R.C. Zavala-Garcia, Facultad de Ciencias Marinas, Universidad Autonoma de Baja California and is not ours to share. It can be obtained from the Matlab Central file exchange.
